# Terminology for describing the 24-h cycle of physical activity, sedentary behaviour, and sleep: a scoping review

**DOI:** 10.1186/s12966-026-01945-6

**Published:** 2026-08-05

**Authors:** Guilherme M. Balbim, Ryan S. Falck, Teresa Liu-Ambrose, Chuidan Kong, David Zimmon, Daniel Elesin, Cassandra D’Amore, Jennifer C. Davis, Nucharapon Liangruenrom, Madeline Crowley, Natan Feter, Matthew N. Ahmadi, Francisco B. Ortega, Jairo H. Migueles, Jean-Philippe Chaput, Meijun Hou, Kaiqi Guan, George Thomas, Tahlia Alsop, Damian McClymont, Emmett Morgante, Rui Cao, Yujie Liu, Xun Luo, Zijun Liu, Connor Graham, Lauren Centeno Scherer, Maya Nagaishi, Linjing Zhou, Rebeca Hernández Gamboa, Yeon Soo Seo, Matthew Noseworthy, Liye Zou, Lora Giangregorio, Karen Spruyt, Sjaan R. Gomersall, Yiqun Gan, Robert Schinke, Shigeru Inoue, Takumi Abe, Yu-Kai Chang, Amy Shirong Lu, Åsa B. Tornberg, Frank Hookin Fu, Zhaowei Kong, Kirk I. Erickson, Ester Cerin, Ashleigh E. Smith, Jasper Schipperijn, Jannique van Uffelen, António Palmeira, Michelle W. Voss, David W. Dunstan, David Lubans, Linda C. Li, Jennifer R. Tomasone, Miao Miao, Keerthana Swaminathan, Varadayini Gorhe, Maurelle D’Sa

**Affiliations:** 1 Department of Physical Therapy, University of British Columbia, Faculty of Medicine, Aging, Mobility, and Cognitive Health Laboratory, Centre for Aging SMART at Vancouver Coastal Health Research Institute, Vancouver, BC Canada; 2 University of British Columbia, Faculty of Medicine, School of Biomedical Engineering, Centre for Aging SMART at the Vancouver Coastal Health Research Institute, Vancouver, BC Canada; 3 University of British Columbia-Okangan Campus, Faculty of Management, Applied Health Economics Laboratory, Kelowna, BC Canada; 4 Mahidol University, Institute for Population and Social Research, Salaya, Thailand; 5 Federal University of Rio Grande Do Sul, School of Medicine, Graduate Program in Epidemiology, Porto Alegre, RS Brazil; 6 Mackenzie Wearables Research Hub, Charles Perkins Centre and School of Health Sciences, Faculty of Medicine and Health, University of Sydney, Sydney, NSW Australia; 7 Department of Physical Education and Sports, Faculty of Sport Sciences, University of Granada, Sport and Health University Research Institute (iMUDS) and CIBEROBN Physiopathology of Obesity and Nutrition, Granada, Spain; 8 Department of Physical Education and Sports, Faculty of Sports Sciences, University of Granada, Sport and Health University Research Institute (iMUDS), Granada, Spain; 9 Department of Pediatrics, Healthy Active Living and Obesity Research Group, Children’s Hospital of Eastern Ontario Research Institute, and University of Ottawa, Ottawa, ON Canada; 10 University of British Columbia, School of Kinesiology, Vancouver, BC Canada; 11 Body-Brain-Mind Laboratory, Shenzhen University, School of Psychology, Shenzhen, Guangdong Province China; 12 The University of Queensland, Health and Wellbeing Centre for Research Innovation, School of Human Movement and Nutrition Sciences, Brisbane, Australia; 13 The University of Queensland, School of Health and Rehabilitation Sciences, Brisbane, Australia; 14 Department of Kinesiology and Health Sciences, University of Waterloo, Waterloo, ON Canada; 15 Department of Medical Psychology, Peking University, School of Health Humanities, Beijing, China; 16 The University of Western Ontario, Faculty of Health Sciences, School of Kinesiology, London, ON Canada; 17 Universidade Federal de Pelotas, School of Physical Education, Pelotas, RS Brazil; 18 Université de Paris, INSERM, Paris, NeuroDiderot France; 19 School of Psychological and Cognitive Sciences and Beijing Key Laboratory of Behavior and Mental Health, Peking University, Beijing, China; 20 Laurentian University, Faculty of Education and Health, School of Kinesiology and Health Sciences, Sudbury, ON Canada; 21 Department of Preventive Medicine and Public Health, Tokyo Medical University, 6-1-1, Shinjuku, Shinjuku-Ku, Tokyo, 160-8402 Japan; 22 School of Commerce, Meiji University, Tokyo, Japan; 23 Department of Physical Education and Sport Sciences, National Taiwan Normal University, Taipei, Taiwan; 24 Department of Communication Studies, College of Arts, Media, and Design, Department of Public Health & Health Sciences, Northeastern University, Bouvé College of Health Sciences, Health Technology Lab, Boston, MA USA; 25 Department of Health Sciences, Faculty of Medicine, Lund University, Lund, Sweden; 26 Department of Sport, Physical Education and Health, Dr. Stephen Hui Research Centre for Physical Recreation and Wellness, Faculty of Arts and Social Sciences, Hong Kong Baptist University, Kowloon Tong, Hong Kong; 27 Faculty of Education, University of Macau, Macau, China; 28 AdventHealth Research Institute, Neuroscience Institute, Orlando, FL USA; 29 Australian Catholic University-Melbourne Campus, Mary Mackillop Institute for Health Research, Behaviour, Environment and Cognition Research Program, Melbourne, VIC Australia; 30 School of Public Health, The University of Hong Kong, Hong Kong SAR, China; 31 Adelaide University, Alliance for Research in Exercise, Nutrition and Activity (ARENA) Research Centre, Adelaide, SA Australia; 32 Department of Sports Science and Clinical Biomechanics, University of Southern Denmark, Odense, Denmark; 33 Department of Movement Sciences, KU Leuven, Physical Activity, Sports & Health Research Group, Louvain, Belgium; 34 Leuven Brain Institute, KU Leuven, Louvain, Belgium; 35 CIDEFES, Universidade Lusófona, Lisbon, Portugal; 36 CIFI2D, Universidade Do Porto, Porto, Portugal; 37 Department of Psychological and Brain Sciences, The University of Iowa, Iowa Neuroscience Institute, Iowa City, IA USA; 38 Institute for Physical Activity and Nutrition, Deakin University, Burwood, Australia; 39 Baker Heart and Diabetes Institute, Melbourne, Australia; 40 Global Sport and Movement Collaborative, University of Newcastle, Callaghan, NSW Australia; 41 Hunter Medical Research Institute, New Lambton Heights, New South Wales, Australia; 42 Department of Physical Therapy, Faculty of Medicine, University of British Columbia, Arthritis Research Centre, Vancouver, BC Canada; 43 Queen’s University, School of Kinesiology and Health Studies, Revved Up Group, Kingston, ON Canada; 44 The Performance Doctor, Chennai, India; 45 Manipal Academy of Higher Education, Manipal, Karnataka India; 46 University of Staffordshire, Stoke-on-Trent, UK; 47 MindFirst Performance Psychology, Pune, India; 48 Department of Social Work and Wellbeing, Faculty of Health, Edge Hill University, Social Care and Medicine, Ormskirk, Lancashire UK

**Keywords:** Physical activity, Sedentary behaviour, Sleep, 24-h cycle, Terminology, Scoping review

## Abstract

**Background:**

Physical activity, sedentary behaviour, and sleep are three behaviours which occupy the 24-h cycle and interact to influence a multitude of health outcomes. Different terms are currently used to describe these behaviours collectively (e.g., *movement behaviours* or *physical behaviours*). The lack of clear and consistent terminology makes it hard to compare, interpret, and build on research findings. As a first step towards developing consensus on what terminology to use for discussing the 24-h cycle of physical activity, sedentary behaviour, and sleep, we conducted a scoping review to identify the breadth of terminology used to describe this phenomenon.

**Methods:**

All searches occurred between September 18th – 20th 2024. We searched nine databases: Medline, EMBASE, CINAHL, PsycInfo, Web of Science, Scopus, ERIC, Education Source, and Compendex from inception to September 18th 2024. The Journal of Activity, Sedentary and Sleep Behaviors and the International Network of Time-Use Epidemiologists (INTUE) publication lists were manually searched on September 19th – 20th 2024. Peer-reviewed articles were included if they 1) were published in English, Spanish, Portuguese, French, Chinese, Korean, or Japanese; 2) focused on humans; and 3) describe physical activity, sedentary behaviour, and sleep in an integrated manner. We identified different terminologies used for collectively discussing physical activity, sedentary behaviour, and sleep across the 24-h cycle.

**Results:**

Our search identified 10,749 articles of which 979 articles met our inclusion criteria. Across all articles, 199 different terms were used to describe physical activity, sedentary behaviour, and sleep collectively. The term *movement behaviours* was used in 673 articles, *24-h movement behaviours* was used in 457 articles, *physical behaviours* was used in 58 articles, *24-h activity cycle* was used in 36 articles, and *time-use behaviours* was used in 31 articles.

**Conclusions:**

The two most popular terms for describing the collective interaction of physical activity, sedentary behaviour, and sleep are *movement behaviours* and *24-h movement behaviours*. However, there is not yet an internationally recognized and agreed-upon term for holistically describing the 24-h cycle of physical activity, sedentary behaviour, and sleep. Developing a homogeneous and widely accepted set of terminology and definitions is thus critical if we hope to ensure clarity, enhance reproducibility, and enable the translation of future research into coherent guidelines, policies, and practices for these behaviours.

**Supplementary Information:**

The online version contains supplementary material available at https://doi.org/10.1186/s12966-026-01945-6.

## Background

Clear, concise, and consistent terminology is crucial for effective science communication and knowledge translation. Using scientific terminology which is clear, concise, and consistent ensures construct uniformity, encourages the synthesis of knowledge by enabling easy comparisons between study findings, and enhances the communication of scientific discoveries and important public health information to both the academy and different interest-holder groups [1].

Health research has a growing interest in the inter-relationships of physical activity, sedentary behaviour, and sleep [2]. These behaviours occupy the 24-h cycle and are associated (both individually and synergistically) with numerous health outcomes, such as cardiovascular disease, cognition, obesity, academic performance, and mortality [3–7]. Consensus statements have been developed for determining how these behaviours should be analyzed [8]. There are also behavioural guidelines for physical activity, sedentary behaviour, and sleep which now exist in multiple countries and population groups [9–12]. Guidelines for children have primarily focused on screen time [9–12], a type of sedentary behaviour [13].

There is a well-defined vocabulary for each these behaviours. Physical activity is commonly defined as any bodily movement produced by skeletal muscles which causes energy expenditure [14]. Conversely, sedentary behaviour is defined as any waking behaviour performed from a seated/lying position which incurs less than or equal to 1.5 metabolic equivalents (METs) [15]. Sleep is defined as a recurring, reversible, neurobehavioural state characterized by reduced responsiveness to external stimuli and disengagement from the environment, and in humans is typically accompanied by postural recumbence, behavioural quiescence, and closed eyes [16].

While researchers have often treated physical activity, sedentary behaviour, and sleep as isolated behaviours, many now recognize that these behaviours share an interactive relationship with each other and health [2–7, 17]. There are now device-based methods capable of concurrently observing physical activity, sedentary behaviour, and sleep, and analytic methods capable of examining how these behaviours holistically interact with health and well-being [18–21]. Studies using these methods point to a dynamic and complex relationship between physical activity, sedentary behaviour, and sleep and multiple health outcomes [2]. Physical activity, sedentary behaviour, and sleep are also linked to circadian rhythms – the 24-h biological clock that regulates the sleep–wake cycle [19].

Some classifications of the 24-h cycle of physical activity, sedentary behaviour, and sleep also include standing behaviour as a separate behaviour within this cycle [15, 17, 22]. Standing behaviour refers to a position in which one has or is maintaining an upright position while supported by one’s feet [22], and incurs between 1.0 and 1.5 METs [23]; light physical activity incurs between 1.5 and 3.0 METs, and moderate-to-vigorous physical activity incurs ≥ 3.0 METs [19]. Standing behaviour is sometimes classified as a type of light physical activity [17], although it has similar energy expenditure as sedentary behaviour [24]. Hence, there is a critical need to develop a clear terminology framework for discussing these behaviours in an integrated manner.

The way that we collectively refer to these behaviours is inconsistent. There are at least five separate terms used by researchers, including: 1) *movement behaviours*; 2) *physical behaviours*; 3) *time-use behaviours*; 4) *time-use activity behaviours*; and 5) *the 24-h activity cycle* [25]. However, there has yet to be a systematic examination of these terms across the literature, and it is likely that there are more variations.

Thus, there is a lack of consistent and clear terminology to discuss the 24-h cycle of physical activity, sedentary behaviour, and sleep which has become more magnified as this line of research inquiry increases in popularity [25]. If this issue is not addressed, it will impede the synthesis of new knowledge about how these behaviours collectively impact health, and potentially lead to public health guidelines which use inconsistent and confusing terminology which will reduce the uptake of intended messages. While a consensus on what terminology should be used for discussing this 24-h cycle is surely needed [25], a key first step towards developing consensus is a review of the current terminology used to describe this phenomenon across the field [26, 27]. Therefore, we conducted a scoping review to identify the breadth of terminology used to collectively discuss physical activity, sedentary behaviour, and sleep. As a secondary aim we explored how standing behaviour was incorporated and discussed within the contexts of this 24-h cycle.

## Methods

### Protocol and reporting

Our scoping review protocol is registered and publicly accessible with the Open Science Framework (Registration DOI: https://doi.org/10.17605/OSF.IO/5PJE4; Registration Date: September 24, 2024) and was developed using the scoping review methodological framework proposed by Arksey and O’Malley [28] and further refined by the Joanna Briggs Institute (JBI) [29]. No amendments were made to the registered protocol. We conducted this scoping review following the Preferred Reporting Items for Systematic Reviews and Meta-Analyses extension for Scoping Reviews (PRISMA-ScR) [30].

### Eligibility criteria

We included peer-reviewed articles which met the following criteria: 1) published in English, Spanish, Portuguese, French, Chinese, Korean, or Japanese (i.e., languages spoken by our research team); 2) focused on humans; and 3) examined or discussed the behaviours of physical activity, sedentary behaviour, and sleep, and their interaction. We did not impose restrictions on age range, outcomes of interest, or study design. We excluded articles if they were: 1) gray literature (i.e., dissertations or theses, conference proceedings and abstracts, books and book chapters, and reports); 2) non-human research; 3) did not discuss or examine all three behaviours (i.e., physical activity, sedentary behaviour, and sleep); and 4) did not clearly acknowledge that all three behaviours were interrelated, or only examined these behaviours independently.

### Information sources and search strategy

The search strategy was developed collaboratively (GMB, RSF, JCD, TLA) and with the help of an experienced librarian. The search strategy terms were grouped into four main concepts: 1) physical activity; 2) sedentary behaviour; 3) sleep; and 4) the collective discussion of these three behaviours. The search strategy accounted for potential differences between Canadian, American, and British English (e.g., behaviour *vs*. behavior) and between singular and plural forms (e.g., behaviour *vs*. behaviours). The complete search strategy is available in Supplementary Material S1. All searches were conducted between September 18th – September 20th, 2024. We identified relevant articles via five sources: a) electronic databases (Scopus, Web of Science, PsycInfo, Medline, EMBASE, CINAHL, ERIC, Education Source, and Compendex) from inception to September 18th 2024; b) manual searches of the *Journal of Activity, Sedentary and Sleep Behavior* – which at the time of the scoping review search had yet to be indexed in PubMed Central – given the relevance of its scope to our scoping review on September 19th 2024; c) manual search of International Network of Time-Use Epidemiologists (INTUE) publications list on September 20th 2024, given the relevance of the Network’s mission; and d) from previous knowledge and input from co-authors. In our protocol, we initially proposed conducting forward manual searches (searching the references of included papers). However, we did not conduct forward manual searches, given the practical constraints of reviewing bibliographies for all 979 included articles.

### Study selection

Our data screening and extraction team included 33 reviewers from Australia (DM, GT, TAlsop, MNA), Brazil (LCS, NF), Canada (CA, CK, CG, DE, DZ, EM, GMB, JPC, MC, MNoseworthy, RSF, RHG, YSS) China (CR, KG, LZ, MH, XL, YL, ZL), France (MNagaishi), Japan (SI, TAbe), Spain (FBO, JHM), Taiwan (YKC), and Thailand (NL). We imported retrieved references into Covidence (Veritas Health Innovation, Melbourne, Australia) and removed duplicates. Reviewers underwent a pilot screening with a random sample of ten titles and abstracts, and five full-text screenings, and were required to achieve an accuracy rate of ≥ 75% before screening articles. The median agreement between reviewers for full text review was 63%. A third independent reviewer resolved conflicts in the title/abstract and full-text screening stages. All articles were screened and reviewed by reviewers who were native speakers or had academic proficiency in the language of the text (i.e., English, Spanish, Portuguese, French, Chinese, Korean, and Japanese). Articles in English were reviewed by DM, GT, TAlsop, MA, NF, CA, CK, CG, DE, DZ, EM, GMB, MC, RSF, YSS, CR, KG, LZ, MH, XL, YL, ZL, and NL. Spanish language articles were reviewed by FBO, JMH, and RHG. Articles in Portuguese were reviewed by LCS, NF, and GMB. French articles were reviewed by MNoseworthy, JPC, and MNagaishi. Chinese articles were reviewed by CK, CR, KG, LZ, MH, XL, YL, and ZL. Articles written in Korean were reviewed by YKC, YSS, and SI, and articles in Japanese were reviewed by TAbe, SI, and YKC. If a third reviewer was required to resolve conflicts, then this individual was also academically fluent in the language of the text. The screening was conducted using Covidence, a web-based collaboration software that streamlines the production of systematic and other literature reviews, without the assistance of automation tools.

### Data extraction

We created a data extraction form in Covidence to extract relevant information from each article. All data extractors undertook a pilot data extraction using a random sample of five included articles and were required to achieve an accuracy rate of ≥ 75% before extracting data from the included articles. The following data were extracted: 1) author(s) and date; 2) language; 3) geographical location (i.e., of the research team and study); 4) study type (e.g., quantitative, qualitative, mixed-methods); 5) research design (e.g., cross-sectional, cohort, randomized controlled trial); 6) settings (e.g., community, school, workplace, clinical); 7) participants’ characteristics (e.g. sample size, mean age, proportion of females); 8) participants’ health status (e.g., healthy, individuals living with chronic diseases, etc.); 9) terminology adopted to discuss physical activity, sedentary behaviour, and sleep as independent behaviours; 10) reporting of standing behaviour; 11) terminology adopted to collectively discuss physical activity, sedentary behaviour, and sleep throughout the 24-h cycle; and 12) outcomes of interest. We determined the different geographic locations of all members of the research team for a given study based on the author contact details from each manuscript. To extract data on articles which discussed standing behaviour, we first classified articles based on whether or not they reported or measured standing behaviour (yes/no). If standing behaviour was measured, we then extracted how standing behaviour was measured, and subsequently determined if standing behaviour was considered a separate component of the 24-h day (i.e., joint interrelationship with physical activity, sedentary behaviour, and sleep).

Variations in spelling (e.g., American versus British English) were grouped under a single term. For example, the terms *sedentary behavior* and *sedentary behaviour* were both categorized as the term *sedentary behaviour*. Singular and plural versions of a term were also classified as a single term. For instance, *sedentary behaviour* and *sedentary behaviours* were both classified as the term *sedentary behaviour*. Among studies published in languages other than English, data were extracted by reviewers who were native speakers or had academic proficiency in both the source language and English. Terminology reported in the original articles was translated into English during data extraction to enable consistent synthesis across studies. Reviewers aimed to capture the closest conceptual equivalent rather than applying literal word-for-word translation, prioritizing terminology already used in the English-language literature when appropriate. During synthesis, conceptually equivalent terms were grouped to avoid counting minor linguistic variations as distinct terminology. This approach allowed integrating terminology from multiple languages while maintaining a consistent analytical framework.

### Data synthesis, analysis and reporting

Following the Arksey and O’Malley [28] framework, we conducted an evidence-mapping step to organize and summarize the extracted data prior to narrative synthesis. The purpose of this process was to examine how terminology used to collectively discuss physical activity, sedentary behaviour, and sleep was distributed across study characteristics (i.e., continent, type of outcome, language) and participant characteristics (i.e., age-group, health status). We also examined how terminology was used to discuss standing behaviour, and whether standing behaviour was considered as a separate component of the 24-h day. These variables were summarized descriptively to illustrate how terminology varied across methodological approaches, settings, and populations.

Data were analyzed using R version 4.3.3 (2024–02–29). As this was a scoping review, analyses were limited to summary statistics. Categorical and nominal variables (i.e., study type, research design, setting, outcomes of interest, participants’ health status, reported proportion of females, age range, terminology, and reporting of standing behaviour) were summarized using frequencies and percentages. Continuous descriptive variables reported at the study level (i.e., sample size, mean age) were summarized using means and standard deviations.

### Data availability statement

The review database (coding sheets, and R scripts) is openly available at the Open Science Framework: https://osf.io/s8anf/overview.

## Results

**Fig. 1:**
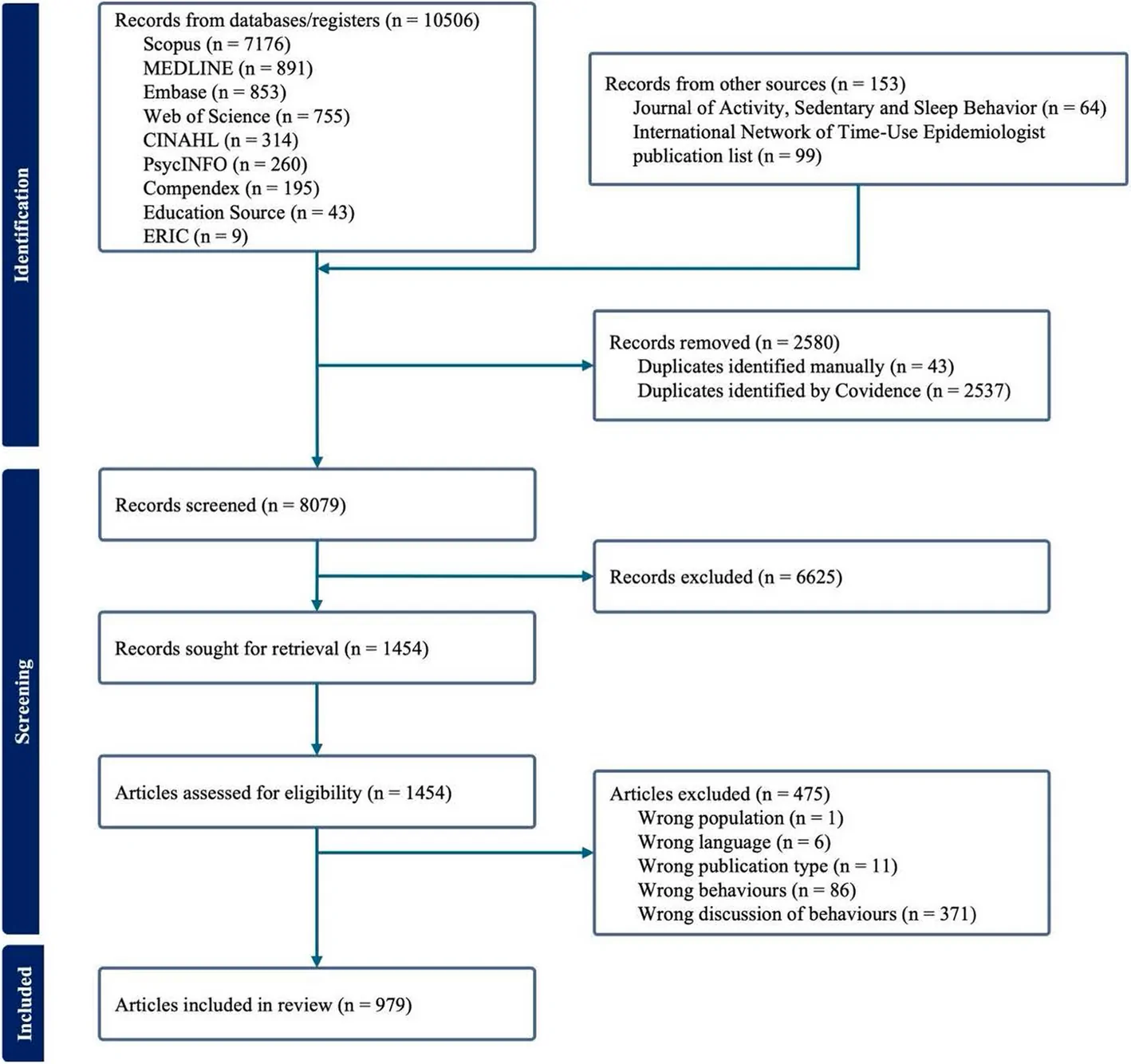
Preferred Reporting Items for Systematic Reviews and Meta-Analyses extension for Scoping Reviews (PRISMA-ScR) flow diagram

Figure 1 describes our PRISMA-ScR flow chart. The initial search identified 10,749 articles. Of these, 10,596 articles were identified in databases and 153 from other sources (i.e., Journal of Activity, Sedentary and Sleep Behaviors and INTUE publication lists). A total of 2,580 duplicates were removed. We assessed the eligibility of 8,079 titles and abstracts, of which 1,454 articles underwent full-text review. A total of 979 articles were included in the scoping review (Fig. 1). The bibliography of included articles is reported in Supplementary Material S2.

### Article characteristics

We describe article characteristics in Supplementary Material S3. Of the 979 included articles, there were 796 empirical research articles (81.3%), 94 reviews (e.g., systematic reviews, scoping reviews, narrative reviews [9.6%]), 33 commentaries or editorials (3.4%), 24 protocol articles (2.5%), 14 measurement studies (e.g., validity and reliability, questionnaire development [1.4%]), 8 expert consensus statements (0.8%), 8 guideline development articles (0.8%). There were 2 articles which we classified as other study designs (e.g., integrated knowledge translation and policy analysis [0.2%]). The empirical research articles were cross-sectional analyses (*n* = 629 [64.2%]), longitudinal case–control or cohort designs (*n* = 122 [12.5%]), combined cross-sectional and longitudinal designs (*n* =19 [1.9%]), randomized controlled trials (*n* = 18 [1.8%]), quasi-experimental studies (*n* = 3 [0.3%]), and other study designs (*n* = 5 [0.5%], e.g., Mendelian randomization, one-group pretest–posttest, and qualitative study). The review articles comprised systematic reviews without meta-analysis (*n* = 39 [4.0%]), scoping reviews (*n* = 26 [2.7%]), systematic reviews with meta-analysis (*n* = 22 [2.2%]), narrative reviews (*n* = 5 [0.5%]), a documentary review (*n* = 1 [0.1%]), and an umbrella review (*n* = 1 [0.1%]).

**Fig. 2:**
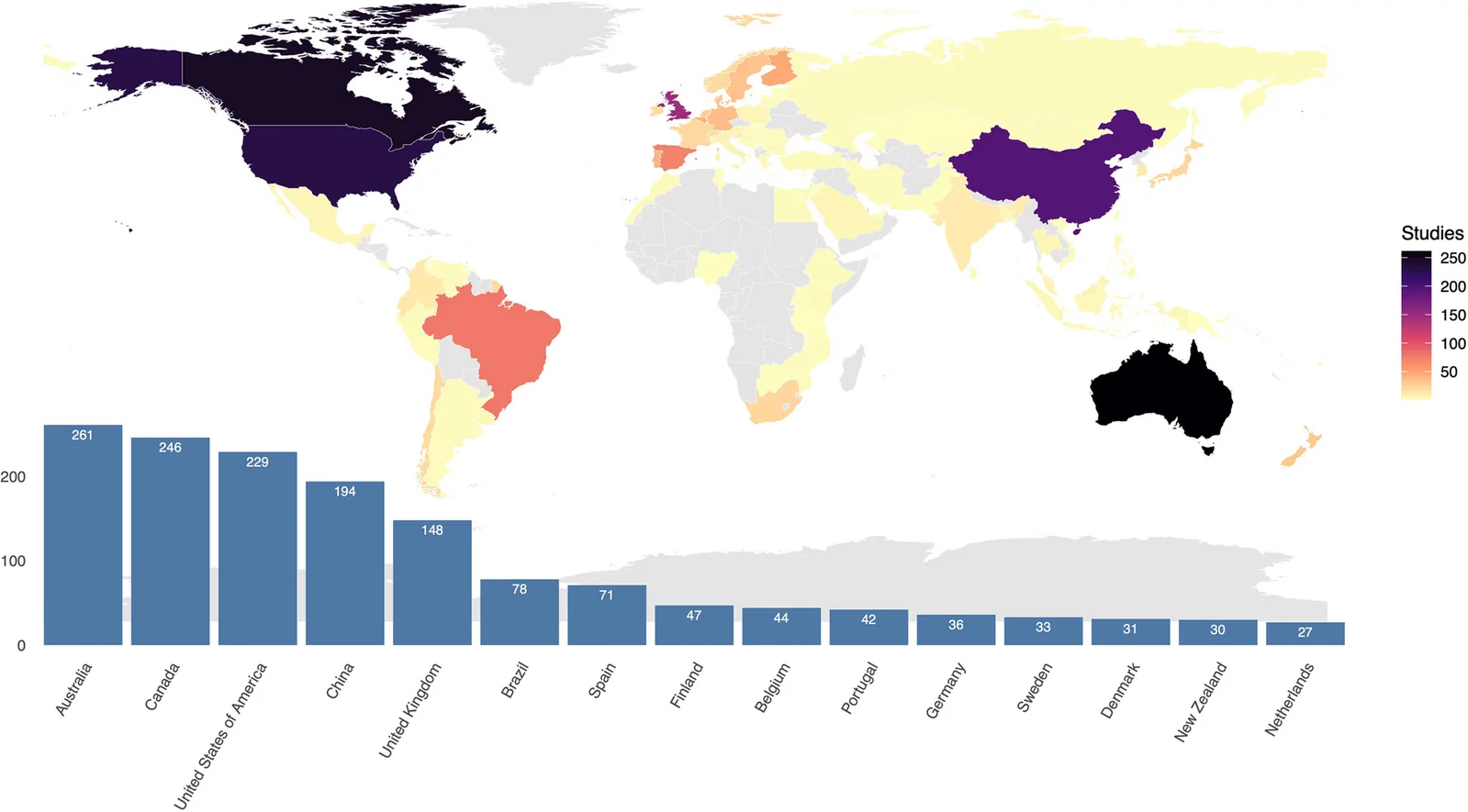
Number of articles collectively discussing physical activity, sedentary behaviour, and sleep throughout the 24-h cycle per country. The bar plot depicts the top 15 countries

**Fig. 3:**
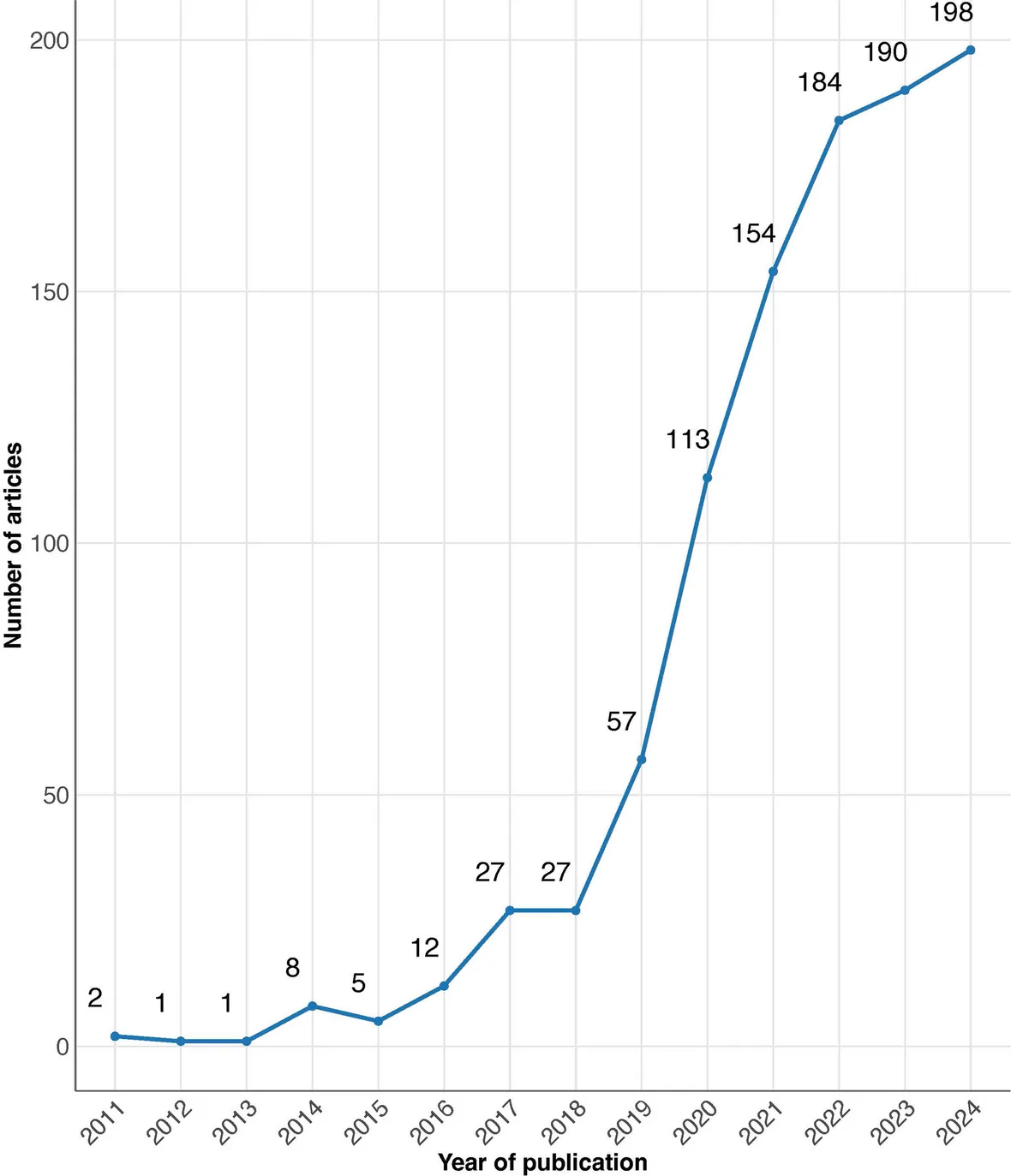
Number of published articles collectively discussing physical activity, sedentary behaviour, and sleep throughout the 24-h cycle per year

Empirical research articles were conducted in 99 different countries/regions, with the most studies conducted (i.e., data were collected) in the United States (*n* = 218 [22.3%]), Canada (*n* = 177 [18.1%]), and China (*n* = 176 [18.0%]). One hundred and fifteen articles (15.3%) collected data from multiple countries/regions. Members of the research teams were from 92 different countries/regions (Fig. 2). Across the 979 included studies, 261 studies included researchers from Australia (26.7%), 246 included researchers from Canada (25.1%), 229 included researchers from the United States of America (23.4%), 194 included researchers from China (15.1%), and 148 included researchers from the United Kingdom (15.1%). Included articles were published from 2011 (*n* = 2 [0.2%]) to 2024 (*n* = 198 [20.2%]) (Fig. 3). The number of articles published each year steadily increased over the 13-year period and increased at an exponential rate after 2018. A total of 932 articles were published in English (95.2%), 34 published in either traditional or simplified Chinese (3.5%), seven in Spanish (0.7%), three in Portuguese (0.3%), and one article each in French (0.1%), Japanese (0.1%), and Korean (0.1%).

Of the 979 included articles, 847 reported sample sizes (86.5%), comprising a total of more than 61 million individuals (range: 10 to 23,431,351). There were 975 studies which reported age; the mean age was 25.75 ± 22.16 years (range: 2 months to 85.30 years). A total of 300 articles included children (0–14 years old [30.6%]), 245 included children (0–14 years old) and youth (15–24 years old [25.0%]), 96 included adults (25–64 years old) and older adults (≥ 65 years old [9.8%]), 74 included youth, adults, and older adults (7.6%), 56 included adults (5.7%), 53 included youth and adults (5.4%), 44 included youth (4.5%), 27 included older adults (2.8%), 17 included children, youth, adults, and older adults (1.7%), 11 included children, youth, and adults (1.1%), 10 included children and adults (1.0%), and 2 included children, youth and older adults (0.2%); 40 articles could not be classified by age group (4.1%). A total of 753 articles reported biological sex (76.9%). Of these, 502 reported biological sex as male and female (51.3%), 263 as boys and girls (26.9%), and 18 as male, female, and other (1.8%). The mean reported proportion of females was 56.6 ± 16.4%, 49.9 ± 9.1% for girls, and 5.9 ± 13.2% for other sex. Only 225 articles reported the health status of participants (23.0%). Eighty-three articles examined healthy individuals (8.5%), 24 investigated people with overweight or obesity (2.5%), 14 examined people with autism spectrum disorder (1.4%), and 14 were among people living with physical disabilities (1.4%).

Three hundred fifty-six articles (36.4%) examined physical outcomes of interest, 284 articles (67.3%) investigated psychosocial or brain health-related outcomes, and 50 examined other behaviours as outcomes of interest (5.1%). The most common physical outcomes were body mass index (*n* = 227 [23.2%]), adiposity (*n* = 226 [23.1%]), and weight (*n* = 226 [23.1%]). Depression (*n* = 85 [8.7%]), cognition (*n* = 65 [6.6%]), and anxiety (*n* = 46 [4.7%]) were the most common psychosocial or brain health-related outcomes. Other behavioural outcomes included diet (*n* = 42 [4.3%]), tobacco consumption (*n* = 13 [1.3%]) and alcohol consumption (*n* = 11 [1.1%]).

### Terminology used to discuss physical activity, sedentary behaviour, and sleep

A complete list of the terminology used to discuss the constructs of physical activity, sedentary behaviour, and sleep as individual behaviours is included in Supplementary Material S4. Across the 979 articles, the construct of physical activity was referred to according to time, modality, and domain characteristics using 68 distinct terms and primarily referred to as *physical activity* (*n* = 979 [100%]), *exercise* (*n* = 132 [13.5%]), or *physical exercise* (*n* = 19 [1.9%]). One hundred and seventy-two articles used two distinct terms for the construct of physical activity (17.6%); 51 used three or more (5.2%). The most common terms used to discuss the construct of physical activity did not vary by age group and health status.

The included articles employed 62 different terms to refer to the construct of sedentary behaviour. These terms described the construct of sedentary behaviour according to time, type, and domain characteristics. The most commonly adopted terms were *sedentary behaviour* (*n* = 883 [90.2%]), *screen time* (*n* = 569 [58.1%]), *sedentary time* (*n* = 488 [49.9%]), or *sitting time* (*n* = 170 [17.4%]). A total of 381 articles (38.9%) adopted two distinct terms and 446 articles (45.6%) used three or more terms for the construct of sedentary behaviour. The most common terms used to discuss the construct of sedentary behaviour varied by age group, such that *screen time* was the second most common term in articles which included children and youth. The most common terms did not vary by health status.

The construct of sleep was referred to using 17 distinct terms regarding time and type (e.g., nap) characteristics. The most frequent being *sleep* (*n* = 972 [99.28%]), *sleep duration* (*n* = 706 [72.11%]), or *sleep time* (*n* = 460 [46.99%]). There were 474 articles which used two different terms for the construct of sleep (48.4%), and 338 articles using three or more (34.5%). The most common terms to describe the construct of sleep did not vary by age group and health status.

### Standing behaviour as a component of the 24-h cycle of physical activity, sedentary behaviour, and sleep

Standing behaviour was measured or reported in 98 articles (10.0%). Among these articles, 65 articles (6.7%) discussed standing behaviour as a separate component of the 24-h day. The terminology used in articles discussing standing behaviour were also similar to those used in articles that did not measure or report standing behaviour. For example, among the 65 articles which discussed standing behaviour as a separate and interrelated component of the 24-h day, the two most popular terms for collectively discussing physical activity, sedentary behaviour, sleep, and standing behaviour were *movement behaviours* (*n* = 33; 50.8%) and *24-h movement behaviours* (*n* = 21; 32.3%). Among the 881 articles which did not measure or report standing behaviour, the two most popular terms were also *movement behaviours* (*n* = 611; 69.4%) and *24-h movement behaviours* (*n* = 424; 48.1%).

### Terminology used to describe the 24-h cycle of physical activity, sedentary behaviour, and sleep

The included articles employed a variety of distinct terms (*n* = 199, see Supplementary Material S5 for a complete list of terms) to collectively discuss physical activity, sedentary behaviour, and sleep. The most commonly adopted terms were: 1) *movement behaviours* which was used in 673 articles (68.7%); 2) *24-h movement behaviours* used in 457 articles (46.7%); 3) *physical behaviours* in 58 articles (5.9%); 4) *24-h activity cycle* in 36 articles (3.7%); and 5) time-use behaviours used in 31 articles (3.2%). Seven hundred and thirty-six articles (75.2%) used either *movement behaviours* or *24-h movement behaviours*, and 384 articles (39.2%) used both terms.

Among English-language articles, 196 distinct terms were used to collectively discuss physical activity, sedentary behaviour, and sleep. The most commonly adopted terms were: 1) *movement behaviours* which was used in 654 articles (74.2%); 2) *24-h movement behaviours* used in 436 articles (49.5%); 3) *physical behaviours* in 54 articles (6.1%); 4) *24-h activity cycle* in 34 articles (3.9%); and 5) *time-use behaviours *used in 31 articles (3.5%). Seven hundred and twelve articles (80.8%) used either *movement behaviours* or *24-h movement behaviours*, and 378 articles (42.9%) used both terms.

Across non-English languages, 18 terms were used to collectively discuss physical activity, sedentary behaviour, and sleep. Articles in Chinese used 15 terms. Spanish-language articles used 4, Portuguese used 3, Japanese used 2, and Korean used 1. The article in French did not use a specific term for describing these behaviours concurrently. The most commonly adapted terms were *movement behaviours* or *24-h movement behaviours*. The one exception was Chinese, wherein the term *24-h movement behaviours* (*n* = 14/34; 41.2%) was the most commonly adapted term, followed by: *24-h activity behaviours* (*n* = 5/34; 14.7%), *movement behaviours* (*n* = 3/34; 8.8%), and *physical behaviours* (*n* = 2/34, 5.9%).

Articles with distinct participants’ age group (e.g., children, youth, adults, older adults, and combinations of different age groups), participants’ health status (e.g., healthy, living with a disease), and health outcomes (e.g., physical, psychosocial, and behavioural), also used *movement behaviours* and *24-h movement behaviours* more frequently (Table 1). With regards to where the research teams were located, the most popular terms across all continents were also *movement behaviours* and *24-h movement behaviours*. Of the 438 articles published by teams with North American researchers, 315 (71.9%) used the term *movement behaviours*; 202 (46.1%) used the term *24-h movement behaviours*. The terms *movement behaviours* and *24-h movement behaviours* were used in the 2 articles published by teams with researchers located in Central America (100.0%). Among the 109 articles published by teams with researchers located in South America, 81 (74.3%) used the term *movement behaviours*, and 72 (66.1%) used the term *24-h movement behaviours*. For articles published by teams with researchers located in Europe, 266/367 (72.5%) used the term *movement behaviours*; there were 190/367 articles (51.8%) which used the term *24-h movement behaviours*. Among researchers located in Asia, 175/266 articles (65.8%) published used the term *movement behaviours*, and 152/266 (57.1%) used the term *24-h movement behaviours*. For the 278 articles published by teams with researchers located in Oceania, 190 (68.3%) used the term *movement behaviours*, while 115 used the term *24-h movement behaviours* (41.4%). For articles published by teams with researchers located in Africa (*n* = 31), 25 articles (80.6%) used the term *movement behaviours*; there were 6 (48.4%) which used the term *24-h movement behaviours*.

**Table 1:** Frequency of the five most popular terms adopted to discuss physical activity, sedentary behaviour, and sleep collectively by age-group, type of outcome of interest, health status, continent, and language of publication

**Age group**^**a**^	**24-h terminology reported**	**Articles reporting (n)**	**% of articles**
Children (*n* = 356)	Movement behaviours	235	78.1
	24-h movement behaviours	151	50.2
	Time-use compositions	10	3.3
	Physical behaviours	8	2.7
	Time-use behaviours	8	2.7
Children and youth (*n* = 245)	Movement behaviours	176	71.8
	24-h movement behaviours	133	54.3
	24-h activity cycle	5	2.0
	24-h activity behaviour	4	1.6
	Lifestyle behaviours	4	1.6
Youth (*n* = 44)	Movement behaviours	26	59.1
	24-h movement behaviours	24	54.5
	24-h activity cycle	4	9.1
	24-h activity behaviour	2	4.5
	24-h cycle	2	4.5
Adults (*n* = 57)	Movement behaviours	38	66.7
	24-h movement behaviours	21	36.8
	Physical behaviours	6	10.5
	24-h activity cycle	3	5.3
	24-h time-use	3	5.3
Older adults (*n* = 27)	Movement behaviours	12	44.4
	24-h movement behaviours	8	29.6
	24-h activity cycle	4	14.8
	Time-use behaviours	4	14.8
	24-h behaviours	3	11.1
**Type of outcome**^**a**^	**24-h terminology reported**	**Articles reporting (n)**	**% of articles**
Physical (*n* = 247)	Movement behaviours	155	62.8
	24-h movement behaviours	106	42.9
	Physical behaviours	25	10.1
	Time-use compositions	15	6.1
	24-h time-use compositions	12	4.9
Psychosocial (*n* = 181)	Movement behaviours	136	75.1
	24-h movement behaviours	106	58.6
	Time-use behaviours	13	7.2
	Time-use pattern	6	3.3
	Lifestyle behaviours	5	2.8
Behavioural (*n* = 22)	Movement behaviours	19	81.8
	24-h movement behaviours	12	54.5
	Time-use behaviours	2	9.1
	24-h integrated lifestyle behaviours	1	4.5
	24-h lifestyle behaviours	1	4.5
**Health status**^**a**^	**24-h terminology reported**	**Articles reporting (n)**	**% of articles**
Living with disease (* n* = 132)	Movement behaviours	86	62.1
	24-h movement behaviours	68	51.5
	24-h activity cycle	10	7.6
	Physical behaviours	10	7.6
	24-h activities	5	3.8
Healthy (*n* = 64)	Movement behaviours	44	68.8
	24-h movement behaviours	31	48.4
	24-h time-use compositions	7	10.9
	Time-use pattern	4	6.3
	Physical behaviours	3	4.7
Pregnant (*n* = 9)	Movement behaviours	5	55.6
	24-h movement behaviours	4	44.4
	24-h activity behaviours	1	11.1
	24-h activity compositions	1	11.1
	24-h activity cycle	1	11.1
**Continent of research team**	**24-h terminology reported**	**Articles reporting (n)**	**% of articles**
North America (*n* = 438)	Movement behaviours	315	71.9
	24-h movement behaviours	202	46.1
	24-h activity cycle	27	6.2
	Physical behaviours	12	2.7
	Time-use compositions	10	2.3
Central America (*n* = 2)	24-h movement behaviours	2	100.0
	Movement behaviours	2	100.0
	Movement behaviours	81	74.3
South America (*n* = 109)	24-h movement behaviours	72	66.1
	24-h activity cycle	5	4.6
	Physical behaviours	4	23.7
	Time-use compositions	4	3.7
Europe (*n* = 367)	Movement behaviours	266	72.5
	24-h movement behaviours	190	51.8
	Physical behaviours	38	10.4
	24-h time-use compositions	19	5.2
	Time-use compositions	18	4.9
Asia (*n* = 266)	Movement behaviours	175	65.8
	24-h movement behaviours	152	57.1
	24-h activity behaviours	10	3.8
	Physical behaviours	9	3.4
	Time-use behaviours	8	3.0
Oceania (*n* = 278)	Movement behaviours	190	68.3
	24-h movement behaviours	115	41.4
	Time-use behaviours	18	6.5
	24-h time-use	17	6.1
	Time-use compositions	16	5.8
Africa (*n* = 31)	Movement behaviours	25	80.6
	24-h movement behaviours	15	48.4
	Daily activity behaviours	3	9.7
	Lifestyle behaviours	2	6.5
	Physical behaviours	1	3.2
**Language of publication**^**b**^	**24-h terminology reported**	**Articles reporting (n)**	**% of articles**
English (*n* = 932)	Movement behaviours	654	70.2
	24-h movement behaviours	436	46.8
	Physical behaviours	54	5.8
	24-h activity cycle	34	3.6
	Time-use behaviours	31	3.3
Chinese (*n* = 34)	24-h movement behaviours	14	41.2
	24-h activity behaviours	5	14.7
	Movement behaviours	3	8.8
	Physical behaviours	2	5.9
	24-h activities	2	5.9
Spanish (*n* = 7)	24-h movement behaviours	4	57.1
	Movement behaviours	4	57.1
	24-h activity cycle	1	14.3
	24-h activity cycle behaviours	1	14.3
Portuguese	24-h movement behaviours	1	33.3
(*n* = 3)	Health-related behaviours	1	33.3
	Movement behaviours	1	33.3
Japanese	24-h movement behaviours	1	100.0
(*n* = 1)	Movement behaviours	1	100.0
Korean (*n* = 1)	24-h movement behaviours	1	100.0
French (*n* = 1)	No term used	1	100.0

^a^For simplicity, all categories only included studies which exclusively examined these age groups, outcomes, or health statuses

^b^Because terminology from non-English languages was translated into English during data extraction, the terms presented are English conceptual equivalents rather than literal linguistic translations

**Fig. 4:**
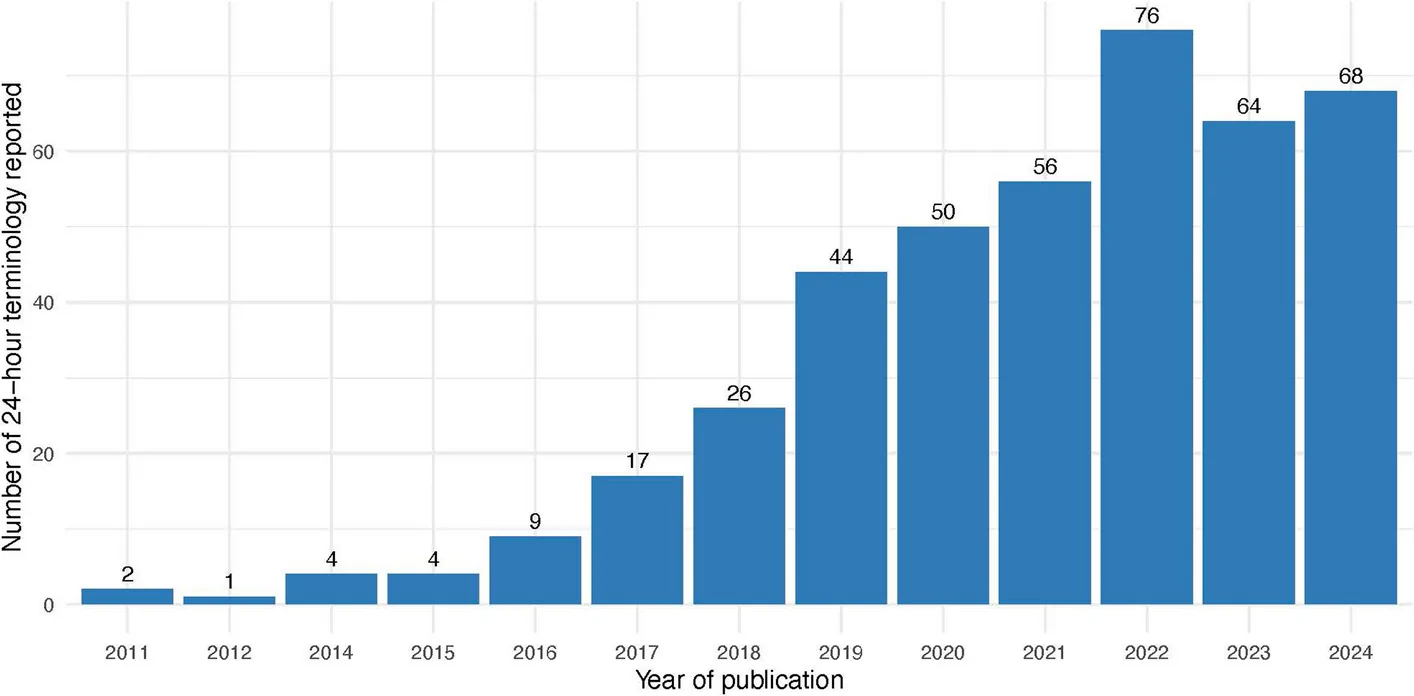
Number of terms to discuss physical activity, sedentary behaviour, and sleep throughout the 24-h cycle by year

**Fig. 5:**
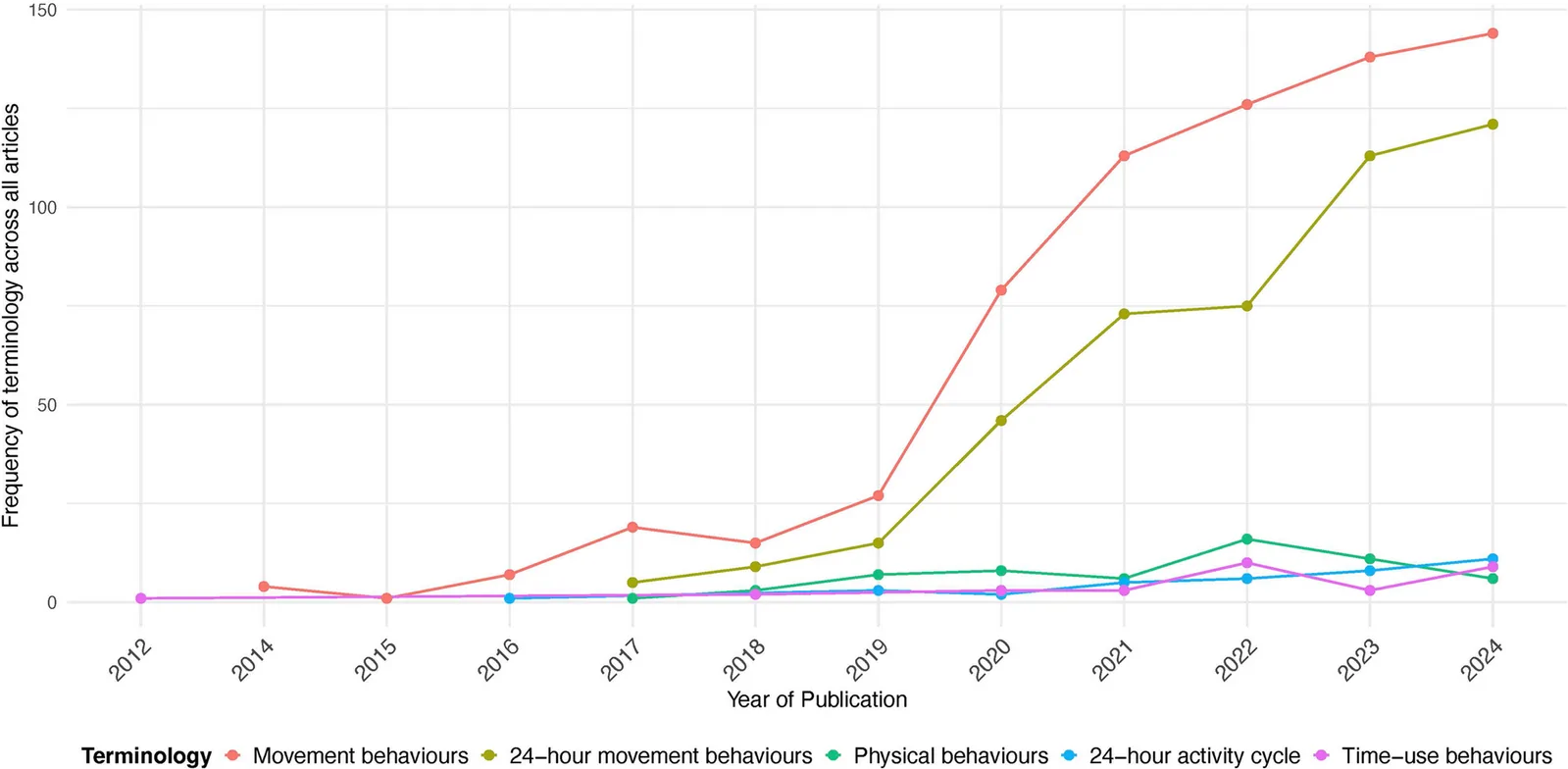
Frequency of use of the five most commonly adopted terms to discuss physical activity, sedentary behaviour, and sleep throughout the 24-h cycle by year

The number of distinct terms used to discuss physical activity, sedentary behaviour, and sleep collectively has grown steadily from 2012 to 2024 (Fig. 4). As indicated in Fig. 5, articles published from 2019 onward appear to have favoured *movement behaviours* and *24-h movement behaviours* more than other terminology. Three hundred and ninety-one used two different terms (39.9%), 118 articles used three different terms (12.0%), 49 articles used four different terms (5.0%), and 25 articles used five or more different terms (2.6%).

## Discussion

The current terminology used to collectively describe physical activity, sedentary behaviour, and sleep across the 24-h cycle lacks consistency. This study identified 199 separate terms used to describe the coalescence of these behaviours, of which *movement behaviours* and *24-h movement behaviours* were the most popular terms. Three hundred and seven articles used at least two different terms. Standing behaviour was reported as a separate component of the 24-h cycle in 65/979 articles (6.7%). These findings highlight the need for consensus on: 1) how to collectively discuss physical activity, sedentary behaviour, and sleep across the 24-h cycle; and 2) whether standing behaviour should be considered a separate component.

Physical activity, sedentary behaviour, and sleep each have established definitions and terminologies [14–16, 22]. All of the articles included in our scoping review referred to physical activity, sedentary behaviour, and sleep using these established definitions and terminologies. Physical activity was also referred to as *exercise* or *physical exercise*, and some articles adopted specific physical activity modalities such as *walking*, *running*, *cycling*, or less common terms such as *exercise-related behaviour* or *physical movement*. Importantly, using terminology such as *exercise* or *exercise-related behaviour* is inaccurate since exercise is a sub-category of physical activity, which refers to planned or structured physical activity for the purposes of increasing or maintaining physical fitness [14].

Articles that had early years children and youth as a population of interest used physical activity terms specific to that age range, including: *tummy time*, *crawling*, *outdoor playtime*, and *energetic play*. While sedentary behaviour was also referred to as *screen time* or *sedentary time*, other terms included modalities of sedentary behaviour such as *sitting time*, *lying time*, and *reading time*. Articles that included children and youth commonly used *screen time* interchangeably with *sedentary behaviour*. Notably, 131 articles (13.4%) published after 2012 adopted *physical inactivity* or *inactivity* to refer to sedentary behaviour, which disagrees with the Sedentary Behaviour Research Network’s efforts to differentiate “sedentary behaviour” and “physical inactivity” [31]. Sleep was also referred to as *sleep duration* or *sleep time*, with other terms including *bedtime* or *time in bed*, *sleep periods*, and *sleep quantity*. While we did not examine in detail why these additional terms were used to describe the constructs of physical activity, sedentary behaviour, and sleep, it is important to note that many of these terms are also sub-categories of these behaviours. Screen time is the time spent on screen-based behaviours, which can be performed while being sedentary, while sedentary time refers to the duration of sedentary behaviour [22]. Sleep duration and sleep time refer to the quantity of time spent sleeping [32]. While all studies in our review used the shared terminology of *physical activity*, *sedentary behaviour*, and *sleep* to refer to these behaviours as independent constructs, there are still clearly some inconsistencies in how terminology is being applied in order to independently described each of these behaviours.

However, when discussing these behaviours collectively, there is less consistency. A total of 199 separate terms were used for discussing the 24-h cycle of physical activity, sedentary behaviour, and sleep. Whether these terms are each semantically different is difficult to discern. For example, a recent cross-sectional study by Balbim and colleagues [33] used the terms *24-h activity cycle, 24-h activity cycle profiles*, *24-h activity cycle, activity profiles, 24-h activity cycle behaviours, 24-h activity cycle time-use behaviours, 24-h activity cycle composition, 24-h activity cycle behaviour compositions,* and *24-h activity cycle patterns*. Each of these terms is clearly using the same lexicon, but varies slightly from term-to-term. Some of these differences might reflect analytical contexts rather than inconsistency; Balbim and colleagues [33] used a combination of compositional data analysis and latent profile analysis to examine their research question, which might account for the variability in terms used (e.g., *24-h activity cycle profiles* or *24-h activity cycle composition)*. Nonetheless, this example still highlights the lack of unified terminology in the field. Balbim and colleagues [33] used 8 different terms for collectively discussing physical activity, sedentary behaviour, and sleep. The *24-h activity cycle* is described as a *profile*, *composition*, *pattern*, *behaviours*, and *time-use behaviours*. Whether these choices in wording reflect genuine semantic differences is difficult to discern. However, at the very least, there is clearly a lack of precision and consistency in the language used surrounding physical activity, sedentary behaviour, and sleep.

Importantly, it does seem that certain terminology is being used more often in recent years, particularly *movement behaviours* and *24-h movement behaviours*. The increased popularity of these terms likely corresponds with the development and dissemination of the Canadian 24-h Movement Guidelines [9, 12], which used these terms to collectively discuss physical activity, sedentary behaviour, and sleep. However, the popularity of this terminology is likely influenced by who is conducting research in this area. Physical activity researchers – many of whom have a background in exercise physiology, kinesiology, or physical education – might be more focused on ‘movement’ and thus find terminology such as *movement behaviours* to be more enticing. Behavioural scientists, on the other hand, may prefer a more neutral term such as *24-h activity behaviours*. While it is difficult within the context of this review to identify with certainty the terminology preferences of researchers, it is clear that there is still a plurality of terminology being used in the scientific literature.

Whether standing behaviour is considered a separate behaviour of the 24-h cycle of physical activity, sedentary behaviour, and sleep is also unclear. Standing behaviour was mentioned in 98 articles, of which 65 (6.7%) treated it as a separate component of the 24-h day, in addition to physical activity, sedentary behaviour, and sleep. Our findings suggest that most studies do not conceptualize standing behaviour as a separate interdependent construct of the 24-h cycle of physical activity, sedentary behaviour, and sleep. However, if sitting and standing do indeed share distinct impacts on health [13], then clarity is needed on how standing behaviour integrates into the 24-h cycle of physical activity, sedentary behaviour, and sleep.

Should there be only one term for discussing this 24-h cycle of behaviour? Using a single term may seem reductive, as this biological phenomenon can be classified behaviourally (e.g., physical activity, sedentary behaviour, standing behaviour, sleep), posturally (e.g., lying, sitting, standing, moving), and energetically (i.e., METs) [19]. Sub-classifications in any `24-h` taxonomy also require clarification. For instance, some have used the term *movement behaviours* to refer to physical activity and sedentary behaviour [34], while others have used it to describe physical activity, sedentary behaviour, and sleep [7]. As noted in this scoping review, the most popular terms for collectively describing physical activity, sedentary behaviour, and sleep are *movement behaviours* and *24-h movement behaviours* – likely because current guidelines use these terms [9, 12]. And yet, sleep is defined by its lack of movement [16]. It is beyond the scope of this review to untangle this Gordian Knot of semantics; however, these issues clearly highlight the need for a consensus on terminology in this field.

Terminology was also inconsistent regardless of the publication language. For example, among the 34 articles written in Chinese, there were 14 different terms used for collectively discussing physical activity, sedentary behaviour, and sleep. The seven articles written in Spanish used four different terms, and among the three Portuguese articles, three separate terms were used. Thus, in both English language and non-English language studies, there was a lack of consistency in how physical activity, sedentary behaviour, and sleep were collectively discussed.

The terminology used for collectively discussing physical activity, sedentary behaviour, and sleep also lacks consistency across different populations and outcomes of interest. For instance, the most popular term used to describe the 24-h cycle for each outcome domain (i.e., physical, psychosocial, or behavioural) was *movement behaviours*; 62.8% of studies exclusively examining physical outcomes used the term *movement behaviours*, while 75.1% of studies exclusively investigating psychosocial outcomes and 81.8% of behavioural outcomes used this term. Similarly, *movement behaviours* was the most popular term for describing the 24-h cycle across all age groups, but the frequency at which this term was used varied between 44.4% (older adults) and 78.1% (children).

Across different continents, *movement behaviours* and *24-h movement behaviours* were the most popular terms for each continent. However, the frequency of use varied by continent. For example, *movement behaviours* was used in 31.9% of articles from Europe, while this term was used in 63.7% of articles from North America. The term *24-h movement behaviours* was used in 23.8% of articles from Europe, but was used in 46.4% of articles from South America. Thus, while these terms appear to be the most popular terminology across the globe, the popularity of these terms in the scientific vernacular is dependent upon location.

The importance of developing a well-recognized and common terminology is evident given how rapidly this field is expanding. Moreover, to ensure clarity, enhance reproducibility, and enable the translation of future research into coherent guidelines, policies, and practices, a consensus on terminology is needed [1]. From 2011 to 2019, there were 140 articles (14.30%) published which examined physical activity, sedentary behaviour, and sleep collectively; 839 articles (85.70%) have been published since 2020. The number of articles published in 2024 (*n* = 198 [20.22%])) was nearly double the number published in 2020 (*n* = 113 [11.54%]). Empirical articles were conducted in 99 countries/regions, and the research teams were composed of researchers from 93 countries/regions. However, the terminology used to discuss these behaviours is still far from consistent. The number of distinct terms used to discuss these behaviours mimic the growth of articles published until 2022, with a decrease in 2023, followed by another increase in 2024. For example, there were 68 different terms used to collectively describe physical activity, sedentary behaviour, and sleep in 2024.

The current issues which we highlight in this study will exacerbate if not addressed soon. Having too many terms in the literature will only distract from the message that these behaviours have an important and interactive effect on health and well-being. As we noted in our editorial from 2021 [25], “our explanations for the collective impacts of physical activity, sedentary behaviour, and sleep on health will crumble like the Tower of Babel if the language we use is not uniform.” It is critical that this area of research now establish how we will collectively refer to these behaviours. Future work is needed to determine whether any agreed upon terminology should be adapted for use with the general public or specific interest groups.

### Strengths and limitations

There are several strengths to this study. This was a review of more than 900 articles examining the 24-h cycle of physical activity, sedentary behaviour, and sleep. Our study examined the literature across multiple languages, health outcomes, and age groups. We therefore think that our results are broadly generalizable and represent the current issues within the field of’24-h’ research.

Several limitations are worth noting. This study focused on the overall body of literature examining the 24-h cycle of physical activity, sedentary behaviour, and sleep. We did not explore whether individual research disciplines use a specific set of terminology compared with other disciplines (e.g., psychology versus physiology). Our last search was conducted on September 20th 2024. Gray literature was not included, which may have limited the different types of terminology used. Of the 979 articles included in this review, 99 were from the INTUE publication list and hence it is plausible that our search strategy may have missed other papers. This study only included articles published in 7 languages. Terminology was translated into English during data extraction by reviewers fluent in both languages. Although this approach enabled the inclusion of literature in multiple languages and facilitated synthesis, it is possible that subtle linguistic nuances present in the original wording may not be fully captured in translation. Consequently, some terms may represent the closest conceptual equivalent in English rather than a direct linguistic match. Each term was treated as distinct; however, some terms (e.g., *physical behaviours* and *24-h physical behaviours*) may be considered semantically identical. Study quality was not assessed. In the event that multiple papers were published by the same authorship group using the same cohort, each paper was examined independently.

Notably, we did not examine whether the productivity of specific research teams influenced how terminology has been used in the literature. It is plausible that raw publication counts for different terms may be misleading, and reflect the productivity of specific research teams rather than widespread adoption of terminology. Future examinations should use a bibliometric analysis of the authors and research teams in this field.

## Conclusions

The terminology used to describe the individual behaviours of physical activity, sedentary behaviour, and sleep are fairly consistent in research. However, the terminology used to describe physical activity, sedentary behaviour, and sleep in an integrated manner has been far more variable with the two most popular terms being *movement behaviours* and *24-h movement behaviours*. While these terms are the most pervasive, there is no agreement about whether sleep, which is defined by its lack of movement [16], can be classified as a “movement behaviour”. It is also unclear whether or not standing behaviour is a separate component of this 24-h cycle, although most studies do not conceptualize standing behaviour as a separate interdependent construct. In order to advance future research, guidelines, policies, and practices related to the 24-h cycle of physical activity, sedentary behaviour, and sleep, the field must establish a standardized and universally accepted set of terms and definitions for discussing these behaviours collectively. Future work is still needed to determine what terminology should be used when engaging with the general public.

## Supplementary Information

Supplementary Material 1. Search Strategy.

Supplementary Material 2. Bibliography of included articles in the scoping review.

Supplementary Material 3. Frequencies of study designs, country where research was conducted, health status of sample, and outcomes measured.

Supplementary Material 4. Absolute and relative frequencies of terminology used for discussing physical activity, sedentary behaviour, and sleep as individual behaviours.

Supplementary Material 5. Frequency of terms adopted to discuss sleep, sedentary behaviour, and physical activity collectively.

## Data Availability

The review database (coding sheets, and R scripts) is openly available at the Open Science Framework: [https://osf.io/s8anf/overview] (https:/osf.io/s8anf/overview).

## Abbreviations

INTUE: International Network of Time-Use Epidemiologists
JBI: Joanna Briggs Institute
METs: Metabolic equivalents
PRISMA-ScR: Preferred Reporting Items for Systematic Reviews and Meta-Analyses extension for Scoping Reviews
