# Supplementary material for: Terminology for describing the 24-h cycle of physical activity, sedentary behaviour, and sleep: a scoping review
Source: Int J Behav Nutr Phys Act. 2026 Aug 5;23:107. doi: 10.1186/s12966-026-01945-6 (PMC13729492; doi:10.1186/s12966-026-01945-6)
Supplement: Supplementary file 1 [file 12966_2026_1945_MOESM1_ESM.docx]

**Supplementary Material S1.** Search strategy

**Search Strategy Terms**

| **Sedentary** | **Sleep** | **Physical Activity** | **Health behaviours of physical activity, sedentary behaviour, and sleep** |
| --- | --- | --- | --- |
| Sedentar* | Sleep* | Exercise | “24-hour activity cycle” |
| Inactiv* | Napping | Sports | “24-hour activity” |
| Sitting | Naps | Physical* | “24-hour movement behavior*” |
| Seated | Circadian* | Training | “24-hour movement*” |
| “Screen time” | “Bed time” | Fitness | “24-hour time use” |
| Stationar* | “Wake time” | MVPA | “24-hour time use behavior*” |
|  | Nocturnal | (light* or moderate or moderate-to-vigorous or aerobic or strength* or weight* or resistance physical*) adj3 (training* or activit* or movement)) | “24-hour day” |
|  | Diurnal |  | “24-hour day behavior*” |
|  | Onset |  | “movement behavior*” |
|  | Offset |  | “physical behavior*” |
|  | “Rise time” |  | “Time use” |
|  |  |  | Time-use |
|  | Nighttime |  | ("24-hour" or 24h or 24hr or "twenty-four hour") adj3 (cycle or "activity cycle day" or movement* or "time-use*" or behavior*) |

**Medline (Limits: Journal article or Meta-analysis or "Systematic review"). Comment:**

1. (Sleep* or Naps or Napping or Circadian* or "Bed time" or bedtime or "wake time" or nocturnal or diurnal or Onset or Offset or "Rise time" or nighttime).mp. [mp=title, book title, abstract, original title, name of substance word, subject heading word, floating sub-heading word, keyword heading word, organism supplementary concept word, protocol supplementary concept word, rare disease supplementary concept word, unique identifier, synonyms, population supplementary concept word, anatomy supplementary concept word]

2. exp Sleep/

3. 1 or 2

4. (Sedentar* or Inactiv* or Sitting or Seated or Stationary).mp. [mp=title, book title, abstract, original title, name of substance word, subject heading word, floating sub-heading word, keyword heading word, organism supplementary concept word, protocol supplementary concept word, rare disease supplementary concept word, unique identifier, synonyms, population supplementary concept word, anatomy supplementary concept word]

5. Sedentary Behavior/ or Sitting Position/

6. 4 or 5

7. (exercise* or sport* or training or fitness or mvpa).mp. [mp=title, book title, abstract, original title, name of substance word, subject heading word, floating sub-heading word, keyword heading word, organism supplementary concept word, protocol supplementary concept word, rare disease supplementary concept word, unique identifier, synonyms, population supplementary concept word, anatomy supplementary concept word]

8. ((light* or moderate or moderate-to-vigorous or aerobic or strength* or resistance or weight* or physical*) adj3 (training* or activit* or movement)).mp. [mp=title, book title, abstract, original title, name of substance word, subject heading word, floating sub-heading word, keyword heading word, organism supplementary concept word, protocol supplementary concept word, rare disease supplementary concept word, unique identifier, synonyms, population supplementary concept word, anatomy supplementary concept word]

9. exp Exercise/ or exp Sports/ or Physical Fitness/ or Physical Exertion/

10. 7 or 8 or 9

11. 3 and 6 and 10

12. (("24-hour activity cycle" or "24-hour activity" or "24-hour movement behavior*" or "24-hour movement*" or "24-hour time use" or "24-hour time use behavior*" or "24-hour day" or "24-hour day behavior*" or "movement behavior*" or "physical behavior*" or time-use or "time use")).mp. [mp=title, book title, abstract, original title, name of substance word, subject heading word, floating sub-heading word, keyword heading word, organism supplementary concept word, protocol supplementary concept word, rare disease supplementary concept word, unique identifier, synonyms, population supplementary concept word, anatomy supplementary concept word]

13. (("24-hour" or 24h or 24hr or "twenty-four hour") adj3 (cycle or "activity cycle day" or movement* or "time-use*" or behavior*)).mp. [mp=title, book title, abstract, original title, name of substance word, subject heading word, floating sub-heading word, keyword heading word, organism supplementary concept word, protocol supplementary concept word, rare disease supplementary concept word, unique identifier, synonyms, population supplementary concept word, anatomy supplementary concept word]

14. exp Circadian Rhythm/

15. 12 or 13 or 14

16. 11 and 15

17. limit 16 to (journal article or meta analysis or "systematic review")

**EMBASE (Limits: Publication Source: Journals or Publication Types: Articles or Articles in Press)**

1. (Sleep* or Naps or Napping or Circadian* or "Bed time" or bedtime or "wake time" or nocturnal or diurnal or Onset or Offset or "Rise time" or nighttime).mp. [mp=title, abstract, heading word, drug trade name, original title, device manufacturer, drug manufacturer, device trade name, keyword heading word, floating subheading word, candidate term word]

2. exp Sleep/

3. 1 or 2

4. (Sedentar* or Inactiv* or Sitting or Seated or Stationary).mp. [mp=title, abstract, heading word, drug trade name, original title, device manufacturer, drug manufacturer, device trade name, keyword heading word, floating subheading word, candidate term word]

5. sedentary lifestyle/ or sitting/ or physical inactivity/

6. 4 or 5

7. (exercise* or sport* or training or fitness or mvpa).mp. [mp=title, abstract, heading word, drug trade name, original title, device manufacturer, drug manufacturer, device trade name, keyword heading word, floating subheading word, candidate term word]

8. ((light* or moderate or moderate-to-vigorous or aerobic or strength* or resistance or weight* or physical*) adj3 (training* or activit* or movement)).mp. [mp=title, abstract, heading word, drug trade name, original title, device manufacturer, drug manufacturer, device trade name, keyword heading word, floating subheading word, candidate term word]

9. exp exercise/ or exp physical activity/ or sport/

10. 7 or 8 or 9

11. 3 and 6 and 10

12. ("24-hour activity cycle" or "24-hour activity" or "24-hour movement behavior*" or "24-hour movement*" or "24-hour time use" or "24-hour time use behavior*" or "24-hour day" or "24-hour day behavior*" or "movement behavior*" or "physical behavior*" or time-use or "time use").mp. [mp=title, abstract, heading word, drug trade name, original title, device manufacturer, drug manufacturer, device trade name, keyword heading word, floating subheading word, candidate term word]

13. (("24-hour" or 24h or 24hr or "twenty-four hour") adj3 (cycle or "activity cycle day" or movement* or "time-use*" or behavior*)).mp. [mp=title, abstract, heading word, drug trade name, original title, device manufacturer, drug manufacturer, device trade name, keyword heading word, floating subheading word, candidate term word]

14. day length/ or circadian rhythm/

15. 12 or 13 or 14

16. 11 and 15

17. limit 16 to ((article or article in press) and journal)

**CINAHL (Limits: Peer Reviewed; Research Article). Limiters: Source Types (Academic Journals)**

S16	S11 AND S15

S15	S12 OR S13 OR S14

S14	(MH "Circadian Rhythm+")

S13	((“24-hour" or 24h or 24hr or "twenty-four hour") N3 (rhythm* OR cycle* OR day* OR movement* OR “time-us*” or timeus* OR behavior* or behaviour*))

S12	"24-hour activity cycle" OR "24-hour activity" OR "24-hour movement behavior*" OR "24-hour movement*" OR "24-hour time use" OR "24-hour time use behavior*" OR "24-hour day" OR "24-hour day behavior*" OR "movement behavior*" OR "physical behavior*" OR time-use OR "time use"

S11	S3 AND S6 AND S10

S10	S7 OR S8 or S9

S9	(MH "Exercise+") OR (MH "Physical Activity") OR (MH "Sports+")

S8	((light* OR moderate OR moderate-to-vigorous OR aerobic OR strength* OR resistance OR weight* OR physical*) N3 (training* OR activit* OR movement))

S7	exercise* OR sport* OR training OR fitness OR mvpa

S6	S4 OR S5

S5	(MH "Life Style, Sedentary+")

S4	sedentar* OR inactiv* OR sitting OR seated OR stationary

S3	S1 OR S2

S2	(MH "Sleep+") OR (MH "Sleep Duration") OR (MH "Sleep Latency") OR (MH "Sleep Hygiene+") OR (MH "Sleep Stages+") OR (MH "Sleep Quality") OR (MH "Sleep, REM") OR (MH "Deep Sleep") OR (MH "Sleep Deprivation")

S1	sleep* OR naps OR Napping OR Circadian* OR "bed time" OR bedtime OR "wake time" OR nocturnal OR diurnal OR onset or offset OR "rise time" OR nighttime

**PsycInfo (Limits: Scholarly (Peer Reviewed) Journals; Publication Type: Peer Reviewed Journal; Exclude Dissertations).**

S16	S11 AND S15

S15	S12 OR S13 OR S14

S14	DE "Human Biological Rhythms" OR DE "Chronotype" OR DE "Cortisol Awakening Response" OR DE "Sleep Wake Cycle"

S13	((“24-hour" or 24h or 24hr or "twenty-four hour") N3 (rhythm* OR cycle* OR day* OR movement* OR “time-us*” or timeus* OR behavior* or behaviour*))

S12	"24-hour activity cycle" OR "24-hour activity" OR "24-hour movement behavior*" OR "24-hour movement*" OR "24-hour time use" OR "24-hour time use behavior*" OR "24-hour day" OR "24-hour day behavior*" OR "movement behavior*" OR "physical behavior*" OR time-use OR "time use"

S11	S3 AND S6 AND S10

S10	S7 OR S8 OR S9

S9	DE "Physical Activity" OR DE "Exercise" OR DE "Physical Fitness"

S8	((light* OR moderate OR moderate-to-vigorous OR aerobic OR strength* OR resistance OR weight* OR physical*) N3 (training* OR activit* OR movement))

S7	exercise* OR sport* OR training OR fitness OR mvpa

S6	S4 OR S5

S5	DE "Sedentary Behavior"

S4	sedentar* OR inactiv* OR sitting OR seated OR stationary

S3	S1 OR S2

S2	DE Sleep

S1	sleep* OR naps OR napping OR circadian* OR "bed time" OR bedtime OR "wake time" OR nocturnal OR diurnal OR onset OR offset OR "rise time" OR nighttime

**Web of Science 2024-09-18 (Limits: Article or Review Article)**

1. ALL=(sleep* OR naps OR napping OR circadian* OR "bed time" OR bedtime OR "wake time" OR nocturnal OR diurnal OR onset OR offset OR "rise time" OR nighttime)

2. ALL=(sedentar* OR inactiv* OR sitting OR seated OR stationary)

3. ALL=(exercise* OR sport* OR training OR fitness OR mvpa

4. TS=((light* OR moderate OR moderate-to-vigorous OR aerobic OR strength* OR resistance OR weight* OR physical*) Near/3 (training* OR activit* OR movement))

5. #3 OR #4

6. #1 AND #2 AND #5

7. ALL=("24-hour activity cycle" OR "24-hour activity" OR "24-hour movement behavior*" OR "24-hour movement*" OR "24-hour time use" OR "24-hour time use behavior*" OR "24-hour day" OR "24-hour day behavior*" OR "movement behavior*" OR "physical behavior*" OR time-use OR "time use")

8. TS=((("24-hour" OR 24h OR 24hr OR "twenty-four hour") Near/3 (rhythm* OR cycle* OR day* OR movement* OR “time-us*” or timeus* OR behavior* or behaviour*)))

9. #7 OR #8

10. #6 AND #9

11. #10 AND (DT==("ARTICLE" OR "REVIEW" OR "EARLY ACCESS"))

**Scopus 2024-09-18 (Limits: Article or Review)**

( ALL ( sedentar* OR inactiv* OR sitt* OR stationary ) ) AND ( ALL ( sleep* OR nap* OR circadian* OR "bed time" OR bedtime OR "wake time" OR nocturnal OR diurnal OR onset OR offset OR "rise time" OR nighttime ) ) AND ( ( ALL ( exercise* OR sport* OR training OR fitness OR mvpa ) ) OR ( ALL ( ( ( light* OR moderate OR moderate-to-vigorous OR aerobic OR strength* OR resistanceOR weight* OR physical* ) W/3 ( training* OR activit* OR movement ) ) ) ) ) AND ( ALL ( "24-hour activity cycle" OR "24-hour activity" OR "24-hour movement behavior*" OR "24-hour movement*" OR "24-hour time use" OR "24-hour time use behavior*" OR "24-hour day" OR "24-hour day behavior*" OR "movement behavior*" OR "physical behavior*" OR time-use OR "time use" ) ) OR ( ( ALL ( "24-hour" OR 24h OR twenty-four W/3 cycle OR day ) ) OR ( ALL ( "24-hour" OR 24h OR "twenty-four hour" W/3 movement* ) ) OR ( ALL ( "24-hour" OR 24h OR "twenty-four hour" W/3 time-use* ) ) OR ( ALL ( "24-hour" OR 24h OR "twenty-four hour" W/3 behavior ) ) ) AND ORIG-LOAD-DATE > 20240814 AND ( LIMIT-TO ( DOCTYPE , "ar" ) OR LIMIT-TO ( DOCTYPE , "re" ) )

**ERIC (Limits: Scholarly (Peer Reviewed) Journals)**

S14	S9 AND S13

S13	S10 OR S11 OR S12

S12	DE "Time Factors (Learning)" OR DE "Scheduling" OR DE "School Schedules" OR DE "Working Hours"

S11	((“24-hour" or 24h or 24hr or "twenty-four hour") N3 (rhythm* OR cycle* OR day* OR movement* OR “time-us*” or timeus* OR behavior* or behaviour*))

S10	"24-hour activity cycle" OR "24-hour activity" OR "24-hour movement behavior*" OR "24-hour movement*" OR "24-hour time use" OR "24-hour time use behavior*" OR "24-hour day" OR "24-hour day behavior*" OR "movement behavior*" OR "physical behavior*" OR time-use OR "time use"

S9	S3 AND S4 AND S8

S8	S5 OR S6 OR S7

S7	DE "Physical Activity Level" OR DE "Physical Activities" OR DE "Athletics" OR DE "Dance" OR DE "Exercise"

S6	((light* OR moderate OR moderate-to-vigorous OR aerobic OR strength* OR resistance OR weight* OR physical*) N3 (training* OR activit* OR movement))

S5	exercise* OR sport* OR training OR fitness OR mvpa

S4	sedentar* OR inactiv* OR sitting OR seated OR stationary

S3	S1 OR S2

S2	DE "Sleep"

S1	sleep* OR naps OR napping OR circadian* OR "bed time" OR bedtime OR "wake time" OR nocturnal OR diurnal OR onset OR offset OR "rise time" OR nighttime

**Education Source (Limits: Peer Reviewed)**

S11	S6 AND S10

S10	S7 OR S8 OR S9

S9	DE "Time Factors (Learning)" OR DE "Scheduling" OR DE "School Schedules" OR DE "Working Hours"

S8	((“24-hour" or 24h or 24hr or "twenty-four hour") N3 (rhythm* OR cycle* OR day* OR movement* OR “time-us*” or timeus* OR behavior* or behaviour*))

S7	"24-hour activity cycle" OR "24-hour activity" OR "24-hour movement behavior*" OR "24-hour movement*" OR "24-hour time use" OR "24-hour time use behavior*" OR "24-hour day" OR "24-hour day behavior*" OR "movement behavior*" OR "physical behavior*" OR time-use OR "time use"

S6	S1 AND S2 AND S5

S5	S3 OR S4

S4	((light* OR moderate OR moderate-to-vigorous OR aerobic OR strength* OR resistance OR weight* OR physical*) N3 (training* OR activit* OR movement))

S3	exercise* OR sport* OR training OR fitness OR mvpa

S2	sedentar* OR inactiv* OR sitting OR seated OR stationary

S1	sleep* OR naps OR napping OR circadian* OR "bed time" OR bedtime OR "wake time" OR nocturnal OR diurnal OR onset OR offset OR "rise time" OR nighttime

**Compendex 2024-08-09 (Limits: Journal Article, Preprint, Article in Press)**

(((( (((Sedentar* OR Inactiv* OR Sitt* OR Stationary) WN ALL))) AND ( (((Sleep* OR Nap* OR Circadian* OR {Bed time} OR bedtime OR {wake time} OR nocturnal OR diurnal OR Onset OR Offset OR {Rise time} OR nighttime) WN ALL))) AND ( ((((exercise* OR sport* OR training OR fitness OR mvpa) WN ALL)) OR ((light* OR moderate OR {moderate-to-vigorous} OR aerobic OR strength* OR resistance OR weight* OR physical* {Near/3} training* OR activit* OR movement) WN ALL))) AND ( (((({24-hour} OR 24h OR {twenty-four hour} {Near/3} cycle OR day) WN ALL)) AND ((rhythm* OR cycle* OR day* OR movement* OR {time use} OR timeus* OR behavior* OR behaviour*) WN ALL))))) AND (({ja} OR {pp} OR {ip}) WN DT))
