# Supplementary material for: Terminology for describing the 24-h cycle of physical activity, sedentary behaviour, and sleep: a scoping review
Source: Int J Behav Nutr Phys Act. 2026 Aug 5;23:107. doi: 10.1186/s12966-026-01945-6 (PMC13729492; doi:10.1186/s12966-026-01945-6)
Supplement: Supplementary file 2 [file 12966_2026_1945_MOESM2_ESM.docx]

**Supplementary Material S2**. Bibliography of Included Articles in the Scoping Review

1.	Aadahl M, Andreasen AH, Petersen CB, Gupta N, Holtermann A, Lau CJ. Should leisure‐time sedentary behavior be replaced with sleep or physical activity for prevention of diabetes? Scandinavian Journal of Medicine & Science in Sports. 2021;31(5):1105-14.

2.	Abd Rahim MH, Ibrahim MI, Ab Rahman A, Yaacob NM. Translation, Cross-Cultural Adaptation and Validation of Movement Behaviour Questionnaire into Malay Language (MBQ-M) for Measuring Movement Behaviors among Preschool Children in Kelantan, Malaysia. Healthcare (2227-9032). 2023;11(9):1276.

3.	Abdeta C, Cliff D, Kariippanon K, Deksisa A, Garoma S, Tesfaye D, et al. Adherence to the WHO physical activity, screen time and sleep guidelines and associations with socio-demographic factors among Ethiopian preschool children: The SUNRISE study. Journal of Activity, Sedentary and Sleep Behaviors. 2024;3(1):22-.

4.	Adebowale TO, Taylor BJ, Gray AR, Galland BC, Heath A-LM, Fortune S, et al. Long-Term Follow-Up of a Randomized Controlled Trial to Reduce Excessive Weight Gain in Infancy: Protocol for the Prevention of Overweight in Infancy (POI) Follow-Up Study at 11 Years. JMIR research protocols. 2020;9(11):e24968.

5.	Aguilar-Farias N, Martino-Fuentealba P, Chandia-Poblete D. Correlates of device-measured physical activity, sedentary behaviour and sleeping in children aged 9-11 years from Chile: ESPACIOS study. Retos. 2020;37:1-10.

6.	Aguilar-Farias N, Toledo-Vargas M, Miranda-Marquez S, Cortinez-O'Ryan A, Cristi-Montero C, Rodriguez-Rodriguez F, et al. Sociodemographic Predictors of Changes in Physical Activity, Screen Time, and Sleep among Toddlers and Preschoolers in Chile during the COVID-19 Pandemic. International journal of environmental research and public health. 2020;18(1).

7.	Alanazi YA, Parrish AM, Okely AD. Impact of the COVID-19 virus outbreak on 24-h movement behaviours among children in Saudi Arabia: A cross-sectional survey. Child: Care, Health and Development. 2022;48(6):1031-9.

8.	Alanazi YA, Sousa-Sa E, Chong KH, Parrish A-M, Okely AD. Systematic Review of the Relationships between 24-Hour Movement Behaviours and Health Indicators in School-Aged Children from Arab-Speaking Countries. International journal of environmental research and public health. 2021;18(16).

9.	Alaqil AI, del Pozo Cruz B, Alothman SA, Ahmadi MN, Caserotti P, Al-Hazzaa HM, et al. Feasibility and acceptability of a cohort study baseline data collection of device-measured physical behaviors and cardiometabolic health in Saudi Arabia: expanding the Prospective Physical Activity, Sitting and Sleep consortium (ProPASS) in the Middle Ea. BMC Public Health. 2024;24(1):1-15.

10.	Alfawaz R, Aljuraiban G, Almarzooqi M, Alghannam A, Bahammam A, Dobia A, et al. The recommended amount of physical activity, sedentary behavior, and sleep duration for healthy Saudis: A joint consensus statement of the Saudi Public Health Authority. Annals of Thoracic Medicine. 2021;16(3):239-44.

11.	Alkhraiji MH, Barker AR, Williams CA. Reliability and validity of using the global school-based student health survey to assess 24 hour movement behaviours in adolescents from Saudi Arabia. Journal of Sports Sciences. 2022;40(14):1578-86.

12.	Alonso-Martinez AM, Legarra-Gorgonon G, Garcia-Alonso Y, Ramirez-Velez R, Alonso-Martinez L, Erice-Echegaray B, et al. Gamified family-based health exercise intervention to improve adherence to 24-h movement behaviors recommendations in children: "3, 2, 1 Move on Study". Trials. 2023;24(1):531.

13.	Amornsriwatanakul A, Jalayondeja C, Wattanapisit A, Chootong R, Saovieng S, Nantabancha A, et al. Thai Preschoolers' movement behaviors outside kindergarten: prevalence of meeting individual and integrated movement guidelines. Pediatric Research. 2024;95(5):1363-71.

14.	Andrianasolo R, Menai M, Galan P, Hercberg S, Oppert J-M, Kesse-Guyot E, et al. Leisure-Time Physical Activity and Sedentary Behavior and Their Cross-Sectional Associations with Excessive Daytime Sleepiness in the French SU.VI.MAX-2 Study. International Journal of Behavioral Medicine. 2016;23(2):143-52.

15.	Antczak D, Lonsdale C, del Pozo Cruz B, Parker P, Sanders T. Reliability of GENEActiv accelerometers to estimate sleep, physical activity, and sedentary time in children. The International Journal of Behavioral Nutrition and Physical Activity. 2021;18.

16.	Antczak D, Sanders T, Del Pozo Cruz B, Parker P, Lonsdale C. Day-to-day and longer-term longitudinal associations between physical activity, sedentary behavior, and sleep in children. Sleep. 2021;44(4).

17.	Aoki A, Togoobaatar G, Tseveenjav A, Nyam N, Zuunnast K, Lkhagvasuren G, et al. Socioeconomic and lifestyle factors associated with mental health problems among Mongolian elementary school children. Social Psychiatry and Psychiatric Epidemiology. 2022;57(4):791-803.

18.	Arbour-Nicitopoulos KP, Bassett-Gunter RL, Leo J, Sharma R, Olds T, Latimer-Cheung AE, et al. A cross-sectional examination of the 24-hour movement behaviours in Canadian youth with physical and sensory disabilities. Disability and Health Journal. 2021;14(1):100980.

19.	Arbour-Nicitopoulos KP, James ME, Moore SA, Sharma R, Martin Ginis KA. Movement behaviors and health of children and youth with disabilities: Impact of the 2020 COVID-19 pandemic. Paediatrics and Child Health (Canada). 2022;27:S151-S7.

20.	Argumedo G, Taylor J, Brunel JO, Gaytán-González A, González-Casanova I, Villalobos MFG, et al. Results from the 2022 Mexican report card on physical activity for children and adolescents. FRONTIERS IN PUBLIC HEALTH. 2024;11.

21.	Armstrong B, Beets MW, Starrett A, Brazendale K, Turner-McGrievy G, Saelens BE, et al. Dynamics of sleep, sedentary behavior, and moderate-to-vigorous physical activity on school versus nonschool days. Sleep. 2021;44(2).

22.	Armstrong B, Covington LB, Hager ER, Black MM. Objective sleep and physical activity using 24-hour ankle-worn accelerometry among toddlers from low-income families. Sleep Health. 2019;5(5):459-65.

23.	Arnup JL, Black N, Johnston DW. Changes in children’s time use during periods of financial hardship. Journal of Population Economics. 2022;35(3):1133-62.

24.	Arts J, Chinapaw MJM, Gubbels JS, Verhoeff AP, Brons A, Veldman S, et al. Development and content validity of an application to assess 24-hour movement behaviors in 0–4-year-old children involving end-users and key stakeholders: the My Little Moves app. International Journal of Behavioral Nutrition & Physical Activity. 2024;21(1):1-16.

25.	Arts J, Gubbels JS, Verhoeff AP, Chinapaw MJM, Lettink A, Altenburg TM. A systematic review of proxy-report questionnaires assessing physical activity, sedentary behavior and/or sleep in young children (aged 0–5 years). International Journal of Behavioral Nutrition & Physical Activity. 2022;19(1):1-39.

26.	Arufe-GirÁldez VÍ, Sanmiguel-Rodríguez A, Zagalaz-SÁnchez ML, CachÓn-Zagalaz J, GonzÁlez-Valero G. Sleep, physical activity and screens in 0-4 years Spanish children during the COVID-19 pandemic: Were the WHO recommendations met? Journal of Human Sport and Exercise. 2020;17(3):1-20.

27.	Asano Y, Nagata K, Shibuya K, Fujii Y, Kitano N, Okura T. Association of 24-h movement behaviors with phase angle in community-dwelling older adults: a compositional data analysis. Aging Clinical & Experimental Research. 2023;35(7):1469-76.

28.	Atkinson HF, Norris A. A health behaviour pandemic: The COVID-19 pandemic has impacted the physical activity, sleep, and sedentary behaviour of already-struggling Canadians. Heliyon. 2023;9(8):e19005.

29.	Atoui S, Bernard P, Carli F, Liberman AS. Association between physical activity, sedentary behaviors, and sleep-related outcomes among cancer survivors: A cross-sectional study. International Journal of Behavioral Medicine. 2023.

30.	Badon SE, Ferrara A, Gabriel KP, Avalos LA, Hedderson MM. Changes in 24-Hour Movement Behaviors From Early to Late Pregnancy in Individuals With Prepregnancy Overweight or Obesity. Journal of physical activity & health. 2022;19(12):842-6.

31.	Badon SE, Ferrara A, Gabriel KP, Dooley EE, Quesenberry CP, Avalos LA, et al. Behavior reallocations within 24-h movement profiles and change in cardiometabolic biomarkers during pregnancy. Obesity. 2024;32(3):623-32.

32.	Bahammam A, Alghannam A, Aljaloud K, Aljuraiban G, Almarzooqi M, Dobia A, et al. Joint consensus statement of the Saudi Public Health Authority on the recommended amount of physical activity, sedentary behavior, and sleep duration for healthy Saudis: Background, methodology, and discussion. Annals of Thoracic Medicine. 2021;16(3):225-38.

33.	Bahl JS, Millar SC, Fraysse F, Arnold JB, Taylor M, Callary S, et al. Changes in 24-Hour Physical Activity Patterns and Walking Gait Biomechanics After Primary Total Hip Arthroplasty: A 2-Year Follow-up Study. Journal of Bone & Joint Surgery, American Volume. 2021;103(13):1166-74.

34.	Bailey CP, DiPietro L, Hayman LL, Ahmad Z, Napolitano MA. Meeting Specific 24-Hour Movement Guidelines Is Associated With BMI Among University Students With Overweight/Obesity. American Journal of Lifestyle Medicine. 2022.

35.	Baillot A, Chaput JP, Prince SA, Romain AJ, Colley RC, Lang JJ. Health associations with meeting the new Canadian 24-Hour Movement Guidelines recommendations according to body mass index classes in Canadian adults. Health reports. 2022;33(11):3-15.

36.	Balbim GM, Falck RS, Boa Sorte Silva NC, Kramer AF, Voss M, Liu-Ambrose T. The association of the 24-hour activity cycle profiles with cognition in older adults with mild cognitive impairment: A cross-sectional study. The journals of gerontology Series A, Biological sciences and medical sciences. 2024((Balbim, Falck, Boa Sorte Silva, Liu-Ambrose) Djavad Mowafaghian Centre for Brain Health, University of British Columbia, Vancouver, Canada(Balbim, Falck, Boa Sorte Silva, Liu-Ambrose) Centre for Aging SMART at Vancouver Coastal Health, Vancouver Coastal).

37.	Bang F, Roberts KC, Chaput JP, Goldfield GS, Prince SA. Physical activity, screen time and sleep duration: Combined associations with psychosocial health among Canadian children and youth. Health reports. 2020;31(5):9-16.

38.	Bao R, Qin H, Memon AR, Chen S, López-Gil JF, Liu S, et al. Is adherence to the 24-h movement guidelines associated with greater academic-related outcomes in children and adolescents? A systematic review and meta-analysis. European Journal of Pediatrics. 2024;183(5):2003-14.

39.	Bao R, Yang Z, Memon AR, Chen S, Wang L, Cai Y. Association between meeting the 24-h movement guidelines and psychosocial health in children: A cross-sectional study. Child: Care, Health and Development. 2024;50(1).

40.	Barone Gibbs B, Kline CE, Huber KA, Paley JL, Perera S. Covid-19 shelter-at-home and work, lifestyle and well-being in desk workers. Occupational Medicine. 2021;71(2):86-94.

41.	Bates LC, Zieff G, Stanford K, Moore JB, Kerr ZY, Hanson ED, et al. COVID-19 Impact on Behaviors across the 24-Hour Day in Children and Adolescents: Physical Activity, Sedentary Behavior, and Sleep. Children. 2020;7(9):1-9.

42.	Bauman A, Bittman M, Gershuny J. A short history of time use research; implications for public health. BMC public health. 2019;19(Supplement 2):607.

43.	Bejarano G, Brayton RP, Ranjit N, Hoelscher DM, Brown D, Knell G. Weight status and meeting the physical activity, sleep, and screen-time guidelines among Texas children: results from a population based, cross-sectional analysis. BMC Pediatrics. 2022;22(1).

44.	Berglind D, Ljung R, Tynelius P, Brooke HL. Cross-sectional and prospective associations of meeting 24-h movement guidelines with overweight and obesity in preschool children. Pediatric Obesity. 2018;13(7):442-9.

45.	Berninger N, Knell G, Gabriel KP, Plasqui G, Crutzen R, Hoor GT. Bidirectional Day-to-Day Associations of Reported Sleep Duration With Accelerometer Measured Physical Activity and Sedentary Time Among Dutch Adolescents: An Observational Study. Journal for the measurement of physical behaviour. 2020;3(4):304-14.

46.	Bezerra TA, Clark CCT, de Souza Filho AN, de Souza Fortes L, Silva Mota JAP, Duncan MJ, et al. 24-hour movement behaviour and executive function in preschoolers: a compositional and isotemporal reallocation analysis. European Journal of Sport Science. 2020:1-18.

47.	Bianchim MS, McNarry MA, Holland A, Cox NS, Dreger J, Barker AR, et al. A Compositional Analysis of Physical Activity, Sedentary Time, and Sleep and Associated Health Outcomes in Children and Adults with Cystic Fibrosis. International Journal of Environmental Research and Public Health. 2022;19(9).

48.	Bianconi A, Fiore M, Zauli E, Acuti Martellucci C, Rosso A, Dallolio L, et al. How strong is the evidence supporting the WHO guidelines on physical activity, sedentary behaviour and sleep in early childhood? European Journal of Clinical Investigation. 2024.

49.	Biddle GJH, Edwardson CL, Henson J, Davies MJ, Khunti K, Rowlands AV, et al. Associations of physical behaviours and behavioural reallocations with markers of metabolic health: A compositional data analysis. International Journal of Environmental Research and Public Health. 2018;15(10):2280.

50.	Bird M, Datta GD, Chinerman D, Kakinami L, Mathieu M-E, Henderson M, et al. Associations of neighborhood walkability with moderate to vigorous physical activity: an application of compositional data analysis comparing compositional and non-compositional approaches. International Journal of Behavioral Nutrition & Physical Activity. 2022;19(1):1-10.

51.	Blodgett JM, Ahmadi MN, Atkin AJ, Chastin S, Chan HW, Suorsa K, et al. Device-measured physical activity and cardiometabolic health: The Prospective Physical Activity, Sitting, and Sleep (ProPASS) consortium. European Heart Journal. 2024;45(6):458-71.

52.	Blodgett JM, Bann D, Chastin SFM, Ahmadi M, Stamatakis E, Cooper R, et al. Socioeconomic gradients in 24-hour movement patterns across weekends and weekdays in a working-age sample: Evidence from the 1970 British Cohort Study. Journal of Epidemiology and Community Health. 2024;78(8):515-21.

53.	Blodgett JM, Mitchell JJ, Stamatakis E, Chastin S, Hamer M. Associations between the composition of daily time spent in physical activity, sedentary behaviour and sleep and risk of depression: Compositional data analyses of the 1970 British cohort Study. Journal of Affective Disorders. 2023;320:616-20.

54.	Bogaert L, Willems I, Calders P, Dirinck E, Kinaupenne M, Decraene M, et al. Explanatory variables of objectively measured 24-h movement behaviors in people with prediabetes and type 2 diabetes: A systematic review. Diabetes & metabolic syndrome. 2024;18(4):102995.

55.	Booker R, Holmes ME, Newton RL, Norris KC, Thorpe RJ, Carnethon MR, et al. Compositional analysis of movement behaviors' association on high-sensitivity c-reactive protein: the Jackson heart study. Annals of Epidemiology. 2022;76:7-12.

56.	Boudreaux BD, Frederick GM, Oconnor PJ, Evans EM, Schmidt MD. Harmonization of three different accelerometers to classify the 24 h activity cycle. Physiological Measurement. 2024;45(4).

57.	Brady SM, Salway R, Mariapun J, Millard L, Ramadas A, Rizal H, et al. Accelerometer-measured 24-hour movement behaviours over 7 days in Malaysian children and adolescents: A cross-sectional study. PLoS ONE. 2024;19(2 FEBRUARY):e0297102.

58.	Brakenridge CJ, Healy GN, Sethi P, Carver A, Bellettiere J, Salim A, et al. Contrasting compositions of sitting, standing, stepping, and sleeping time: Associations with glycaemic outcome by diabetes risk. The International Journal of Behavioral Nutrition and Physical Activity. 2021;18.

59.	Brakenridge CJ, Koster A, de Galan BE, Carver A, Dumuid D, Dzakpasu FQS, et al. Associations of 24 h time-use compositions of sitting, standing, physical activity and sleeping with optimal cardiometabolic risk and glycaemic control: The Maastricht Study. Diabetologia. 2024;67(7):1356-67.

60.	Brayton RP, Price AM, Jones C, Ellis C, Burkhart S, Knell G. Prospective evaluation of 24-hour movement behaviors among adolescents recovering from a sport-related concussion. Applied neuropsychology Child. 2023((Brayton) Research Staff, Department of Epidemiology, Human Genetics, Environmental Sciences, School of Public Health, University of Texas Health Science Center at Houston (UTHealth), Dallas, TX, United States(Price) Concussion Program Director, Sports Ne):1-9.

61.	Brown DMY, Burkart S, Groves CI, Balbim GM, Pfledderer CD, Porter CD, et al. A systematic review of research reporting practices in observational studies examining associations between 24-h movement behaviors and indicators of health using compositional data analysis. Journal of Activity, Sedentary and Sleep Behaviors. 2024;3(1):23-.

62.	Brown DMY, Cairney J, Kwan MY. Adolescent movement behaviour profiles are associated with indicators of mental wellbeing. Mental Health and Physical Activity. 2021;20.

63.	Brown DMY, Faulkner GEJ, Kwan MYW. Healthier movement behavior profiles are associated with higher psychological wellbeing among emerging adults attending post-secondary education. Journal of Affective Disorders. 2022;319((Brown) Department of Psychology, The University of Texas at San Antonio, 1 UTSA Circle, San Antonio, TX 78254, United States(Faulkner) School of Kinesiology, University of British Columbia, 6081 University Blvd, Vancouver, BC V6T 1Z1, Canada(Kwan) Depart):511-7.

64.	Brown DMY, Hill RM, Wolf JK. Cross-sectional associations between 24-h movement guideline adherence and suicidal thoughts among Canadian post-secondary students. Mental Health and Physical Activity. 2022;23((Brown, Hill) Department of Psychology, The University of Texas at San Antonio, 1 UTSA Circle, San Antonio, TX 78249, United States(Wolf) Department of Sociology, The University of Texas at San Antonio, 1 UTSA Circle, San Antonio, TX 78249, United States):100484.

65.	Brown DMY, Kwan MY, Arbour-Nicitopoulos KP, Cairney J. Identifying patterns of movement behaviours in relation to depressive symptoms during adolescence: A latent profile analysis approach. Preventive Medicine. 2021;143.

66.	Brown DMY, Kwan MYW. Movement Behaviors and Mental Wellbeing: A Cross-Sectional Isotemporal Substitution Analysis of Canadian Adolescents. Frontiers in Behavioral Neuroscience. 2021;15.

67.	Brown DMY, McPhee PG, Kwan MY, Timmons BW. Implications of Disability Severity on 24-Hour Movement Guideline Adherence Among Children With Neurodevelopmental Disorders in the United States. Journal of physical activity & health. 2021;18(11):1325-31.

68.	Brown DMY, Ronen GM. Associations between 24-hour movement guideline adherence and mental health disorders among young people with active and inactive epilepsy. Epilepsy and Behavior. 2021;125((Brown) Department of Psychology, University of Texas at San Antonio, 1 UTSA Circle, San Antonio, TX 78249, United States(Ronen) CanChild Centre for Childhood Disability Research, 1400 Main Street West, Room 408, Hamilton, ON L8S 1C7, Canada(Ronen) Depart):108386.

69.	Bruijns BA, Bourke M, Loh A, Tucker P. Movement behaviour education for parents in prenatal, postnatal, and pediatric care in Canada: A needs assessment. BMC Pediatrics. 2024;24(1).

70.	Bruijns BA, Bourke M, Saravanamuttoo K, Tucker P. Physical activity, sedentary behaviour, and sleep knowledge and self-efficacy among parents of young children in Canada. Journal of activity, sedentary and sleep behaviors. 2024;3(1):12.

71.	Brusaca LA, Barbieri DF, Mathiassen SE, Holtermann A, Oliveira AB. Physical behaviours in brazilian office workers working from home during the COVID-19 pandemic, compared to before the pandemic: A compositional data analysis. International Journal of Environmental Research and Public Health. 2021;18(12):6278.

72.	Brusaca LA, Januario LB, Mathiassen SE, Barbieri DF, Oliveira RV, Heiden M, et al. Sedentary behaviour, physical activity, and sleep among office workers during the COVID-19 pandemic: a comparison of Brazil and Sweden. BMC Public Health. 2022;22(1):2196-.

73.	Brzęk A, Strauss M, Sanchis-Gomar F, Leischik R. Physical activity, screen time, sedentary and sleeping habits of polish preschoolers during the covid-19 pandemic and who’s recommendations: An observational cohort study. International Journal of Environmental Research and Public Health. 2021;18(21).

74.	Bu H, He A, Gong N, Huang L, Liang K, Kastelic K, et al. Optimal movement behaviors: correlates and associations with anxiety symptoms among Chinese university students. BMC Public Health. 2021;21(1):1-11.

75.	Buchan MC, Carson V, Faulkner G, Qian W, Leatherdale ST. Factors associated with students meeting components of canada's new 24-hour movement guidelines over time in the compass study. International Journal of Environmental Research and Public Health. 2020;17(15):1-15.

76.	Buchanan L, Xu H, Hewitt L, Taki S, Wen LM. A longitudinal analysis examining the associations of tummy time with active playtime, screen time, and sleep time. Journal of Physical Activity and Health. 2021;18(10):1215-22.

77.	Bucko AG, Dowda M, Pate RR. Factors Related to High-School Students’ Odds of Having Overweight or Obesity. American Journal of Lifestyle Medicine. 2022.

78.	Buman MP, Winkler EAH, Kurka JM, Hekler EB, Baldwin CM, Owen N, et al. Reallocating time to sleep, sedentary behaviors, or active behaviors: Associations with cardiovascular disease risk biomarkers, NHANES 2005-2006. American Journal of Epidemiology. 2014;179(3):323-34.

79.	Burge AT, Palarea-Albaladejo J, Holland AE, Abramson MJ, McDonald CF, Mahal A, et al. The Impact of Pulmonary Rehabilitation on 24-Hour Movement Behavior in People With Chronic Obstructive Pulmonary Disease: New Insights From a Compositional Perspective. Journal of physical activity & health. 2020;18(1):13-20.

80.	Burns RD. Public health implications of replacing screen time with physical activity and sleep in Brazilian children. Jornal de Pediatria. 2024;100(2):121-3.

81.	Burns RD, Almes H, Fu Y. Associations of activity, sedentary and sleep behaviors with oral health indictors in children and adolescents: a cross-sectional analysis. Journal of Activity, Sedentary and Sleep Behaviors. 2024;3(1).

82.	Burns RD, Bai Y, Pfledderer CD, Brusseau TA, Byun W. Movement behaviors and perceived loneliness and sadness within alaskan adolescents. International Journal of Environmental Research and Public Health. 2020;17(18):1-13.

83.	Burns RD, Byun W, Fu Y, Mihalopoulos NL. Sexual identity-behavior discordance and meeting 24-hour movement behavior recommendations in adolescents. Journal of LGBT Youth. 2024;21(1):1-19.

84.	Burns RD, Pfledderer CD, Fu Y. The neighbourhood social environment correlates with meeting 24-hmovement behaviour recommendations in females: A cross-sectional study using the 2019 national survey of children’s health. Applied Physiology, Nutrition and Metabolism. 2021;46(4):408-11.

85.	Burns RD, Pfledderer CD, Fu Y. Socioeconomic Factors, Movement Behavior Context, and Self-reported Physical and Mental Health in Adults Living in New York City. Health Behavior and Policy Review. 2023;10(5):1376-91.

86.	Butler GP, Roberts KC, Kropac E, Rao DP, Branchard B, Prince SA, et al. Conceptualizing a framework for the surveillance of physical activity, sedentary behaviour and sleep in Canada. Health Promotion and Chronic Disease Prevention in Canada. 2019;39(5):201-4.

87.	Byambaa A, Dechinjamts O, Jambaldorj B, Jones RA, Chong KH, Okely AD. Prevalence and Health Associations of Meeting the World Health Organization Guidelines for Physical Activity, Sedentary Behavior, and Sleep in Preschool-Aged Children: The SUNRISE Mongolia Pilot and Feasibility Study. Journal of physical activity & health. 2024;21(3):283-93.

88.	Byrne R, Terranova CO, Chai LK, Brookes DSK, Trost SG. Cognitive Testing of Items Measuring Movement Behaviours in Young Children Aged Zero to Five Years: Development of the Movement Behaviour Questionnaires for -Baby (MBQ-B) and -Child (MBQ-C). Children. 2023;10(9).

89.	Cabanas-Sánchez V, Esteban-Cornejo I, García-Esquinas E, Ortolá R, Ara I, Rodríguez-Gómez I, et al. Cross-sectional and prospective associations of sleep, sedentary and active behaviors with mental health in older people: a compositional data analysis from the Seniors-ENRICA-2 study. International Journal of Behavioral Nutrition & Physical Activity. 2021;18(1):1-13.

90.	Cabanas‐Sánchez V, Esteban‐Cornejo I, Migueles JH, Banegas JR, Graciani A, Rodríguez‐Artalejo F, et al. Twenty four‐hour activity cycle in older adults using wrist‐worn accelerometers: The seniors‐ENRICA‐2 study. Scandinavian Journal of Medicine & Science in Sports. 2020;30(4):700-8.

91.	Caetano IT, Miranda VPN, dos Santos FK, dos Santos Amorim PR. Adolescent's movement behaviors and built environment: a latent class analysis. BMC Public Health. 2021;21(1):1-14.

92.	Cai S, Zhong P, Dang J, Liu Y, Shi D, Chen Z, et al. Associations between combinations of 24‐h movement behaviors and physical fitness among Chinese adolescents: Sex and age disparities. Scandinavian Journal of Medicine & Science in Sports. 2023;33(9):1779-91.

93.	Caldwell HAT, Faulkner G, Tremblay MS, Rhodes RE, de Lannoy L, Kirk SFL, et al. Regional differences in movement behaviours of children and youth during the second wave of the COVID-19 pandemic in Canada: follow-up from a national study. Canadian journal of public health = Revue canadienne de sante publique. 2022;113(4):535-46.

94.	Campbell JE, Janssen I. The neighborhood physical environment and the 24-hour movement behavior composition among children. International Journal of Environmental Health Research. 2023;33(6):575-87.

95.	Campbell JE, Stone MR, Mitra R, Locke M, MacDonald C, Preston A, et al. Children and youth’s movement behaviours differed across phases and by geographic region throughout the COVID-19 pandemic in Nova Scotia, Canada: an explanatory sequential mixed-methods study. Journal of Activity, Sedentary and Sleep Behaviors. 2023;2(1).

96.	Cao C, Patel AV, Liu R, Cao Y, Friedenreich CM, Yang L. Trends and cancer-specific patterns of physical activity, sleep duration, and daily sitting time among US cancer survivors, 1997-2018. Journal of the National Cancer Institute. 2023;115(12):1563-75.

97.	Cao R, Gao T, Hu Y, Qin Z, Ren H, Liang L, et al. Clustering of lifestyle factors and the relationship with depressive symptoms among adolescents in Northeastern China. Journal of Affective Disorders. 2020;274:704-10.

98.	Capio CM, Jones RA, Ng CSM, Sit CHP, Chung KKH. Movement guidelines for young children: Engaging stakeholders to design dissemination strategies in the Hong Kong early childhood education context. Frontiers in public health. 2022;10((Capio, Ng, Chung) Early Childhood Education Department, Education University of Hong Kong, Hong Kong SAR, Tai Po, China(Capio) Centre for Psychosocial Health, Education University of Hong Kong, Hong Kong SAR, Tai Po, China(Capio) Health Science Departmen):1007209.

99.	Capio CM, Ng CSM, Chung KKH, Jones RA, Sit CHP. Evaluation of a school-based dissemination of the movement guidelines for young children in Hong Kong: Study protocol. Archives of Public Health. 2021;79(1):1-9.

100.	Cardon G, Chastin S, Stappen VV, Huys N, Stefanova T, Chakarova N, et al. Feel4Diabetes intervention: effectiveness on 24-hour physical behaviour composition in families at risk for diabetes development. Health Promotion International. 2022;37(5):1-15.

101.	Carson V, Adamo K, Rhodes RE. Associations of Parenthood with Physical Activity, Sedentary Behavior, and Sleep. American Journal of Health Behavior. 2018;42(3):80-9.

102.	Carson V, Chaput J-P, Janssen I, Tremblay MS. Health associations with meeting new 24-hour movement guidelines for Canadian children and youth. Preventive Medicine. 2017:7-13.

103.	Carson V, Ezeugwu VE, Tamana SK, Chikuma J, Lefebvre DL, Azad MB, et al. Associations between meeting the Canadian 24-Hour Movement Guidelines for the Early Years and behavioral and emotional problems among 3-year-olds. Journal of science and medicine in sport. 2019;22(7):797-802.

104.	Carson V, Faulkner G, Sabiston CM, Tremblay MS, Leatherdale ST. Patterns of movement behaviors and their association with overweight and obesity in youth. International Journal of Public Health (Springer Nature). 2015;60(5):551-9.

105.	Carson V, Tremblay MS, Chaput JP, Chastin SFM. Associations between sleep duration, sedentary time, physical activity, and health indicators among Canadian children and youth using compositional analyses. Applied Physiology, Nutrition and Metabolism. 2016;41(6):S294-S302.

106.	Carson V, Tremblay MS, Chastin SFM. Cross-sectional associations between sleep duration, sedentary time, physical activity, and adiposity indicators among Canadian preschool-aged children using compositional analyses. BMC Public Health. 2017;17.

107.	Carson V, Zhang Z, Boyd M, Potter M, Li J, Kuzik N, et al. Moderators of movement behaviour changes among Canadian toddlers and preschoolers throughout the first 2 years of the COVID-19 pandemic. Applied Physiology, Nutrition & Metabolism. 2024;49(9):1228-40.

108.	Carson V, Zhang Z, Predy M, Pritchard L, Hesketh KD. Adherence to Canadian 24-Hour Movement Guidelines among infants and associations with development: a longitudinal study. International Journal of Behavioral Nutrition and Physical Activity. 2022;19(1):154.

109.	Carson V, Zhang Z, Predy M, Pritchard L, Hesketh KD. Longitudinal associations between infant movement behaviours and development. International Journal of Behavioral Nutrition and Physical Activity. 2022;19(1).

110.	Caserta A, Reedman S, Morgan P, Williams CM. Physical activity and quality of life in children with idiopathic toe walking: a cross sectional study. BMC Pediatrics. 2022;22(1):1-8.

111.	Cellini N, McDevitt EA, Mednick SC, Buman MP. Free-living cross-comparison of two wearable monitors for sleep and physical activity in healthy young adults. Physiology & Behavior. 2016;157:79-86.

112.	Chang Z, Lei W. A Study on the Relationship Between Physical Activity, Sedentary Behavior, and Sleep Duration in Preschool Children. Frontiers in public health. 2021;9((Chang) College of Physical Education and Health, East China Normal University, Shanghai, China(Chang) Preschool Education School, Changsha Normal University, Hunan, China(Lei) College of Physical Education, Jiangxi Science and Technology Normal Universit):618962.

113.	Chao L, Ma R, Jiang W. Movement behaviours and anxiety symptoms in Chinese college students: A compositional data analysis. Frontiers in Psychology. 2022;13.

114.	Chaput J-P, Colley RC, Aubert S, Carson V, Janssen I, Roberts KC, et al. Proportion of preschool-aged children meeting the Canadian 24-Hour Movement Guidelines and associations with adiposity: results from the Canadian Health Measures Survey. BMC Public Health. 2017;17:147-54.

115.	Chaput J-P, Dutil C. Lack of sleep as a contributor to obesity in adolescents: Impacts on eating and activity behaviors. The International Journal of Behavioral Nutrition and Physical Activity. 2016;13.

116.	Chaput JP. The integration of pediatric sleep health into public health in Canada. Sleep Medicine. 2019;56:4-8.

117.	Chaput JP, Carson V, Gray CE, Tremblay MS. Importance of all movement behaviors in a 24 hour period for overall health. International Journal of Environmental Research and Public Health. 2014;11(12):12575-81.

118.	Chaput JP, Colley RC, Aubert S, Carson V, Janssen I, Roberts KC, et al. Proportion of preschool-aged children meeting the <i>Canadian</i> <i>24</i>-<i>Hour</i> <i>Movement</i> <i>Guidelines</i> and associations with adiposity: results from the Canadian Health Measures Survey. BMC PUBLIC HEALTH. 2017;17.

119.	Chaput JP, Leduc G, Boyer C, Belanger P, Le Blanc AG, Borghese MM, et al. Objectively measured physical activity, sedentary time and sleep duration: Independent and combined associations with adiposity in Canadian children. Nutrition and Diabetes. 2014;4(6):e117.

120.	Chaput JP, Saunders TJ, Carson V. Interactions between sleep, movement and other non-movement behaviours in the pathogenesis of childhood obesity. Obesity Reviews. 2017;18:7-14.

121.	Charman SJ, Blain AP, Trenell MI, Jakovljevic DG, Kunadian V. Physical activity, inactivity and sleep in older patients with coronary artery disease following percutaneous coronary intervention: a longitudinal, observational study. Coronary Artery Disease. 2023;34(6):441-7.

122.	Chastin S, McGregor D, Palarea-Albaladejo J, Diaz KM, Hagströmer M, Hallal PC, et al. Joint association between accelerometry-measured daily combination of time spent in physical activity, sedentary behaviour and sleep and all-cause mortality: A pooled analysis of six prospective cohorts using compositional analysis. British Journal of Sports Medicine. 2021;55(22):1277-85.

123.	Chemtob K, Reid RER, Guimarães RdF, Henderson M, Mathieu ME, Barnett TA, et al. Adherence to the 24‐hour movement guidelines and adiposity in a cohort of at risk youth: A longitudinal analysis. Pediatric Obesity. 2021;16(4):1-11.

124.	Chen B, Bernard JY, Padmapriya N, Ning Y, Cai S, Lanca C, et al. Associations between early-life screen viewing and 24 hour movement behaviours: findings from a longitudinal birth cohort study. The Lancet Child and Adolescent Health. 2020;4(3):201-9.

125.	Chen B, Bernard JY, Padmapriya N, Yao J, Goh C, Tan KH, et al. Socio-demographic and maternal predictors of adherence to 24-hour movement guidelines in Singaporean children. International Journal of Behavioral Nutrition & Physical Activity. 2019;16(1):N.PAG-N.PAG.

126.	Chen H, Wang LJ, Xin F, Liang G, Zhou YL. Associations between 24-h movement behaviours and BMI in Chinese primary- and middle- school students. Journal of Exercise Science and Fitness. 2023;21(2):186-92.

127.	Chen J, Fang X, Zhang F, Shen J, Liu Y, Xu P, et al. The associations of chronic pain and 24-h movement behaviors with incident mental disorders: evidence from a large-scale cohort study. BMC Medicine. 2024;22(1):313.

128.	Chen M, Chia M, Chua T, Shen Z, Kang M, Chen L, et al. Associations between Parental Educational Attainment, Children’s 24-h Behaviors and Children’s Hyperactivity Behavior in the COVID-19 Pandemic. Healthcare (Switzerland). 2024;12(5).

129.	Chen M, Chua T, Shen Z, Tay LY, Wang X, Chia M. The Associations between 24-Hour Movement Behaviours and Quality of Life in Preschoolers: A Compositional Analysis of Cross-Sectional Data from 2018-2021. International Journal of Environmental Research and Public Health. 2022;19(22):14969.

130.	Chen S, Brown D, Parker K, Lee EY. Trends in adherence to the 24-h movement guidelines among US adolescents from 2011 to 2019: Evidence from repeated cross-sectional cycles of the Youth Risk Behavior Surveillance System. Scandinavian Journal of Medicine and Science in Sports. 2024;34(4).

131.	Chen S, Hong J, Milton K, Klepac B, Ma J, Pedisic Z. Analysis of national physical activity and sedentary behaviour policies in China. BMC Public Health. 2023;23(1).

132.	Chen S, Hong J, Wang G, Liu Y. Move More, Sit Less and Sleep Well: An analysis of WHO movement guidelines for children under 5 years of age. Sports medicine and health science. 2021;3(1):54-7.

133.	Chen S, Liang K, Chen S, Huang L, Chi X. Association Between 24-Hour Movement Guideline and Physical, Verbal, and Relational Forms of Bullying Among Chinese Adolescents. Asia-Pacific Journal of Public Health. 2023;35(2/3):168-74.

134.	Chen S, Liang K, López‐Gil JF, Drenowatz C, Tremblay MS. Association between meeting 24‐h movement guidelines and academic performance in a sample of 67,281 Chinese children and adolescents. European Journal of Sport Science. 2024;24(4):487-98.

135.	Chen S, Yang L, Yang Y, Shi W, Stults-Kolehmainen M, Yuan Q, et al. Sedentary behavior, physical activity, sleep duration and obesity risk: Mendelian randomization study. PLoS ONE. 2024;19(3 March).

136.	Chen ST, Yan J. Prevalence and selected sociodemographic of movement behaviors in schoolchildren from low-and middle-income families in nanjing, china: A cross-sectional questionnaire survey. Children. 2020;7(2).

137.	Chen XK, Zheng C, Wong SHS, Ma ACH. Moderate-vigorous physical activity attenuates premature senescence of immune cells in sedentary adults with obesity: a pilot randomized controlled trial. Aging. 2022;14(24):10137-52.

138.	Chen Y, Chan S, Bennett D, Chen X, Wu X, Ke Y, et al. Device-measured movement behaviours in over 20,000 China Kadoorie Biobank participants. International Journal of Behavioral Nutrition & Physical Activity. 2023;20(1):1-11.

139.	Chen Y, Hou M, Herold F, Wang K, Werneck AO, Van Damme T, et al. Linking 24-hour movement behavior guidelines to academic engagement and social-emotional functioning among youth with chronic pain. Mental Health and Physical Activity. 2024;27.

140.	Chen Y, Lyu Y. Comprehensively review behavioral pattern leading to better health during childhood. Chinese Journal of School Health. 2019;40(12):1761-6.

141.	Chen Y, Song Y, Zhou N, Wang W, Hong X. Association between movement behavior patterns and cardiovascular risk among Chinese adults aged 40-75: a sex-specific latent class analysis. BMC public health. 2024;24(1):1170.

142.	Chen YY, Ke YL, Lyu J, Sun DJY, Pan L, Pei P, et al. Progress and practice of objective measurement of physical behaviors in large-scale cohort research. Zhonghua liu xing bing xue za zhi = Zhonghua liuxingbingxue zazhi. 2024;45(1):35-40.

143.	Chen Z, Chi G, Wang L, Chen S, Yan J, Li S. The Combinations of Physical Activity, Screen Time, and Sleep, and Their Associations with Self-Reported Physical Fitness in Children and Adolescents. International Journal of Environmental Research and Public Health. 2022;19(10).

144.	Cheng Y, Huang Y, Zhang G, Sun J. Educating adolescents on preventing substance use: The role of 24-h movement behaviors ─ data from 2021 Youth Risk Behavior Surveillance. Complementary Therapies in Clinical Practice. 2024;57.

145.	Chia MYH, Tay LY, Chua TBK. Quality of Life and Meeting 24-h WHO Guidelines Among Preschool Children in Singapore. Early Childhood Education Journal. 2020;48(3):313-23.

146.	Chong KH, Dumuid D, Cliff DP, Parrish AM, Okely AD. Changes in 24-Hour Domain-Specific Movement Behaviors and Their Associations With Children's Psychosocial Health During the Transition From Primary to Secondary School: A Compositional Data Analysis. Journal of physical activity & health. 2022;19(5):358-66.

147.	Chong KH, Parrish A-M, Cliff DP, Dumuid D, Okely AD. Changes in 24-hour movement behaviours during the transition from primary to secondary school among Australian children. European Journal of Sport Science. 2022;22(8):1276-86.

148.	Chong KH, Parrish A-M, Cliff DP, Kemp BJ, Zhang Z, Okely AD. Changes in physical activity, sedentary behaviour and sleep across the transition from primary to secondary school: A systematic review. Journal of Science & Medicine in Sport. 2020;23(5):498-505.

149.	Chong KH, Parrish AM, Cliff DP, Dumuid D, Okely AD. Cross-sectional and longitudinal associations between 24-hour movement behaviours, recreational screen use and psychosocial health outcomes in children: A compositional data analysis approach. International Journal of Environmental Research and Public Health. 2021;18(11):5995.

150.	Christian H, Murray K, Trost SG, Schipperijn J, Trapp G, Maitland C, et al. Meeting the Australian 24-Hour Movement Guidelines for the Early Years is associated with better social-emotional development in preschool boys. Preventive medicine reports. 2022;27(101643766):101770.

151.	Christian H, Wenden EJ, Ng M, Maitland C. Association between preschooler movement behaviours, family dog ownership, dog play and dog walking: Findings from the PLAYCE study. Preventive Medicine Reports. 2022;26.

152.	Christofaro DGD, Ferrari G, Cucato GG, Mota J, Silva DR, Vanderlei LCM, et al. Association of meeting the 24-h movement guidelines with high blood pressure in adolescents: a cross-sectional study. Scientific Reports. 2024;14(1).

153.	Clarke AE, Carson V, Chaput J-P, Colley RC, Roberts KC, Rollo S, et al. Meeting Canadian 24-Hour Movement Guideline recommendations and risk of all-cause mortality. Applied Physiology, Nutrition & Metabolism. 2021;46(12):1487-94.

154.	Clarke AE, Janssen I. A compositional analysis of time spent in sleep, sedentary behaviour and physical activity with all-cause mortality risk. International Journal of Behavioral Nutrition and Physical Activity. 2021;18(1).

155.	Clevenger KA, Montoye AHK. Systematic Review of Accelerometer Responsiveness to Change for Measuring Physical Activity, Sedentary Behavior, or Sleep. Journal for the Measurement of Physical Behaviour. 2023;6(4):250-63.

156.	Cliff DP, Antczak D, Draper CE, Olds T, Santos R, Silva DAS, et al. The sleep and activity database for the early years (SADEY) study: design and methods. Journal of Activity, Sedentary and Sleep Behaviors. 2024;3(1).

157.	Cliff DP, McNeill J, Vella SA, Howard SJ, Santos R, Batterham M, et al. Adherence to 24-Hour Movement Guidelines for the Early Years and associations with social-cognitive development among Australian preschool children. BMC Public Health. 2017;17:207-15.

158.	Coats JC, Coxon M, Temple VA, Butler C, Stuart-Hill L. Examining the Canadian 24-Hour Movement Guidelines among Adults with Intellectual Disability: A Pilot Study. International Journal of Environmental Research and Public Health. 2023;20(13):6291.

159.	Cole AG, Laxer RE, Patte KA, Leatherdale ST. Can we reverse this trend? Exploring health and risk behaviours of grade 12 cohorts of Ontario students from 2013–2019. International Journal of Environmental Research and Public Health. 2021;18(6):1-14.

160.	Colley RC, Michaud I, Garriguet D. Reallocating time between sleep, sedentary and active behaviours: Associations with obesity and health in Canadian adults. Health Reports. 2018;29(4):1-13.

161.	Collings PJ, Backes A, Aguayo GA, Fagherazzi G, Malisoux L. Substituting device-measured sedentary time with alternative 24-hour movement behaviours: compositional associations with adiposity and cardiometabolic risk in the ORISCAV-LUX 2 study. Diabetology and Metabolic Syndrome. 2023;15(1):70.

162.	Collings PJ, Backes A, Malisoux L. Arterial stiffness and the reallocation of time between device-measured 24-hour movement behaviours: A compositional data analysis. Atherosclerosis. 2023;379((Collings, Backes, Malisoux) Physical Activity, Sport and Health Research Group, Department of Precision Health, Luxembourg Institute of Health, Luxembourg):117185.

163.	Conners RT, Bates LC, Lassalle PP, Zieff G, Whitehead PN, Stevens S, et al. Current and Future Implications of COVID-19 among Youth Wheelchair Users: 24-Hour Activity Behavior. Children. 2021;8(8):1-6.

164.	Costa RM, Minatto G, Costa BGG, Silva KS. Clustering of 24-h movement behaviors associated with cardiorespiratory fitness among adolescents: a latent class analysis. European Journal of Pediatrics. 2021;180(1):109-17.

165.	Coyle-Asbil HJ, Breau B, Ma DWL, Haines J, Buchholz AC, Vallis LA. Compliance with the 24-hour movement behavior guidelines and the impact of sleep methods among toddler, preschool, and school-aged children enrolled in the Guelph Family Health Study. Journal of Science and Medicine in Sport. 2024;27(9):631-9.

166.	Crowley P, Kildedal R, Vindelev SO, Jacobsen SS, Larsen JR, Johansson PJ, et al. A Novel System for the Device-Based Measurement of Physical Activity, Sedentary Behavior, and Sleep (Motus): Usability Evaluation. JMIR formative research. 2023;7(101726394):e48209.

167.	Cruz BdP, McGregor DE, Cruz JdP, Buman MP, Palarea-Albaladejo J, Alfonso-Rosa RM, et al. Integrating Sleep, Physical Activity, and Diet Quality to Estimate All-Cause Mortality Risk: A Combined Compositional Clustering and Survival Analysis of the National Health and Nutrition Examination Survey 2005–2006 Cycle. American Journal of Epidemiology. 2020;189(10):1057-64.

168.	Cuberek R, Pelclová J, Gába A, Pechová J, Svozilová Z, Přidalová M, et al. Adiposity and changes in movement-related behaviors in older adult women in the context of the built environment: a protocol for a prospective cohort study. BMC Public Health. 2019;19(1).

169.	Cui Q, Chen Y, Ye X, Cai Y, Qin R, Chen T, et al. Patterns of Lifestyle Behaviors and Relevant Metabolic Profiles in Chinese Adults: Latent Class Analysis from Two Independent Surveys in Urban and Rural Populations. Iranian Journal of Public Health. 2022;51(5):1076-83.

170.	Curtis RG, Dumuid D, McCabe H, Singh B, Ferguson T, Maher C. The association between 24-hour activity, sedentary and sleep compositions and mental health in Australian adults: a cross-sectional study. Journal of Activity, Sedentary and Sleep Behaviors. 2023;2(1).

171.	Curtis RG, Dumuid D, Olds T, Plotnikoff R, Vandelanotte C, Ryan J, et al. The Association Between Time-Use Behaviors and Physical and Mental Well-Being in Adults: A Compositional Isotemporal Substitution Analysis. Journal of physical activity & health. 2020;17(2):197-203.

172.	Curtis RG, Olds T, Ferguson T, Fraysse F, Dumuid D, Esterman A, et al. Changes in diet, activity, weight, and wellbeing of parents during COVID-19 lockdown. PLoS ONE. 2021;16(3 March).

173.	Curtis RG, Olds T, Fraysse F, Dumuid D, Hendrie GA, Esterman A, et al. Annual rhythms in adults' lifestyle and health (ARIA): protocol for a 12-month longitudinal study examining temporal patterns in weight, activity, diet, and wellbeing in Australian adults. BMC PUBLIC HEALTH. 2021;21(1).

174.	da Costa BGG, Chaput J-P, Lopes MVV, Malheiros LEA, Tremblay MS, Silva KS. Prevalence and sociodemographic factors associated with meeting the 24-hour movement guidelines in a sample of Brazilian adolescents. PloS one. 2020;15(9):e0239833.

175.	da Costa BGG, Chaput JP, Lopes MVV, Gaya AR, Silva DAS, Silva KS. Association between sociodemographic, dietary, and substance use factors and accelerometer-measured 24-hour movement behaviours in Brazilian adolescents. European Journal of Pediatrics. 2021;180(11):3297-305.

176.	da Costa BGG, Chaput JP, Lopes MVV, Malheiros LEA, da Silva ICM, Silva KS. Association between screen time and accelerometer-measured 24-h movement behaviors in a sample of Brazilian adolescents. Public Health (Elsevier). 2021;195:32-8.

177.	da Costa BGG, Chaput JP, Lopes MVV, Malheiros LEA, da Silva KS. Associations between sociodemographic, dietary, and substance use factors with self-reported 24-hour movement behaviors in a sample of brazilian adolescents. International Journal of Environmental Research and Public Health. 2021;18(5):1-13.

178.	da Costa BGG, Chaput JP, Lopes MVV, Malheiros LEA, Silva KS. How do adolescents with short sleep duration spend their extra waking hours? A device-based analysis of physical activity and sedentary behaviour in a Brazilian sample. Sleep Science. 2021;14:163-6.

179.	da Costa BGG, Chaput JP, Lopes MVV, Malheiros LEA, Silva KS. Movement behaviors and their association with depressive symptoms in Brazilian adolescents: A cross-sectional study. Journal of Sport and Health Science. 2022;11(2):252-9.

180.	da Silva DRP, Ohara D, da Silva-Grigoletto ME. The importance of an integrative view of behaviors adopted during the 24 hours of the day in physical activity and exercise interventions studies. Revista Brasileira de Cineantropometria e Desempenho Humano. 2019;21.

181.	da Silva Ferreira M, Nunes SAN, Papini CB, de Sousa TF. Analysis of the reallocation of time spent sitting, sleeping, and performing physical activities under negative self-rated heath of university students. Mundo da Saude. 2022;46:142-52.

182.	Dalziell A, Janssen X. Barriers and facilitators of physical activity, sedentary and sleep behaviours in 3 to 4-year-old children from low-income families: a study protocol. Journal of Activity, Sedentary and Sleep Behaviors. 2023;2(1).

183.	De Craemer M, Decraene M, Willems I, Buysse F, Van Driessche E, Verbestel V. Objective measurement of 24-hour movement behaviors in preschool children using wrist-worn and thigh-worn accelerometers. International Journal of Environmental Research and Public Health. 2021;18(18):9482.

184.	De Craemer M, McGregor D, Androutsos O, Manios Y, Cardon G. Compliance with 24-h movement behaviour guidelines among belgian pre-school children: The toybox-study. International Journal of Environmental Research and Public Health. 2018;15(10).

185.	De Craemer M, Verbestel V. Comparison of outcomes derived from the actigraph GT3X+ and the axivity AX3 accelerometer to objectively measure 24-hour movement behaviors in adults: A cross-sectional study. International Journal of Environmental Research and Public Health. 2022;19(1):271.

186.	De Craemer M, Verbestel V, Cardon G, Androutsos O, Manios Y, Chastin S. Correlates of meeting the physical activity, sedentary behavior, and sleep guidelines for the early years among belgian preschool children: The toybox-study. International Journal of Environmental Research and Public Health. 2020;17(19):1-15.

187.	de Fátima Guimarães R, Mathieu ME, Reid RER, Henderson M, Barnett TA. Physical activity, screen time, and sleep trajectories from childhood to adolescence: The influence of sex and body weight status. Journal of Physical Activity and Health. 2021;18(7):767-73.

188.	de Lannoy L, Barbeau K, Vanderloo LM, Goldfield G, Lang JJ, MacLeod O, et al. Evidence supporting a combined movement behavior approach for children and youth's mental health—A scoping review and environmental scan. Mental Health and Physical Activity. 2023;24:1-15.

189.	de Lucena Martins CM, Lemos LFGBP, de Souza Filho AN, Bezerra TA, Soares IAA, Mota JG, et al. Adherence to 24-hour movement guidelines in low-income Brazilian preschoolers and associations with demographic correlates. American journal of human biology : the official journal of the Human Biology Council. 2021;33(4):e23519.

190.	de Mello GT, Minatto G, Costa RM, Leech RM, Cao Y, Lee RE, et al. Clusters of 24-hour movement behavior and diet and their relationship with health indicators among youth: a systematic review. BMC public health. 2024;24(1):1080.

191.	De Moraes ACF, Medeiros-Oliveira VC, Burford K, Schaan BD, Bloch K, de Carvalho KMB, et al. Association Between Sleep Time and Pro- and Anti-Inflammatory Biomarkers Is Mediated by Abdominal Obesity Among Adolescents. Journal of physical activity & health. 2023;20(10):926-33.

192.	de Paula D, Crochemore-Silva I, Griep RH, Duncan BB, Schmidt MI. Accelerometry Measured Movement Behaviors in Middle-Aged and Older Adults: Cross-Sectional Analysis of the ELSA-Brasil Study. Journal of physical activity & health. 2023;20(11):1008-17.

193.	de Paula D, Feter N, dos Reis RCP, Griep RH, Duncan BB, Schmidt MI. Associations of activity, sedentary and sleep behaviors with prevalent steatotic liver disease in middle-aged and older adults: the ELSA-Brasil study. Journal of Activity, Sedentary and Sleep Behaviors. 2024;3(1).

194.	de Souza AA, Bandeira PFR, Vale SMCG, da Cunha GL, Mota JAPS, de Lucena Martins CM. Paths towards a healthier BMI among short and adequate sleepers: A pathway network analysis considering movement behaviors in low-income preschoolers. American journal of human biology : the official journal of the Human Biology Council. 2023;35(9):e23915.

195.	de Souza AA, Clark CCT, Mota J, Tassitano RM, Duncan MJ, Martins CML. Movement behaviors in short versus adequate nocturnal sleepers: A compositional analysis of preschoolers. American journal of human biology : the official journal of the Human Biology Council. 2022;34(5):e23694.

196.	Decraene M, Verbestel V, Cardon G, Iotova V, Koletzko B, Moreno LA, et al. Compliance with the 24-hour movement behavior guidelines and associations with adiposity in european preschoolers: Results from the toybox-study. International Journal of Environmental Research and Public Health. 2021;18(14):7499.

197.	del Pozo Cruz B, Alfonso-Rosa RM, McGregor D, Chastin SF, Palarea-Albaladejo J, del Pozo Cruz J. Sedentary behaviour is associated with depression symptoms: Compositional data analysis from a representative sample of 3233 US adults and older adults assessed with accelerometers. Journal of Affective Disorders. 2020;265:59-62.

198.	Delfino LD, Tebar WR, Christofaro DGD. Prevalence and association of compliance with the Canadian 24-hour movement guidelines with sociodemographic aspects in Brazilian adults: a cross-sectional epidemiological study. BMC public health. 2024;24(1):262.

199.	Dennis C-L, Birken CS, Brennenstuhl S, Wrottesley S, Marini F, Bell RC, et al. Describing 24-hour movement behaviours among preconception and recently pregnant Canadian parents: who do we need to target? Behavioral Medicine. 2023;49(1):83-95.

200.	DeSmet A, De Bourdeaudhuij I, Chastin S, Crombez G, Maddison R, Cardon G. Adults' Preferences for Behavior Change Techniques and Engagement Features in a Mobile App to Promote 24-Hour Movement Behaviors: Cross-Sectional Survey Study. JMIR mHealth and uHealth. 2019;7(12):e15707.

201.	Di Blasio A, Di Dalmazi G, Morano T, Bucci I, Verrocchio S, Grossi S, et al. Relationships between daily physical activity combinations and psychophysical health status of Italian breast cancer survivors. Home Health Care Services Quarterly. 2022;41(3):200-18.

202.	Di Fabio DR, Blomme CK, Smith KM, Welk GJ, Campbell CG. Adherence to physical activity guidelines in mid-pregnancy does not reduce sedentary time: An observational study. International Journal of Behavioral Nutrition and Physical Activity. 2015;12(1).

203.	Diamant É, Perez T, Drouin O. Nudging interventions to improve children's sleep, physical activity and sedentary behavior: A scoping review. Preventive Medicine. 2023;173.

204.	Ding Y, Xu X, Xin Z, Cao Q, Huang J, Wu X, et al. Associations of sleeping patterns and isotemporal substitution of other behavior with the prevalence of CKD in Chinese adults. Frontiers of Medicine. 2024;18(2):303-14.

205.	Dinkel D, Lu K, John J, Snyder K, Jacobson LT. A Cross-Sectional Examination of Physical Activity, Sedentary Time, and Sleep Between Adults With and Without Children in the Home Using the National Health and Nutrition Examination Survey. Journal of physical activity & health. 2021;18(4):391-9.

206.	Dogra S, Dunstan DW, Sugiyama T, Stathi A, Gardiner PA, Owen N. Active Aging and Public Health: Evidence, Implications, and Opportunities. Annual Review of Public Health. 2022;43:439-59.

207.	Dogra S, Patlan I, O'Neill C, Lewthwaite H. Recommendations for 24-Hour Movement Behaviours in Adults with Asthma: A Review of Current Guidelines. International journal of environmental research and public health. 2020;17(5).

208.	Domingues SF, da Silva CD, Faria FR, de Sá Souza H, dos Santos Amorim PR. Sleep, sedentary behavior, and physical activity in Brazilian adolescents: Achievement recommendations and BMI associations through compositional data analysis. PLoS ONE. 2022;17(4 April).

209.	Donnelly S, Buchan DS, McLellan G, Roberts R, Arthur R. Exploring the feasibility of a cluster pilot randomised control trial to improve children's 24-hour movement behaviours and dietary intake: Happy homework. Journal of Sports Sciences. 2023;41(19):1787-800.

210.	Dooley EE, Palta P, Wolff-Hughes DL, Martinez-Amezcua P, Staudenmayer J, Troiano RP, et al. Higher 24-h Total Movement Activity Percentile Is Associated with Better Cognitive Performance in U.S. Older Adults. Medicine and Science in Sports and Exercise. 2022;54(8):1317-25.

211.	Downing KL, del Pozo Cruz B, Sanders T, Zheng M, Hnatiuk JA, Salmon J, et al. Outdoor time, screen time and sleep reported across early childhood: concurrent trajectories and maternal predictors. International Journal of Behavioral Nutrition and Physical Activity. 2022;19(1):160.

212.	Downing KL, Hesketh KD, Timperio A, Salmon J, Moss K, Mishra G. Family history of non-communicable diseases and associations with weight and movement behaviours in Australian school-aged children: A prospective study. BMJ Open. 2020;10(11).

213.	Doyon M, Pelland-St-Pierre L, Allard C, Bouchard L, Perron P, Hivert MF. Associations of sleep duration, sedentary behaviours and energy expenditure with maternal glycemia in pregnancy. Sleep Medicine. 2020;65:54-61.

214.	Draper CE, Silubonde TM, Mukoma G, van Sluijs EMF. Evaluation of the dissemination of the south african 24-hour movement guidelines for birth to 5 years. International Journal of Environmental Research and Public Health. 2021;18(6):1-21.

215.	Draper CE, Silubonde TM, Mukoma G, van Sluijs EMF. Perceptions of the South African 24-Hour Movement Guidelines for Birth to 5 Years: A Qualitative Study. Journal of physical activity & health. 2022;19(1):4-11.

216.	Draper CE, Tomaz SA, Biersteker L, Cook CJ, Couper J, de Milander M, et al. The South African 24-Hour Movement Guidelines for Birth to 5 Years: An Integration of Physical Activity, Sitting Behavior, Screen Time, and Sleep. Journal of physical activity & health. 2020;17(1):109-19.

217.	Draper CE, Tomaz SA, Cook CJ, Jugdav SS, Ramsammy C, Besharati S, et al. Understanding the influence of 24-hour movement behaviours on the health and development of preschool children from low-income South African settings: the SUNRISE pilot study. South African journal of sports medicine. 2020;32(1):v32i1a8415.

218.	Du Z, Wang S, Bulloch G, Zhang F, Wang Y, Lai C, et al. Accelerometer-Measured Daily Behaviors That Mediate the Association Between Refractive Status and Depressive Disorders. Translational Vision Science and Technology. 2024;13(7):3.

219.	Dubuc MM, Berrigan F, Goudreault M, Beaudoin S, Turcotte S. Covid-19 impact on adolescent 24 h movement behaviors. International Journal of Environmental Research and Public Health. 2021;18(17):9256.

220.	Dumuid D, Lewis LK, Olds TS, Maher C, Bondarenko C, Norton L. Relationships between older adults' use of time and cardio-respiratory fitness, obesity and cardio-metabolic risk: A compositional isotemporal substitution analysis. Maturitas. 2018;110:104-10.

221.	Dumuid D, Maher C, Lewis LK, Stanford TE, Martín Fernández JA, Ratcliffe J, et al. Human development index, children's health-related quality of life and movement behaviors: a compositional data analysis. Quality of Life Research. 2018;27(6):1473-82.

222.	Dumuid D, Mellow ML, Olds T, Tregoweth E, Greaves D, Keage H, et al. Does APOE e4 Status Change How 24-Hour Time-Use Composition Is Associated with Cognitive Function? An Exploratory Analysis Among Middle-to-Older Adults. Journal of Alzheimer's disease : JAD. 2022;88(3):1157-65.

223.	Dumuid D, Olds T, Lange K, Edwards B, Lycett K, Burgner DP, et al. Goldilocks Days: optimising children's time use for health and well-being. Journal of Epidemiology and Community Health. 2022;76(3):301-8.

224.	Dumuid D, Olds T, Lewis LK, Martin-Fernández JA, Katzmarzyk PT, Barreira T, et al. Health-Related Quality of Life and Lifestyle Behavior Clusters in School-Aged Children from 12 Countries. Journal of Pediatrics. 2017;183:178-83.e2.

225.	Dumuid D, Olds T, Martín-Fernández JA, Lewis LK, Cassidy L, Maher C. Academic Performance and Lifestyle Behaviors in Australian School Children: A Cluster Analysis. Health Education and Behavior. 2017;44(6):918-27.

226.	Dumuid D, Olds T, Wake M, Rasmussen CL, Pedisic Z, Hughes JH, et al. Your best day: An interactive app to translate how time reallocations within a 24-hour day are associated with health measures. PLoS ONE. 2022;17(9 September):e0272343.

227.	Dumuid D, Pedisic Z, Palarea-Albaladejo J, Martin-Fernandez JA, Hron K, Olds T. Compositional data analysis in time-use epidemiology: What, why, how. International Journal of Environmental Research and Public Health. 2020;17(7):2220.

228.	Dumuid D, Pedišić Ž, Stanford TE, Martín-Fernández J-A, Hron K, Maher CA, et al. The compositional isotemporal substitution model: A method for estimating changes in a health outcome for reallocation of time between sleep, physical activity and sedentary behaviour. Statistical Methods in Medical Research. 2019;28(3):846-57.

229.	Dumuid D, Simm P, Wake M, Burgner D, Juonala M, Wu F, et al. The "Goldilocks Day" for Children's Skeletal Health: Compositional Data Analysis of 24-Hour Activity Behaviors. Journal of Bone and Mineral Research. 2020;35(12):2393-403.

230.	Dumuid D, Stanford TE, Martin-Fernández JA, Pedišić Ž, Maher CA, Lewis LK, et al. Compositional data analysis for physical activity, sedentary time and sleep research. Statistical Methods in Medical Research. 2018;27(12):3726-38.

231.	Dumuid D, Stanford TE, Pedišić Ž, Maher C, Lewis LK, Martín-Fernández JA, et al. Adiposity and the isotemporal substitution of physical activity, sedentary time and sleep among school-aged children: A compositional data analysis approach. BMC Public Health. 2018;18(1).

232.	Dumuid D, Wake M, Burgner D, Tremblay MS, Okely AD, Edwards B, et al. Balancing time use for children's fitness and adiposity: Evidence to inform 24-hour guidelines for sleep, sedentary time and physical activity. PLoS ONE. 2021;16(1 January):e0245501.

233.	Dumuid D, Wake M, Clifford S, Burgner D, Carlin JB, Mensah FK, et al. The Association of the Body Composition of Children with 24-Hour Activity Composition. Journal of Pediatrics. 2019;208:43-.

234.	Duncan MJ, Kuzik N, Silva DAS, Bélanger RE, Carson V, Chaput JP, et al. “Goldilocks days” for adolescent mental health: Movement behaviour combinations for well-being, anxiety and depression by gender. Mental Health and Physical Activity. 2024;26.

235.	Duncan MJ, Murphy L, Oftedal S, Fenwick MJ, Vincent GE, Fenton S. The associations between physical activity, sedentary behaviour, and sleep with mortality and incident cardiovascular disease, cancer, diabetes and mental health in adults: a systematic review and meta-analysis of prospective cohort studies. Journal of Activity, Sedentary and Sleep Behaviors. 2023;2(1).

236.	Duncan MJ, Oftedal S, Rebar AL, Murawski B, Short CE, Rayward AT, et al. Patterns of physical activity, sitting time, and sleep in Australian adults: A latent class analysis. Sleep Health. 2020;6(6):828-34.

237.	Duncan MJ, Riazi NA, Faulkner G, Gilchrist JD, Leatherdale ST, Patte KA. Changes in Canadian Adolescent Time Use and Movement Guidelines During the Early COVID-19 Outbreak: A Longitudinal Prospective Natural Experiment Design. Journal of physical activity & health. 2022;19(8):566-77.

238.	Duncan MJ, Riazi NA, Faulkner G, Gilchrist JD, Leatherdale ST, Patte KA. The association of physical activity, sleep, and screen time with mental health in Canadian adolescents during the COVID-19 pandemic: A longitudinal isotemporal substitution analysis. Mental Health and Physical Activity. 2022;23.

239.	Duncan S, Stewart T, Mackay L, Neville J, Narayanan A, Walker C, et al. Wear-time compliance with a dual-accelerometer system for capturing 24-h behavioural profiles in children and adults. International Journal of Environmental Research and Public Health. 2018;15(7):1296.

240.	Dygrýn J, Medrano M, Molina-Garcia P, Rubín L, Jakubec L, Janda D, et al. Associations of novel 24-h accelerometer-derived metrics with adiposity in children and adolescents. Environmental Health and Preventive Medicine. 2021;26(1).

241.	Eckermann S, Willan AR, Okely A, Tremblay MS, Coelli T. Robust cross-country comparison of children meeting 24-HR movement guidelines: an odds solution for binary effect efficiency measures. Journal of Public Health (Germany). 2022;30(8):2059-68.

242.	Edney S, Chua XH, Muller AM, Kui KY, Muller-Riemenschneider F. mHealth interventions targeting movement behaviors in Asia: A scoping review. Obesity reviews : an official journal of the International Association for the Study of Obesity. 2022;23(4):e13396.

243.	El Miedany Y, Toth M, Plummer S, El Gaafary M, Mahran S, Elwakil W. The development of the Egyptian 24-h movement guidelines for adults aged 50 years and older: an integration of sleep, sedentary behavior, and physical activity by the Egyptian Academy of Bone and Muscle Health. Egyptian Rheumatology and Rehabilitation. 2024;51(1).

244.	Elmesmari RA, Reilly JJ, Paton JY. 24-Hour Movement Behaviors in Children with Chronic Disease and Their Healthy Peers: A Case-Control Study. International Journal of Environmental Research and Public Health. 2022;19(5):2912.

245.	Engberg E, Ojala A, Paasio H, Lahti J, Koski P, Vehviläinen-Julkunen K, et al. Sociodemographic factors, parental mental health and movement behaviours in the early years: the SUNRISE Finland study protocol. Journal of Activity, Sedentary and Sleep Behaviors. 2024;3(1).

246.	Ezeugwu VE, Mandhane PJ, Hammam N, Brook JR, Tamana SK, Hunter S, et al. Influence of Neighborhood Characteristics and Weather on Movement Behaviors at Age 3 and 5 Years in a Longitudinal Birth Cohort. Journal of physical activity & health. 2021;18(5):571-9.

247.	Ezeugwu VE, Manns PJ. Sleep Duration, Sedentary Behavior, Physical Activity, and Quality of Life after Inpatient Stroke Rehabilitation. Journal of Stroke and Cerebrovascular Diseases. 2017;26(9):2004-12.

248.	Ezeugwu VE, Manns PJ. Compositional associations between movement-related behaviours and functional outcomes post-stroke. Disability and Rehabilitation. 2024.

249.	Fabre-Estremera B, Buno-Soto A, Garcia-Esquinas E, Cabanas-Sanchez V, Martinez-Gomez D, Rodriguez-Artalejo F, et al. Device-measured movement behaviors and cardiac biomarkers in older adults without major cardiovascular disease: the Seniors-ENRICA-2 study. European review of aging and physical activity : official journal of the European Group for Research into Elderly and Physical Activity. 2023;20(1):5.

250.	Fairclough SJ, Clifford L, Brown D, Tyler R. Characteristics of 24-hour movement behaviours and their associations with mental health in children and adolescents. Journal of activity, sedentary and sleep behaviors. 2023;2(1):11.

251.	Fairclough SJ, Dumuid D, Taylor S, Curry W, McGrane B, Stratton G, et al. Fitness, fatness and the reallocation of time between children's daily movement behaviours: An analysis of compositional data. International Journal of Behavioral Nutrition and Physical Activity. 2017;14(1).

252.	Fairclough SJ, Tyler R, Dainty JR, Dumuid D, Richardson C, Shepstone L, et al. Cross-sectional associations between 24-hour activity behaviours and mental health indicators in children and adolescents: A compositional data analysis. Journal of Sports Sciences. 2021;39(14):1602-14.

253.	Falck RS, Davis JC, Khan KM, Handy TC, Liu-Ambrose T. A Wrinkle in Measuring Time Use for Cognitive Health: How should We Measure Physical Activity, Sedentary Behaviour and Sleep? American Journal of Lifestyle Medicine. 2023;17(2):258-75.

254.	Fan H, Yan J, Yang Z, Liang K, Chen S. Cross-sectional associations between screen time and the selected lifestyle behaviors in adolescents. Frontiers in Public Health. 2022;10.

255.	Fanxing K, Gao Y, Herold F, Hossain MM, Yeung AS, Ng JL, et al. Relationships between physical activity, sleep, and screen time with academic performance and psychological functioning among US children and adolescents with depression. Complementary Therapies in Clinical Practice. 2023;53:N.PAG-N.PAG.

256.	Faria FRD, Barbosa D, Howe CA, Canabrava KLR, Sasaki JE, Amorim PRDS. Time-use movement behaviors are associated with scores of depression/anxiety among adolescents: A compositional data analysis. PLoS ONE. 2022;17(12 December):e0279401.

257.	Farrahi V, Kangas M, Walmsley R, Niemela M, Kiviniemi A, Puukka K, et al. Compositional Associations of Sleep and Activities within the 24-h Cycle with Cardiometabolic Health Markers in Adults. Medicine and science in sports and exercise. 2021;53(2):324-32.

258.	Farrahi V, Muhammad U, Rostami M, Oussalah M. AccNet24: A deep learning framework for classifying 24-hour activity behaviours from wrist-worn accelerometer data under free-living environments. International Journal of Medical Informatics. 2023;172.

259.	Farrahi V, Rostami M. Machine learning in physical activity, sedentary, and sleep behavior research. Journal of Activity, Sedentary and Sleep Behaviors. 2024;3(1).

260.	Farrahi V, Rostami M, Nauha L, Korpisaari M, Niemelä M, Jämsä T, et al. Replacing sedentary time with physical activity and sleep: Associations with cardiometabolic health markers in adults. Scandinavian Journal of Medicine & Science in Sports. 2023;33(6):907-20.

261.	Faught E, Walters AJ, Latimer-Cheung AE, Faulkner G, Jones R, Duggan M, et al. Optimal messaging of the Canadian 24-Hour Movement Guidelines for Adults aged 18–64 years and Adults aged 65 years and older. Applied Physiology, Nutrition & Metabolism. 2020;45:S125-S50.

262.	Faught EL, Qian W, Carson VL, Storey KE, Faulkner G, Veugelers PJ, et al. The longitudinal impact of diet, physical activity, sleep, and screen time on Canadian adolescents' academic achievement: An analysis from the COMPASS study. Preventive Medicine. 2019;125:24-31.

263.	Faulkner G, Weatherson K, Patte K, Qian W, Leatherdale ST. Are one-year changes in adherence to the 24-hour movement guidelines associated with flourishing among Canadian youth? Preventive Medicine. 2020;139:N.PAG-N.PAG.

264.	Feehan L, Xie H, Lu N, Li LC. Twenty-four hour physical activity, sedentary behaviour and sleep profiles in adults living with rheumatoid arthritis: a cross-sectional latent class analysis. Journal of Activity, Sedentary and Sleep Behaviors. 2024;3(1).

265.	Feehan LM, Lu N, Xie H, Li LC. Twenty-Four Hour Activity and Sleep Profiles for Adults Living with Arthritis: Habits Matter. Arthritis Care & Research. 2020;72(12):1678-86.

266.	Felin Fochesatto C, Cristi-Montero C, Ribeiro Bandeira PF, Brand C, Dias AF, Bandeira DR, et al. A network analysis involving mental difficulties, cognition, physical fitness, 24-hour movement components, fatness, and sociodemographic factors in children. Journal of Exercise Science & Fitness. 2023;21(4):416-23.

267.	Feng J, Huang WY, Lau PWC, Wong SH-S, Sit CH-P. Movement behaviors and mental health of caregivers of preschoolers in China during the COVID-19 pandemic. Preventive Medicine. 2022;155:N.PAG-N.PAG.

268.	Feng J, Huang WY, Sit CH-P, Reilly JJ, Khan A. Effectiveness of a parent-focused intervention targeting 24-hour movement behaviours in preschool-aged children: a randomised controlled trial. International Journal of Behavioral Nutrition & Physical Activity. 2024;21(1):1-12.

269.	Feng J, Huang WY, Sit CHP. Effectiveness of a Parent-Focused Intervention Targeting 24-H Movement Behaviors in Preschool-Aged Children: Study Protocol for a Randomized Controlled Trial. Frontiers in public health. 2022;10((Feng) Department of Sport, Physical Education and Health, Hong Kong Baptist University, Hong Kong SAR, Kowloon Tong, China(Huang) Centre for Health and Exercise Science Research, Department of Sport, Physical Education and Health, Hong Kong Baptist Unive):870281.

270.	Feng J, Khan A, Lau PWC, Huang WY. Parental role‐modeling on 24‐hour movement behaviors among preschoolers during the COVID‐19 pandemic. Scandinavian Journal of Medicine & Science in Sports. 2024;34(1):1-9.

271.	Feng J, Lau PWC, Shi L, Huang WY. Longitudinal Shifts in Movement Behaviors during the COVID-19 Pandemic: Relations to Posttraumatic Stress Disorder among University Students. International Journal of Environmental Research and Public Health. 2022;19(20):13449.

272.	Feng J, Zheng C, Sit CH-P, Reilly JJ, Huang WY. Associations between meeting 24-hour movement guidelines and health in the early years: A systematic review and meta-analysis. Journal of Sports Sciences. 2021;39(22):2545-57.

273.	Feng Z, Tan S, Liu Y, Song Y, Zhang G, Zhang C, et al. Establishment of an intervention model for adolescent obesity based on component isochronous substitution method. Chinese Journal of School Health. 2023;44(11):1641-4.

274.	Ferguson T, Curtis R, Fraysse F, Lagiseti R, Northcott C, Virgara R, et al. Annual, seasonal, cultural and vacation patterns in sleep, sedentary behaviour and physical activity: a systematic review and meta-analysis. BMC Public Health. 2021;21(1):1-14.

275.	Ferguson T, Curtis R, Fraysse F, Olds T, Dumuid D, Brown W, et al. Weather associations with physical activity, sedentary behaviour and sleep patterns of Australian adults: a longitudinal study with implications for climate change. International Journal of Behavioral Nutrition and Physical Activity. 2023;20(1):30.

276.	Ferguson T, Curtis R, Fraysse F, Olds T, Dumuid D, Brown W, et al. How do 24-h movement behaviours change during and after vacation? A cohort study. International Journal of Behavioral Nutrition and Physical Activity. 2023;20(1):24.

277.	Ferguson T, Curtis R, Fraysse F, Olds T, Dumuid D, Brown W, et al. The Annual Rhythms in Sleep, Sedentary Behavior, and Physical Activity of Australian Adults: A Prospective Cohort Study. Annals of behavioral medicine : a publication of the Society of Behavioral Medicine. 2024;58(4):286-95.

278.	Ferrari G, Alberico C, Drenowatz C, Kovalskys I, Gómez G, Rigotti A, et al. Prevalence and sociodemographic correlates of meeting the Canadian 24-hour movement guidelines among latin american adults: a multi-national cross-sectional study. BMC Public Health. 2022;22(1):1-11.

279.	Ferrari G, Cristi-Montero C, Drenowatz C, Kovalskys I, Gómez G, Rigotti A, et al. Meeting 24-h movement guidelines and markers of adiposity in adults from eight Latin America countries: the ELANS study. Scientific Reports. 2022;12(1).

280.	Feter N, de Paula D, Dos Reis RCP, Alvim Matos SM, Barreto SM, Duncan BB, et al. Association Between 24-Hour Movement Behavior and Cognitive Function in Brazilian Middle-Aged and Older Adults: Findings From the ELSA-Brasil. Innovation in aging. 2023;7(3):igad030.

281.	Fillon A, Lambert C, Tardieu M, Genin P, Larras B, Melsens P, et al. Impact of the COVID-19 confinement on movement behaviors among French young children: The ONAPS national survey. Minerva Pediatrics. 2024;76(4):478-86.

282.	Flood SM, Thompson B, Faulkner G, Vanderloo LM, Blackett B, Dolf M, et al. Development of a ParticipACTION App-Based Intervention for Improving Postsecondary Students' 24-Hour Movement Guideline Behaviors: Protocol for the Application of Intervention Mapping. JMIR research protocols. 2023;12(101599504):e39977.

283.	Foley L, Dumuid D, Atkin AJ, Olds T, Ogilvie D. Patterns of health behaviour associated with active travel: a compositional data analysis. International Journal of Behavioral Nutrition & Physical Activity. 2018;15:1-.

284.	Fonseca S, Coelho E, Mourao-Carvalhal MI. Compliance with 24-hour movement recommendations and health in university students during COVID-19 confinement. VIREF-REVISTA DE EDUCACION FISICA. 2022;11(1):1-12.

285.	Fournier E, Łuszczki E, Isacco L, Chanséaume-Bussiere E, Gryson C, Chambrier C, et al. Toward an Integrated Consideration of 24 h Movement Guidelines and Nutritional Recommendations. Nutrients. 2023;15(9):2109.

286.	Frederick GM, O'Connor PJ, Schmidt MD, Evans EM. Relationships between components of the 24-hour activity cycle and feelings of energy and fatigue in college students: A systematic review. Mental Health and Physical Activity. 2021;21((Frederick, O'Connor, Schmidt, Evans) Department of Kinesiology, University of Georgia, Athens, GA 30602, United States):100409.

287.	Frederick GM, Wilson OWA, Peterson KT. Associations between Meeting 24-hour Movement Guidelines and Perceived Stress and Depressive Symptoms among College Students: Differences by Gender, Race, and Sexual Orientation. International Journal of Kinesiology in Higher Education. 2023;7(4):309-21.

288.	Fu J, Sun S, Zhu S, Wang R, Chen D, Chen R, et al. Relationship between 24-h activity behavior and body fat percentage in preschool children: based on compositional data and isotemporal substitution analysis. BMC Public Health. 2024;24(1).

289.	Fu Y, Burns RD, Zhang P, Dyer S, Constantino N. Association of Adolescent Bullying Victimization with Meeting 24-hour Movement Behavior Recommendations: A Cross-Sectional Study Using the Combined 2015–2019 Youth Risk Behavior Survey. Journal of Science in Sport and Exercise. 2023;5(2):182-90.

290.	Full KM, Gallo LC, Malhotra A, Bellettiere J, Kerr J, Arredondo E, et al. Modeling the cardiometabolic benefits of sleep in older women: Exploring the 24-hour day. Sleep. 2020;43(1):zsz205.

291.	Full KM, Moran K, Carlson J, Godbole S, Natarajan L, Hipp A, et al. Latent profile analysis of accelerometer-measured sleep, physical activity, and sedentary time and differences in health characteristics in adult women. PLoS ONE. 2019;14(6):e0218595.

292.	Fung H, Yeo BTT, Chen C, Lo JC, Chee MWL, Ong JL. Adherence to 24-Hour Movement Recommendations and Health Indicators in Early Adolescence: Cross-Sectional and Longitudinal Associations in the Adolescent Brain Cognitive Development Study. Journal of Adolescent Health. 2023;72(3):460-70.

293.	Gába A, Dygrýn J, Štefelová N, Rubín L, Hron K, Jakubec L. Replacing school and out-of-school sedentary behaviors with physical activity and its associations with adiposity in children and adolescents: a compositional isotemporal substitution analysis. Environmental Health and Preventive Medicine. 2021;26(1).

294.	Gaba A, Dygryn J, Stefelova N, Rubin L, Hron K, Jakubec L, et al. How do short sleepers use extra waking hours? A compositional analysis of 24-h time-use patterns among children and adolescents. International Journal of Behavioral Nutrition and Physical Activity. 2020;17(1):104.

295.	Gallant F, Thibault V, Hebert J, Gunnell KE, Bélanger M. One size does not fit all: Identifying clusters of physical activity, screen time, and sleep behaviour co-development from childhood to adolescence. International Journal of Behavioral Nutrition and Physical Activity. 2020;17(1).

296.	Galmes-Panades AM, Konieczna J, Varela-Mato V, Abete I, Babio N, Fiol M, et al. Targeting body composition in an older population: do changes in movement behaviours matter? Longitudinal analyses in the PREDIMED-Plus trial. BMC Medicine. 2021;19(1).

297.	Galmes-Panades AM, Varela-Mato V, Konieczna J, Wärnberg J, Martínez-González MÁ, Salas-Salvadó J, et al. Isotemporal substitution of inactive time with physical activity and time in bed: Cross-sectional associations with cardiometabolic health in the PREDIMED-Plus study. International Journal of Behavioral Nutrition and Physical Activity. 2019;16(1).

298.	Gao W, Zhang Y, Wu D, Dong Y, Liu N, Wang H. Compliance with Health-Related Behaviors Guidelines and Its Relationship with Multiple Factors in Preschool Children Aged 3–6 Years: A National Cross-Sectional Survey in China. International Journal of Environmental Research and Public Health. 2022;19(3).

299.	Gao Y, Yu Q, Schuch FB, Herold F, Hossain MM, Ludyga S, et al. Meeting 24-h movement behavior guidelines is linked to academic engagement, psychological functioning, and cognitive difficulties in youth with internalizing problems. Journal of affective disorders. 2024;349(h3v, 7906073):176-86.

300.	Garcia J, Brewer B, Healy S, Patterson F. The temporal relationships between 24-h movement behaviors among children with autism spectrum disorder. Advances in Neurodevelopmental Disorders. 2022;6(2):157-65.

301.	Garcia JM, Leahy N, Rivera P, Brazendale K, Rice DJ. The association among demographic factors, health behaviors and sleep quality in youth with Autism Spectrum Disorder. Disability and Health Journal. 2020;13(3):100885.

302.	Garcia-Hermoso A, Ezzatvar Y, Alonso-Martinez AM, Ramirez-Velez R, Izquierdo M, Lopez-Gil JF. Twenty-four-hour movement guidelines during adolescence and its association with obesity at adulthood: results from a nationally representative study. European journal of pediatrics. 2023;182(3):1009-17.

303.	Garcia-Hermoso A, Ezzatvar Y, Lopez-Gil JF. Association Between Daily Physical Education Attendance and Meeting 24-Hour Movement Guidelines in Adolescence and Adulthood. The Journal of adolescent health : official publication of the Society for Adolescent Medicine. 2023;73(5):896-902.

304.	García-Hermoso A, Ezzatvar Y, Ramírez-Vélez R, López-Gil JF, Izquierdo M. Trajectories of 24-h movement guidelines from middle adolescence to adulthood on depression and suicidal ideation: a 22-year follow-up study. International Journal of Behavioral Nutrition and Physical Activity. 2022;19(1).

305.	Garcia-Hermoso A, Lopez-Gil JF, Ezzatvar Y, Ramirez-Velez R, Izquierdo M. Twenty-four-hour movement guidelines during middle adolescence and their association with glucose outcomes and type 2 diabetes mellitus in adulthood. Journal of sport and health science. 2023;12(2):167-74.

306.	Gauci J, Olds T, Maher C, Watson A, Fraysse F, Munzberg M, et al. Characteristics associated with differences in 24-hour device-measured and self-reported sleep, sedentary behaviour and physical activity in a sample of Australian primary school children. Journal of Activity, Sedentary and Sleep Behaviors. 2023;2(1).

307.	German C, Makarem N, Fanning J, Redline S, Elfassy T, McClain A, et al. Sleep, Sedentary Behavior, Physical Activity, and Cardiovascular Health: MESA. Medicine and Science in Sports and Exercise. 2021;53(4):724-31.

308.	Germano-Soares AH, Farah BQ, Da Silva JF, Barros MVG, Tassitano RM. Clustering of 24H movement behaviors associated with clinic blood pressure in older adults: a cross-sectional study. Journal of Human Hypertension. 2024;38(7):575-9.

309.	Gerritsen A, Hulst RY, van Rijssen IM, Obeid J, Pillen S, Gorter JW, et al. The temporal and bi-directional relationship between physical activity and sleep in ambulatory children with cerebral palsy. Disability & Rehabilitation. 2024;46(13):2821-7.

310.	Ghanamah R. Changes in Healthy Behaviors among Arab Israeli Children Diagnosed with ASD amid the Coronavirus Outbreak: Mothers’ Perceptions. Education Sciences. 2024;14(3).

311.	Ghanamah R, Eghbaria-Ghanamah H, Abu-Saleh N, Kitany S. Parents’ Perceptions of Changes in Sleep Duration, Physical Activity, and Sedentary Behavior in Arab Israeli Children during the COVID-19 Outbreak. International Journal of Environmental Research and Public Health. 2023;20(11).

312.	Gibson V, van der Merwe E, Coetzee BA. Early childhood practitioners’ awareness of gross motor milestone acquisition and movement guidelines. South African Journal of Childhood Education. 2024;14(1).

313.	Gilchrist JD, Battista K, Patte KA, Faulkner G, Carson V, Leatherdale ST. Effects of reallocating physical activity, sedentary behaviors, and sleep on mental health in adolescents. Mental Health and Physical Activity. 2021;20.

314.	Gilson ND, Mielke GI, Coombes JS, Feter N, Smith E, Duncan MJ, et al. VO<sub>2peak</sub> and 24-hour sleep, sedentary behavior, and physical activity in Australian truck drivers. SCANDINAVIAN JOURNAL OF MEDICINE & SCIENCE IN SPORTS. 2021;31(7):1574-8.

315.	Giurgiu M, Ketelhut S, Kubica C, Nissen R, Doster A-K, Thron M, et al. Assessment of 24-hour physical behaviour in adults via wearables: a systematic review of validation studies under laboratory conditions. International Journal of Behavioral Nutrition & Physical Activity. 2023;20(1):1-12.

316.	Giurgiu M, Nigg C, Fiedler J, Timm I, Rulf E, Bussmann JBJ, et al. The Assessment of 24-Hr Physical Behavior in Children and Adolescents via Wearables: A Systematic Review of Laboratory Validation Studies. Journal for the Measurement of Physical Behaviour. 2022;5(4):263-76.

317.	Giurgiu M, Nissen R, Müller G, Ebner‐Priemer UW, Reichert M, Clark B. Drivers of productivity: Being physically active increases yet sedentary bouts and lack of sleep decrease work ability. Scandinavian Journal of Medicine & Science in Sports. 2021;31(10):1921-31.

318.	Giurgiu M, Timm I, Becker M, Schmidt S, Wunsch K, Nissen R, et al. Quality Evaluation of Free-living Validation Studies for the Assessment of 24-Hour Physical Behavior in Adults via Wearables: Systematic Review. JMIR mHealth and uHealth. 2022;10(6):e36377.

319.	Godoy-Cumillaf A, Fuentes-Merino P, Farías-Valenzuela C, Duclos-Bastías D, Giakoni-Ramírez F, Bruneau-Chávez J, et al. The Association between Sedentary Behavior, Physical Activity, and Physical Fitness with Body Mass Index and Sleep Time in Chilean Girls and Boys: A Cross-Sectional Study. Children. 2023;10(6).

320.	Gomersall SR, Norton K, Maher C, English C, Olds TS. In search of lost time: When people undertake a new exercise program, where does the time come from? A randomized controlled trial. Journal of Science and Medicine in Sport. 2015;18(1):43-8.

321.	Gomez-Garcia M, Bia D, Zocalo Y. Physical activity, sedentary behavior and sleep time: Association with cardiovascular hemodynamic parameters, blood pressure and structural and functional arterial properties in childhood. Journal of Cardiovascular Development and Disease. 2021;8(6):62.

322.	Goncalves WSF, Byrne R, de Lira PIC, Viana MT, Trost SG. Psychometric properties of instruments to measure parenting practices and children's movement behaviors in low-income families from Brazil. BMC Medical Research Methodology. 2021;21(1):1-9.

323.	Goncalves WSF, Byrne R, de Lira PIC, Viana MT, Trost SG. Adherence to 24-hour movement guidelines among rural Brazalian preschool children: associations with parenting practices. International Journal of Behavioral Nutrition & Physical Activity. 2022;19(1):1-10.

324.	Granat M, Ahmadi M, Stamatakis E, Hamer M. The Effect of Using Anchored Wake Time to Derive 24-h Device Measured Circadian Physical Behavior Patterns. Scandinavian Journal of Medicine and Science in Sports. 2024;34(7).

325.	Grant VM, Tomayko EJ, Kingfisher RD. Sleep and physical activity patterns in urban American Indian children. American Journal of Health Behavior. 2020;44(1):67-75.

326.	Grgic J, Dumuid D, Bengoechea EG, Shrestha N, Bauman A, Olds T, et al. Health outcomes associated with reallocations of time between sleep, sedentary behaviour, and physical activity: A systematic scoping review of isotemporal substitution studies. International Journal of Behavioral Nutrition and Physical Activity. 2018;15(1).

327.	Groves CI, Huong C, Porter CD, Summerville B, Swafford I, Witham B, et al. Associations between 24-h movement behaviors and indicators of mental health and well-being across the lifespan: a systematic review. Journal of Activity, Sedentary and Sleep Behaviors. 2024;3(1).

328.	Guan H, Zhang Z, Wang B, Okely AD, Tong M, Wu J, et al. Proportion of kindergarten children meeting the WHO guidelines on physical activity, sedentary behaviour and sleep and associations with adiposity in urban Beijing. BMC Pediatrics. 2020;20(1).

329.	Guan KQ, Herold F, Owen N, Cheval B, Liu ZJ, Gerber M, et al. Prevalence and correlates of meeting 24-hour movement behavior guidelines among 8523 youth prescribed eyeglasses/contact lenses. COMPLEMENTARY THERAPIES IN CLINICAL PRACTICE. 2024;57.

330.	Guedes DP, Zuppa MA. Adherence to Combined Healthy Movement Behavior Guidelines among Adolescents: Effects on Cardiometabolic Health Markers. International journal of environmental research and public health. 2022;19(14).

331.	Guerrero MD, Barnes JD, Tremblay MS, Pulkki-Råback L. Typologies of family functioning and 24-h movement behaviors. International Journal of Environmental Research and Public Health. 2021;18(2):1-10.

332.	Guerrero MD, Barnes JD, Walsh JJ, Chaput J-P, Tremblay MS, Goldfield GS. 24-Hour Movement Behaviors and Impulsivity. Journal of Clinical Chiropractic Pediatrics. 2019;18(2):1608-.

333.	Guerrero MD, Moore S, Faulkner G, Roberts KC, Mitra R, Vanderloo LM, et al. Identifying Risk Profiles for Nonadherence to the 24-Hour Movement Guidelines for Children and Youth 6 Months Into the COVID-19 Pandemic. Pediatric Exercise Science. 2023;35(3):155-64.

334.	Guerrero MD, Vanderloo LM, Rhodes RE, Faulkner G, Moore SA, Tremblay MS. Canadian children's and youth's adherence to the 24-h movement guidelines during the COVID-19 pandemic: A decision tree analysis. Journal of Sport and Health Science. 2020;9(4):313-21.

335.	Guimarães RDF, Gilbert JA, Lemoyne J, Mathieu ME. Better health indicators of FitSpirit participants meeting 24-h movement guidelines for Canadian children and youth. Health Promotion International. 2021;36(3):836-45.

336.	Guo H, Ma J, Chua TBK, Tay LY, Chia MYH, Kim H. Associations between Parents’ Digital Media Habits, Engagement, Awareness, and Movement Guidelines among Preschool-Age Children: International Ipreschooler Surveillance Study. International Journal of Environmental Research and Public Health. 2022;19(17).

337.	Guo L, Zhang J, Zeng S, Li T, Deng X. Research progress of alleviating depression among children and adolescents based on 24 h movement behaviors. Chinese Journal of School Health. 2024;45(6):899-903.

338.	Gupta N, Dencker-Larsen S, Lund Rasmussen C, McGregor D, Rasmussen CDN, Thorsen SV, et al. The physical activity paradox revisited: A prospective study on compositional accelerometer data and long-term sickness absence. International Journal of Behavioral Nutrition and Physical Activity. 2020;17(1).

339.	Gupta N, Dumuid D, KorshØj M, JØrgensen MB, SØgaard K, Holtermann A. Is Daily Composition of Movement Behaviors Related to Blood Pressure in Working Adults? Medicine and Science in Sports and Exercise. 2018;50(10):2150-5.

340.	Gupta N, Hallman DM, Dumuid D, Vij A, Rasmussen CL, Jorgensen MB, et al. Movement behavior profiles and obesity: a latent profile analysis of 24-h time-use composition among Danish workers. International Journal of Obesity. 2020;44(2):409-17.

341.	Gupta N, Korshøj M, Dumuid D, Coenen P, Allesøe K, Holtermann A. Daily domain-specific time-use composition of physical behaviors and blood pressure. International Journal of Behavioral Nutrition & Physical Activity. 2019;16(1):N.PAG-N.PAG.

342.	Gutierrez-Espinoza H, Cassola-Cajiao M, Garzon-Ulloa E, Celi-Lalama D, Bastidas-Caldes C, Araya-Quintanilla F, et al. Lifestyle behavior of physiotherapy students from Ecuador upon admission to higher education: a cross-sectional study. Frontiers in sports and active living. 2024;6(101765780):1352144.

343.	Haegele JA, Zhu X. Movement behaviors, comorbidities, and health-related quality of life among adults with visual impairments. Disability & Rehabilitation. 2022;44(16):4361-7.

344.	Haegele JA, Zhu X, Healy S. Behavioral Correlates of Depression Among Adults with Visual Impairments. Journal of Visual Impairment & Blindness. 2021;115(5):403-13.

345.	Haegele JA, Zhu X, Healy S, Patterson F. Proportions of youth with visual impairments meeting 24-hr movement guidelines. Child: Care, Health and Development. 2020;46(3):345-51.

346.	Haegele JA, Zhu X, Healy S, Patterson F. The 24-Hour Movement Guidelines and Body Composition Among Youth Receiving Special Education Services in the United States. Journal of physical activity & health. 2021;18(7):838-43.

347.	Hakimi S, Kaur S, Ross-White A, Martin LJ, Rosenberg MW. A systematic review examining associations between physical activity, sedentary behaviour, and sleep duration with quality of life in older adults aged 65 years and above. Applied Physiology, Nutrition and Metabolism. 2023;48(2):97-162.

348.	Hakimi S, Martin LJ, Rosenberg MW. Impacts of physical activity, sedentary behaviour, and sleep on depression symptoms in Canadian older adults 65 years of age and above: a compositional data analysis of the Canadian Longitudinal Study on Aging. Journal of Activity, Sedentary and Sleep Behaviors. 2024;3(1).

349.	Hallman DM, Januario LB, Mathiassen SE, Heiden M, Svensson S, Bergstrom G. Working from home during the COVID-19 outbreak in Sweden: effects on 24-h time-use in office workers. BMC public health. 2021;21(1):528.

350.	Handler L, Tennant EM, Faulkner G, Latimer-Cheung AE. Perceptions of Inclusivity: The Canadian 24-Hour Movement Guidelines for Children and Youth. Adapted Physical Activity Quarterly. 2019;36(1):1-18.

351.	Hansen J, Hanewinkel R, Galimov A. Physical activity, screen time, and sleep: do German children and adolescents meet the movement guidelines? European journal of pediatrics. 2022;181(5):1985-95.

352.	Hao Y, Zhou X, Razman R, Peng S, Ahmad NS. Compliance with the 24-hour movement behaviour guidelines among children and adolescents with disabilities: a systematic review and meta-analysis. BMC public health. 2024;24(1):2357.

353.	Hartson KR, Huntington-Moskos L, Sears CG, Genova G, Mathis C, Ford W, et al. Use of Electronic Ecological Momentary Assessment Methodologies in Physical Activity, Sedentary Behavior, and Sleep Research in Young Adults: Systematic Review. Journal of medical Internet research. 2023;25(100959882):e46783.

354.	Haszard JJ, Jackson R, Morrison S, Meredith-Jones KA, Galland BC, Beebe DW, et al. Losing sleep influences dietary intake in children: a longitudinal compositional analysis of a randomised crossover trial. International Journal of Behavioral Nutrition & Physical Activity. 2024;21(1):1-11.

355.	Haszard JJ, Meredith-Jones K, Farmer V, Williams S, Galland B, Taylor R. Non-Wear Time and Presentation of Compositional 24-Hour Time-Use Analyses Influence Conclusions About Sleep and Body Mass Index in Children. Journal for the Measurement of Physical Behaviour. 2020;3(3):204-10.

356.	Hayes-Ortiz T, Suárez-Reyes M, Galgani JE, Zbinden-Foncea H, Fernández-Verdejo R. Time reallocation of physical behaviours induced by endurance exercise in physically active individuals. European Journal of Sport Science. 2023;23(8):1810-20.

357.	Healy S, Aigner CJ, Haegele JA, Patterson F. Meeting the 24-hr movement guidelines: An update on US youth with autism spectrum disorder from the 2016 National Survey of Children's Health. Autism Research. 2019;12(6):941-51.

358.	Healy S, Brewer B, Garcia J, Daly J, Patterson F. Sweat, Sit, Sleep: A Compositional Analysis of 24-hr Movement Behaviors and Body Mass Index among Children with Autism Spectrum Disorder. Autism Research. 2021;14(3):545-50.

359.	Healy S, Brewer B, Hoopes E, Paller A, Mayberry S, Maguire J, et al. Identifying the most proximal multi-level factors associated with meeting each of the 24-h movement behavior recommendations in a sample of autistic adults. Disability and health journal. 2022;15(4):101367.

360.	Healy S, Brewer B, Palmiere K, Daly J, McGuire J, Patterson F. 24-h movement behaviors among autistic adults: Differences by sex, age, and level of independence. Disability and Health Journal. 2021;14(4):101108.

361.	Healy S, Foley J, Haegele JA. Physical Activity, Screen Time, and Sleep Duration Among Youth With Chronic Health Conditions in the United States. American Journal of Health Promotion. 2020;34(5):505-11.

362.	Healy S, Patterson F, Williams E, Lozano AJ, Hanlon AL, Obrusnikova I. Rethinking daily movement behaviours of children with autism spectrum disorder: Meeting the 24-hour movement guidelines. European Journal of Adapted Physical Activity. 2020;13(8).

363.	Hedayatrad L, Stewart T, Paine S-J, Marks E, Walker C, Duncan S. Sociodemographic differences in 24-hour time-use behaviours in New Zealand children. International Journal of Behavioral Nutrition & Physical Activity. 2022;19(1):1-17.

364.	Henson J, Tziannou A, Rowlands AV, Edwardson CL, Hall AP, Davies MJ, et al. Twenty-four-hour physical behaviour profiles across type 2 diabetes mellitus subtypes. Diabetes, Obesity and Metabolism. 2024;26(4):1355-65.

365.	Hernández PO, Machacón LMN, Beltrán YH. Level of physical activity, sedentary behavior, and sleep in the early childhood population. Nutricion Hospitalaria. 2021;38(6):1149-54.

366.	Hesketh KD, Downing KL, Campbell K, Crawford D, Salmon J, Hnatiuk JA. Proportion of infants meeting the Australian 24-hour Movement Guidelines for the Early Years: data from the Melbourne InFANT Program. BMC Public Health. 2017;17:191-8.

367.	Hesketh KD, Downing KL, Galland BC, Nicholson JM, Taylor R, Orellana L, et al. Protocol for the Let's Grow randomised controlled trial: examining efficacy, cost-effectiveness and scalability of a m-Health intervention for movement behaviours in toddlers. BMJ open. 2022;12(3):e057521.

368.	Hesketh KR, Janssen X. Movement behaviours and adherence to guidelines: perceptions of a sample of UK parents with children 0–18 months. International Journal of Behavioral Nutrition & Physical Activity. 2022;19(1):1-7.

369.	Hewitt L, Frohmuller C, Wall J, Okely AD. Clinician and early childhood educator knowledge and advice given to parents regarding physical activity, screen time and sleep. An observational study. Journal of Pediatric Nursing. 2022;66:143-50.

370.	Hidde MC, Lyden K, Henry K, Leach HJ. Reallocating time to physical activity and sleep: associations with quality of life in cancer survivors. Supportive Care in Cancer. 2022;30(9):7527-34.

371.	Hidde MC, Lyden K, Henry K, Leach HJ. Reallocating Time to Physical Activity and Sleep: Associations with Body Mass Index in Cancer Survivors. International Journal of Behavioral Medicine. 2024;31(1):109-15.

372.	Hidding LM, Chinapaw MJM, Belmon LS, Altenburg TM. Co-creating a 24-hour movement behavior tool together with 9–12-year-old children using mixed-methods: MyDailyMoves. International Journal of Behavioral Nutrition & Physical Activity. 2020;17(1):1-12.

373.	Higgins S, Pomeroy A, Bates LC, Paterson C, Barone Gibbs B, Pontzer H, et al. Sedentary behavior and cardiovascular disease risk: An evolutionary perspective. Frontiers in Physiology. 2022;13((Higgins, Pomeroy, Bates, Paterson, Stoner) Department of Exercise and Sport Science, University of North Carolina at Chapel Hill, Chapel Hill, NC, United States(Barone Gibbs) Department of Epidemiology and Biostatistics, West Virginia University, Morgant):962791.

374.	Hinkley T, Timperio A, Watson A, Duckham RL, Okely AD, Cliff D, et al. Prospective associations with physiological, psychosocial and educational outcomes of meeting Australian 24-Hour Movement Guidelines for the Early Years. International Journal of Behavioral Nutrition & Physical Activity. 2020;17(1):1-12.

375.	Hjorth MF, Chaput JP, Damsgaard CT, Dalskov SM, Andersen R, Astrup A, et al. Low physical activity level and short sleep duration are associated with an increased cardio-metabolic risk profile: A longitudinal study in 8-11 year old Danish children. PLoS ONE. 2014;9(8):e104677.

376.	Hjorth MF, Chaput JP, Ritz C, Dalskov SM, Andersen R, Astrup A, et al. Fatness predicts decreased physical activity and increased sedentary time, but not vice versa: support from a longitudinal study in 8- to 11-year-old children. International Journal of Obesity. 2014;38(7):959-65.

377.	Hofman A, Voortman T, Ikram MA, Luik AI. Time spent in physical activity, sedentary behavior, and sleep: Associations with self-rated sleep quality in middle-aged and older adults. Sleep Health. 2022;8(6):701-4.

378.	Hofman A, Voortman T, Ikram MA, Luik AI. Substitutions of physical activity, sedentary behaviour and sleep: Associations with mental health in middle-aged and elderly persons. Journal of Epidemiology and Community Health. 2022;76(2):175-81.

379.	Hongjuan LI, Ting Z, Liu Z, Aibao WU, Yanhui GAO. Research on the Association Between Time Use and Physical Fitness of Children and Adolescents Based on the Activity Balance Model. (English). Journal of Beijing Sport University. 2023;46(4):76-84.

380.	Hörnberg K, Pomeroy J, Sandberg C, Ångström L, Södergren A, Sundström B. Isotemporal Substitution of Time Between Sleep and Physical Activity: Associations With Cardiovascular Risk Factors in Early Rheumatoid Arthritis. ACR Open Rheumatology. 2021;3(3):138-46.

381.	Hossain MS, Deeba IM, Hasan M, Kariippanon KE, Chong KH, Cross PL, et al. International study of 24-h movement behaviors of early years (SUNRISE): a pilot study from Bangladesh. Pilot and Feasibility Studies. 2021;7(1):176.

382.	Hossain MS, Raheem E, Okely AD. 24-hour movement guidelines and weight status among preschool-aged children in Bangladesh: A community-level cross-sectional study. Brain and Behavior. 2023;13(7):e3094.

383.	Hou M, Herold F, Cheval B, Owen N, Teychenne M, Gerber M, et al. Recent trends and disparities in 24-hour movement behaviors among US youth with mental, behavioral and neurodevelopmental conditions. Journal of Affective Disorders. 2024;367:58-66.

384.	Hou M, Herold F, Healy S, Haegele JA, Block ME, Ludyga S, et al. 24-Hour movement behaviors among visually impaired US children and adolescents. Mental Health and Physical Activity. 2023;25.

385.	Hou M, Herold F, Werneck AO, Teychenne M, Paoli AGD, Taylor A, et al. Associations of 24-hour movement behaviors with externalizing and internalizing problems among children and adolescents prescribed with eyeglasses/contact lenses. International journal of clinical and health psychology : IJCHP. 2024;24(1):100435.

386.	Howard M, Akhund SA. Parents’ knowledge, perceptions and support around appropriate physical activity, screen time and sleep time levels for children. International Journal of Child Care and Education Policy. 2024;18(1).

387.	Howie EK, Joosten J, Harris CJ, Straker LM. Associations between meeting sleep, physical activity or screen time behaviour guidelines and academic performance in Australian school children. BMC Public Health. 2020;20(1).

388.	Howie EK, Nelson A, McVeigh JA, Andres A. Physical Activity, Sedentary and Sleep Phenotypes in Women During the First Trimester of Pregnancy. Maternal & Child Health Journal. 2023;27(10):1834-45.

389.	Huang C, Yang Y, Lu C, Qin P, Jiang W, Ma J, et al. Associations of 24-hour movement behaviors with emotional and behavioral problems among Chinese adolescents. Social Psychiatry and Psychiatric Epidemiology. 2024.

390.	Huang J, Li X, Li G, Haegele JA, Zou L, Chen S, et al. Prevalence of meeting 24-hour movement guidelines and its associations with health indicators in people with disabilities: A systematic review and meta-analysis. Disability and health journal. 2024;17(3):101616.

391.	Huang J, Memon AR, Bao R, Fan H, Wang L, Liu Y, et al. 24-H movement behaviours research in Chinese population: A scoping review. Journal of Exercise Science and Fitness. 2024;22(4):397-405.

392.	Huang S, Gu Y, Ali SH, Xue J, Zhang R, Wen X. Association Between Adherence to 24-Hour Movement Guidelines and Noncommunicable Disease Risk in Chinese Adults: Prospective Cohort Study. JMIR Public Health and Surveillance. 2024;10.

393.	Huang S, Huang Y, Gu Y, Chen H, Lv R, Wu S, et al. Adherence to 24-Hour Movement Guidelines in Relation to the Risk of Overweight and Obesity Among Children and Adolescents. Journal of Adolescent Health. 2023;73(5):887-95.

394.	Huang SY, Li YZ, Zhang YR, Huang YY, Wu BS, Zhang W, et al. Sleep, physical activity, sedentary behavior, and risk of incident dementia: a prospective cohort study of 431,924 UK Biobank participants. Molecular Psychiatry. 2022;27(10):4343-54.

395.	Huang WY, Feng J, Zheng C, Jiao J, Wong SHS. Associations of social jetlag with physical activity and sedentary behaviour in children and adolescents: A systematic review and meta-analysis. Journal of sleep research. 2024;33(1):e13997.

396.	Huang WY, Ho RST, Tremblay MS, Wong SHS. Relationships of physical activity and sedentary behaviour with the previous and subsequent nights’ sleep in children and youth: A systematic review and meta-analysis. Journal of Sleep Research. 2021;30(6).

397.	Huang WY, Wong SHS, He G, Salmon J. Isotemporal substitution analysis for sedentary behavior and body mass index. Medicine and Science in Sports and Exercise. 2016;48(11):2135-41.

398.	Huang Z, Zhou Y, He J, Liu Y. Component analysis of relationship between 24-hour movement behavior and physical fitness in college students. Chinese Journal of School Health. 2023;44(10):1550-4.

399.	Hui SSc, Zhang R, Suzuki K, Naito H, Balasekaran G, Song JK, et al. The associations between meeting 24‐hour movement guidelines and adiposity in Asian Adolescents: The Asia‐Fit Study. Scandinavian Journal of Medicine & Science in Sports. 2021;31(3):763-71.

400.	Hulst RY, Gorter JW, Obeid J, Voorman JM, van Rijssen IM, Gerritsen A, et al. Accelerometer‐measured physical activity, sedentary behavior, and sleep in children with cerebral palsy and their adherence to the 24‐hour activity guidelines. Developmental Medicine & Child Neurology. 2023;65(3):393-405.

401.	Husu P, Tokola K, Vähä-Ypyä H, Sievänen H, Suni J, Kaikkonen KM, et al. Physical Activity, Sedentary Behavior, and Time in Bed Among Finnish Adults Measured 24/7 by Triaxial Accelerometry. Journal for the Measurement of Physical Behaviour. 2021;4(2):163-73.

402.	Husu P, Vähä-Ypyä H, Tokola K, Sievänen H, Mänttäri A, Kokko S, et al. Measurement of Physical Fitness and 24/7 Physical Activity, Standing, Sedentary Behavior, and Time in Bed in Working-Age Finns: Study Protocol for FINFIT 2021. Methods and Protocols. 2022;5(1).

403.	Hyodo K, Kitano N, Ueno A, Yamaguchi D, Watanabe Y, Noda T, et al. Association between intensity or accumulating pattern of physical activity and executive function in community-dwelling older adults: A cross-sectional study with compositional data analysis. Frontiers in Human Neuroscience. 2023;16((Hyodo, Kitano, Ueno, Yamaguchi, Watanabe, Noda, Nishida, Kai, Arao) Physical Fitness Research Institute, Meiji Yasuda Life Foundation of Health and Welfare, Tokyo, Japan):1018087.

404.	Hyunshik K, Jiameng M, Sunkyoung L, Ying G. Change in Japanese children's 24-hour movement guidelines and mental health during the COVID-19 pandemic. Scientific reports. 2021;11(1):22972.

405.	Izzicupo P, Di Baldassarre A, Ghinassi B, Reichert FF, Kokubun E, Nakamura FY. Can Off-Training Physical Behaviors Influence Recovery in Athletes? A Scoping Review. Frontiers in physiology. 2019;10(101549006):448.

406.	Jakubec L, Gaba A, Dygryn J, Rubin L, Simunek A, Sigmund E. Is adherence to the 24-hour movement guidelines associated with a reduced risk of adiposity among children and adolescents? BMC public health. 2020;20(1):1119.

407.	Janda D, Gaba A, Hron K, Arundell L, Contardo Ayala AM. Movement behaviour typologies and their associations with adiposity indicators in children and adolescents: a latent profile analysis of 24-h compositional data. BMC public health. 2024;24(1):1553.

408.	Janda D, Gába A, Vencálek O, Fairclough SJ, Dygrýn J, Jakubec L, et al. A 24-h activity profile and adiposity among children and adolescents: Does the difference between school and weekend days matter? PLoS ONE. 2023;18(5 May).

409.	Janssen I, Clarke AE, Carson V, Chaput JP, Giangregorio LM, Kho ME, et al. A systematic review of compositional data analysis studies examining associations between sleep, sedentary behaviour, and physical activity with health outcomes in adults. Applied physiology, nutrition, and metabolism = Physiologie appliquee, nutrition et metabolisme. 2020;45(10 Supplement 2):S248-S57.

410.	Janssen I, Roberts KC, Thompson W. Is adherence to the Canadian 24-Hour Movement Behaviour Guidelines for Children and Youth associated with improved indicators of physical, mental, and social health? Applied Physiology, Nutrition & Metabolism. 2017;42(7):725-31.

411.	Janssen I, Roberts KC, Thompson W. Adherence to the 24-hour movement guidelines among 10- to 17-year-old Canadians. Health Promotion and Chronic Disease Prevention in Canada. 2017;37(11):369-75.

412.	Janssen X, Fleming L, Kirk A, Rollins L, Young D, Grealy M, et al. Changes in physical activity, sitting and sleep across the COVID-19 national lockdown period in Scotland. International Journal of Environmental Research and Public Health. 2020;17(24):1-10.

413.	Janssen X, Martin A, Hughes AR, Hill CM, Kotronoulas G, Hesketh KR. Associations of screen time, sedentary time and physical activity with sleep in under 5s: A systematic review and meta-analysis. Sleep Medicine Reviews. 2020;49:N.PAG-N.PAG.

414.	Jaskova P, Palarea-Albaladejo J, Gaba A, Dumuid D, Pedisic Z, Pelclova J, et al. Compositional functional regression and isotemporal substitution analysis: Methods and application in time-use epidemiology. Statistical Methods in Medical Research. 2023;32(10):2064-80.

415.	Jauregui A, Argumedo G, Medina C, Bonvecchio-Arenas A, Romero-Martinez M, Okely AD. Factors associated with changes in movement behaviors in toddlers and preschoolers during the COVID-19 pandemic: A national cross-sectional study in Mexico. Preventive Medicine Reports. 2021;24((Jauregui, Argumedo, Medina, Bonvecchio-Arenas) Health and Nutrition Research Center, National Institute of Public Health, Cuernavaca, Mexico(Romero-Martinez) Population Health Research Center, National Institute of Public Health, Cuernavaca, Mexico(Okely):101552.

416.	Jauregui A, Salvo D, Aguilar-Farias N, Okely A. Movement behaviors during COVID-19 among Latin American/Latino toddlers and pre-schoolers in Chile, Mexico and the US. Scientific reports. 2022;12(1):19156.

417.	Jayamaha AR, Jones AV, Katagira W, Girase B, Yusuf ZK, Pina I, et al. Systematic Review of Physical Activity, Sedentary Behaviour and Sleep Among Adults Living with Chronic Respiratory Disease in Low-and Middle-Income Countries. International Journal of COPD. 2022;17:821-54.

418.	Ji Y, Atakan MM, Yan X, Wu J, Kuang J, Peng L. Reallocating just 10 min to moderate-to-vigorous physical activity from other components of 24-hour movement behaviors improves cardiovascular health in adults. BMC public health. 2024;24(1):1768.

419.	Jiang S, Ng JYY, Chong KH, Peng B, Ha AS. Effects of eHealth Interventions on 24-Hour Movement Behaviors Among Preschoolers: Systematic Review and Meta-Analysis. Journal of Medical Internet Research. 2024;26(1):e52905.

420.	Jiang Y, Lu Y, Cui J, Chu A. 24-hour movement behaviors time allocation and depression among Chinese community-dwelling older adults: A latent profile analysis. Geriatric Nursing. 2024;58:382-7.

421.	Jiao Y, Liu Z, Zhang XD. Relationship between daily physical activity and sleep quality among college students. Chinese Journal of School Health. 2021;42(7):1047-51.

422.	Jin L, Wuyun G, Deli G. Correlation between 24-hour time allocation patterns and physical fitness of Mongolian junior school students. Chinese Journal of School Health. 2023;44(12):1853-7.

423.	Joan C, Koh D, Wong JE, Khouw I, Poh BK. Association between adherence to 24-h movement guidelines and sociodemographic factors among Malaysian preschoolers: Findings from SEANUTS II Malaysia. American journal of human biology : the official journal of the Human Biology Council. 2024((Joan, Wong, Poh) Faculty of Health Sciences, Universiti Kebangsaan Malaysia, Kuala Lumpur, Malaysia(Koh) Faculty of Education, Universiti Kebangsaan Malaysia, Malaysia(Khouw) R&D, Expert Team Nutrition, FrieslandCampina, Amersfoort, Netherlands):e24076.

424.	Johansson MS, Søgaard K, Prescott E, Marott JL, Schnohr P, Holtermann A, et al. Can we walk away from cardiovascular disease risk or do we have to 'huff and puff'? A cross-sectional compositional accelerometer data analysis among adults and older adults in the Copenhagen City Heart Study. International Journal of Behavioral Nutrition and Physical Activity. 2020;17(1).

425.	Johansson PJ, Crowley P, Axelsson J, Franklin K, Garde AH, Hettiarachchi P, et al. Development and performance of a sleep estimation algorithm using a single accelerometer placed on the thigh: An evaluation against polysomnography. Journal of Sleep Research. 2023;32(2):1-10.

426.	Johnson C, Chaput JP, Diasparra M, Richard C, Dubois L. Influence of physical activity, screen time and sleep on inmates’ body weight during incarceration in Canadian federal penitentiaries: a retrospective cohort study. Canadian Journal of Public Health. 2019;110(2):198-209.

427.	Julian G, Valérie J, David T, Katharina M, Maria SA, Daniel W. Childhood obesity prevention: What can be achieved? Current Opinion in Clinical Nutrition and Metabolic Care. 2022;25(3):223-31.

428.	Julian V, Haschke F, Fearnbach N, Gomahr J, Pixner T, Furthner D, et al. Effects of Movement Behaviors on Overall Health and Appetite Control: Current Evidence and Perspectives in Children and Adolescents. Current obesity reports. 2022;11(1):10-22.

429.	Kaizu Y, Kasuga T, Takahashi Y, Otani T, Miyata K. Sleep Should Be Focused on When Analyzing Physical Activity in Hospitalized Older Adults after Trunk and Lower Extremity Fractures—A Pilot Study. Healthcare (Switzerland). 2022;10(8).

430.	Kandola AA, del Pozo Cruz B, Osborn DPJ, Stubbs B, Choi KW, Hayes JF. Impact of replacing sedentary behaviour with other movement behaviours on depression and anxiety symptoms: a prospective cohort study in the UK Biobank. BMC Medicine. 2021;19(1).

431.	Kang SJ, Leroux A, Guo W, Dey D, Strippoli MPF, Di J, et al. Integrative Modeling of Accelerometry-Derived Sleep, Physical Activity, and Circadian Rhythm Domains with Current or Remitted Major Depression. JAMA Psychiatry. 2024;81(9):911-8.

432.	Kariippanon KE, Hongyan G, Aguilar-Farias N, El Hamdouchi A, Lubree H, Draper CE. Voices of Children on Movement Behaviours in the Early Years: Reflections from Six Diverse Country Settings. International Journal of Qualitative Methods. 2023;22.

433.	Kasai A, Shikano A, Tanaka R, Yoshinaga M, Noi S. School-aged children’s movement behaviours and subjective health complaints in Japan: a cross-sectional study during COVID-19 pandemic-related school closures and after school reopenings. BMC Public Health. 2024;24(1).

434.	Kastelic K, Löfler S, Matko Š, Šarabon N. Validity of the German Version of Daily Activity Behaviours Questionnaire Among Older Adults. Journal of Aging & Physical Activity. 2023;31(6):1016-22.

435.	Kastelic K, Pedisic Z, Lipovac D, Kastelic N, Chen ST, Sarabon N. Associations of meeting 24-h movement guidelines with stress and self-rated health among adults: is meeting more guidelines associated with greater benefits? BMC public health. 2021;21(1):929.

436.	Kastelic K, Šarabon N. VALIDITY AND RELIABILITY OF THE DAILY ACTIVITY BEHAVIOURS QUESTIONNAIRE (DABQ) FOR THE ASSESSMENT OF 24-H MOVEMENT BEHAVIOURS AMONG ADOLESCENTS. Kinesiology. 2023;55(2):289-97.

437.	Kastelic K, Sarabon N, Burnard MD, Lipovac D, Pedisic Z. Association of meeting 24-hour movement guidelines with low back pain among adults. AIMS public health. 2023;10(4):964-79.

438.	Kastelic K, Sarabon N, Burnard MD, Pedisic Z. Validity and Reliability of the Daily Activity Behaviours Questionnaire (DABQ) for Assessment of Time Spent in Sleep, Sedentary Behaviour, and Physical Activity. International Journal of Environmental Research and Public Health. 2022;19(9):5362.

439.	Kastelic K, Sarabon N, Stanford T, Dumuid D, Pedisic Z. Are reallocations of time between physical activity, sedentary behaviour and sleep associated with low back pain? A compositional data analysis. BMJ Open Sport and Exercise Medicine. 2023;9(4):e001701.

440.	Katzmarzyk PT, Staiano AE. Relationship Between Meeting 24-Hour Movement Guidelines and Cardiometabolic Risk Factors in Children. Journal of physical activity & health. 2017;14(10):779-84.

441.	Kauffeldt KD, Varkul O, Latimer-Cheung AE, Faulkner G, Brouwers MC, Chulak-Bozzer T, et al. Awareness and knowledge of the Canadian 24-Hour Movement Guidelines for Adults among adults living in Canada. Applied Physiology, Nutrition and Metabolism. 2024;49(3):405-10.

442.	Keadle SK, Kravitz ES, Matthews CE, Tseng M, Carroll RJ. Development and Testing of an Integrated Score for Physical Behaviors. Medicine & Science in Sports & Exercise. 2019;51(8):1759-66.

443.	Keating SE, Chawla Y, De A, George ES. Lifestyle intervention for metabolic dysfunction-associated fatty liver disease: a 24-h integrated behavior perspective. Hepatology International. 2024.

444.	Khan A, Ahmed KR, Lee EY. Adherence to 24-hour movement guidelines and their association with depressive symptoms in adolescents: Evidence from Bangladesh. Sports Medicine and Health Science. 2024;6(1):76-81.

445.	Khan A, Lee E-Y, Tremblay MS. Meeting 24-h movement guidelines and associations with health related quality of life of Australian adolescents. Journal of science and medicine in sport. 2021;24(5):468-73.

446.	Kidokoro T, Tomkinson GR, Lang JJ, Suzuki K. Physical fitness before and during the COVID-19 pandemic: Results of annual national physical fitness surveillance among 16,647,699 Japanese children and adolescents between 2013 and 2021. Journal of Sport and Health Science. 2023;12(2):246-54.

447.	Kim H, Ma J, Harada K, Lee S, Gu Y. Associations between Adherence to Combinations of 24-h Movement Guidelines and Overweight and Obesity in Japanese Preschool Children. International journal of environmental research and public health. 2020;17(24).

448.	Kim TV, Pham TND, Nguyen CLD, Nguyen TTD, Okely AD, Tang HK. Prevalence of Physical Activity, Screen Time, and Sleep, and Associations with Adiposity and Motor Development among Preschool-Age Children in Vietnam: The SUNRISE Vietnam Pilot Study. Indian Journal of Pediatrics. 2022;89(2):148-53.

449.	Kim Y, Burns RD, Lee DC, Welk GJ. Associations of movement behaviors and body mass index: comparison between a report-based and monitor-based method using Compositional Data Analysis. International Journal of Obesity. 2021;45(1):266-75.

450.	Kim Y, Wijndaele K, Sharp SJ, Strain T, Pearce M, White T, et al. Specific physical activities, sedentary behaviours and sleep as long-term predictors of accelerometer-measured physical activity in 91,648 adults: A prospective cohort study. International Journal of Behavioral Nutrition and Physical Activity. 2019;16(1).

451.	Kim YB, McCurdy AP, Lamboglia CG, Hakimi S, Kuzik N, Lee EY, et al. Ambient air pollution and movement behaviours: A scoping review. Health and Place. 2021;72.

452.	Kindratt TB, Moza J, Rethorst CD, Liao Y. How do People Spend their Day? Sociodemographic Disparities in 24-hour Movement Guideline Adherence among US Adults Using 2017–2020 NHANES Data. Journal of Racial & Ethnic Health Disparities. 2024;11(4):2346-54.

453.	Kitano N, Kai Y, Jindo T, Tsunoda K, Arao T. Compositional data analysis of 24-hour movement behaviors and mental health in workers. Preventive Medicine Reports. 2020;20((Kitano, Kai, Jindo, Tsunoda, Arao) Physical Fitness Research Institute, Meiji Yasuda Life Foundation of Health and Welfare, 150 Tobuki, Hachioji, Tokyo 192-0001, Japan(Tsunoda) Faculty of Social Welfare, Yamaguchi Prefectural University, 3-2-1 Sakurabata):101213.

454.	Knell G, Li Q, Morales-Marroquin E, Drope J, Gabriel KP, Shuval K. Physical activity, sleep, and sedentary behavior among successful long-term weight loss maintainers: Findings from a U.S. national study. International Journal of Environmental Research and Public Health. 2021;18(11).

455.	Koivukoski H, Hasanen E, Tolvanen A, Chua T, Chia M, Vehmas H, et al. Meeting the WHO 24-h guidelines among 2–6-year-old children by family socioeconomic status before and during the COVID-19 pandemic: a repeated cross-sectional study. Journal of Activity, Sedentary and Sleep Behaviors. 2023;2(1).

456.	Kolk MZH, Frodi DM, Andersen TO, Langford J, Diederichsen SZ, Svendsen JH, et al. Accelerometer-assessed physical behavior and the association with clinical outcomes in implantable cardioverter-defibrillator recipients: A systematic review. Cardiovascular digital health journal. 2022;3(1):46-55.

457.	Kong C, Chen A, Ludyga S, Herold F, Healy S, Zhao M, et al. Associations between meeting 24-hour movement guidelines and quality of life among children and adolescents with autism spectrum disorder. Journal of sport and health science. 2023;12(1):73-86.

458.	Kontostoli E, Jones AP, Pearson N, Foley L, Biddle SJH, Atkin AJ. The association of contemporary screen behaviours with physical activity, sedentary behaviour and sleep in adolescents: A cross-sectional analysis of the millennium cohort study. International Journal of Behavioral Medicine. 2022.

459.	Koohsari MJ, Yasunaga A, McCormack GR, Shibata A, Ishii K, Liao Y, et al. Sedentary behaviour and sleep quality. Scientific Reports. 2023;13(1).

460.	Kostick MD, Zhu X. Movement Behaviors and Mental Health of Catholic Priests in the Eastern United States. Journal of Religion & Health. 2024;63(3):1867-79.

461.	Kracht CL, Drews KL, Flanagan EW, Keadle SK, Gallagher D, Van Horn L, et al. Maternal 24-h movement patterns across pregnancy and postpartum: The LIFE-Moms consortium. Preventive Medicine Reports. 2024;42((Kracht, Drews, Flanagan, Redman) Pennington Biomedical Research Center, Baton Rouge, LA, United States(Kracht) University of Kansas, Medical Center, Kansas City, KS 66160, United States(Keadle, Phelan) California Polytechnic State University, San Luis Ob):102740.

462.	Kracht CL, Katzmarzyk PT, Champagne CM, Broyles ST, Hsia DS, Newton Jr RL, et al. Association between Sleep, Sedentary Time, Physical Activity, and Adiposity in Adolescents: A Prospective Observational Study. Medicine & Science in Sports & Exercise. 2023;55(1):110-8.

463.	Kracht CL, Katzmarzyk PT, Staiano AE. Household chaos, family routines, and young child movement behaviors in the U.S. during the COVID-19 outbreak: a cross-sectional study. BMC Public Health. 2021;21(1).

464.	Kracht CL, Redman LM, Casey PH, Krukowski RA, Andres A. Association between Home Environment in Infancy and Child Movement Behaviors. Childhood Obesity. 2021;17(2):100-9.

465.	Kracht CL, Webster EK, Staiano AE. Sociodemographic Differences in Young Children Meeting 24-Hour Movement Guidelines. Journal of physical activity & health. 2019;16(10):908-15.

466.	Kracht CL, Webster EK, Staiano AE. Relationship between the 24-Hour Movement Guidelines and fundamental motor skills in preschoolers. Journal of science and medicine in sport. 2020;23(12):1185-90.

467.	Krietsch KN, Duraccio KM, Zhang N, Saelens BE, Howarth T, Combs A, et al. Earlier bedtimes and more sleep displace sedentary behavior but not moderate-to-vigorous physical activity in adolescents. Sleep Health. 2022;8(3):270-6.

468.	Kuzik N, Naylor P-J, Spence JC, Carson V. Parent–child Movement Behaviors and Bluetooth Proximity in Preschool-aged Children. Measurement in Physical Education & Exercise Science. 2021;25(4):353-64.

469.	Kuzik N, Naylor PJ, Spence JC, Carson V. Movement behaviours and physical, cognitive, and social-emotional development in preschool-aged children: Cross-sectional associations using compositional analyses. PLoS ONE. 2020;15(8 August):e0237945.

470.	Kuzik N, Poitras VJ, Tremblay MS, Lee EY, Hunter S, Carson V. Systematic review of the relationships between combinations of movement behaviours and health indicators in the early years (0-4 years). BMC Public Health. 2017;17.

471.	Kuzik N, Spence JC, Arkko K, Blye C-J, Davie J, Duddridge R, et al. Associations between meeting the Canadian 24-hour movement guidelines and physical, cognitive, social-emotional, and overall development in early childhood. Journal of Activity, Sedentary and Sleep Behaviors. 2022;1(1).

472.	Kyan A, Takakura M, Miyagi M. Associations between 24-h movement behaviors and self-rated health: a representative sample of school-aged children and adolescents in Okinawa, Japan. Public Health (Elsevier). 2022;213:117-23.

473.	Lai TF, Chang CC, Hsueh MC, Koohsari MJ, Shibata A, Liao Y, et al. Association of 24-Hour movement behavior and cognitive function in older Taiwanese adults. Geriatric nursing (New York, NY). 2024;59((Lai) Health Convergence Medicine Laboratory, Biomedical Research Institute, Pusan National University Hospital, Busan, Republic of Korea; Graduate Institute of Sport, Leisure and Hospitality Management, National Taiwan Normal University, Taipei, Taiwan(C):60-6.

474.	Lamboglia CG, McCurdy AP, Kim YB, Lindeman C, Mangan AJ, Sivak A, et al. Investigation of movement-related behaviors and energy compensation in people living with liver disease: A scoping review. Journal of Sports Sciences. 2022;40(12):1299-307.

475.	Larisch LM, Bojsen-Møller E, Nooijen CFJ, Blom V, Ekblom M, Ekblom Ö, et al. Effects of two randomized and controlled multi-component interventions focusing on 24-hour movement behavior among office workers: A compositional data analysis. International Journal of Environmental Research and Public Health. 2021;18(8).

476.	Larisch LM, Kallings LV, Hagstromer M, Desai M, Rosen PV, Blom V. Associations between 24 h movement behavior and mental health in office workers. International Journal of Environmental Research and Public Health. 2020;17(17):1-20.

477.	Larsen SC, O'Driscoll R, Horgan G, Mikkelsen MLK, Specht IO, Rohde JF, et al. Substituting sedentary time with sleep or physical activity and subsequent weight-loss maintenance. Obesity. 2023;31(2):515-24.

478.	Latimer-Cheung AE, Copeland JL, Fowles J, Zehr L, Duggan M, Tremblay MS. The Canadian 24-Hour Movement Guidelines for Children and Youth: Implications for practitioners, professionals, and organizations. Applied Physiology, Nutrition & Metabolism. 2016;41:S328-S35.

479.	Lau PWC, Song H, Song D, Wang JJ, Zhen S, Shi L, et al. 24‐Hour movement behaviors and executive functions in preschoolers: A compositional and isotemporal reallocation analysis. Child Development. 2024;95(2):e110-e21.

480.	Laxton P, Healy S, Brewer B, Patterson F. Prevalence of current smoking and association with meeting 24-h movement guidelines: Results from a national convenience sample of autistic adults. Autism: The International Journal of Research & Practice. 2024;28(2):474-83.

481.	Le F, Yap Y, Tung NYC, Bei B, Wiley JF. The Associations Between Daily Activities and Affect: a Compositional Isotemporal Substitution Analysis. International Journal of Behavioral Medicine. 2022;29(4):456-68.

482.	Lee E-Y, Kim Y-B, Goo S, Oyama O, Lee J, Kim G, et al. Physical activity in the era of climate change and COVID-19 pandemic: Results from the South Korea's 2022 Report Card on physical activity for children and adolescents. Journal of exercise science and fitness. 2023;21(1):26-33.

483.	Lee E-Y, Park S, Kim Y-B, Lee M, Lim H, Ross-White A, et al. Exploring the Interplay Between Climate Change, 24-Hour Movement Behavior, and Health: A Systematic Review. Journal of physical activity & health. 2024(101189457):1-19.

484.	Lee EY, Carson V, Jeon JY, Spence JC, Tremblay MS. Levels and correlates of 24-hour movement behaviors among South Koreans: Results from the Korea National Health and Nutrition Examination Surveys, 2014 and 2015. Journal of Sport and Health Science. 2019;8(4):376-85.

485.	Lee EY, Hesketh KD, Hunter S, Kuzik N, Rhodes RE, Rinaldi CM, et al. Meeting new <i>Canadian</i> <i>24</i>-<i>Hour</i> <i>Movement</i> <i>Guidelines</i> <i>for the Early Years</i> and associations with adiposity among toddlers living in Edmonton, Canada. BMC PUBLIC HEALTH. 2017;17.

486.	Lee EY, Khan A, Uddin R, Lim E, George L. Six-year trends and intersectional correlates of meeting 24-Hour Movement Guidelines among South Korean adolescents: Korea Youth Risk Behavior Surveys, 2013-2018. Journal of sport and health science. 2023;12(2):255-65.

487.	Lee EY, Spence JC, Tremblay MS, Carson V. Meeting <i>24</i>-<i>Hour Movement Guidelines for Children and Youth</i> and associations with psychological well-being among South Korean adolescents. MENTAL HEALTH AND PHYSICAL ACTIVITY. 2018;14:66-73.

488.	Lee J, Walker ME, Matthews KA, Kuller LH, Ranjit N, Gabriel KP. Associations of physical activity and sleep with cardiometabolic risk in older women. Preventive Medicine Reports. 2020;18.

489.	Lee ST, Wong JE, Chan GKL, Poh BK. Association between compliance with movement behavior guidelines and obesity among malaysian preschoolers. International Journal of Environmental Research and Public Health. 2021;18(9):4611.

490.	Leech RM, Chappel SE, Ridgers ND, Eicher-Miller HA, Maddison R, McNaughton SA. Analytic Methods for Understanding the Temporal Patterning of Dietary and 24-H Movement Behaviors: A Scoping Review. Advances in nutrition (Bethesda, Md). 2024;15(8):100275.

491.	Lemes VB, Gaya ACA, Gaya AR. Comparison of 24-h movement behavior, health-related quality of life, and waist to height ratio between adolescents with healthy body mass index (BMI) and adolescents with BMI in the cardiometabolic risk zone. Sport Sciences for Health. 2023;19(1):231-9.

492.	Lemos L, Clark C, Brand C, Pessoa ML, Gaya A, Mota J, et al. 24‐hour movement behaviors and fitness in preschoolers: A compositional and isotemporal reallocation analysis. Scandinavian Journal of Medicine & Science in Sports. 2021;31(6):1371-9.

493.	Leppanen MH, Haapala EA, Vaisto J, Ekelund U, Brage S, Kilpelainen TO, et al. Longitudinal and cross-sectional associations of adherence to 24-hour movement guidelines with cardiometabolic risk. Scandinavian journal of medicine & science in sports. 2022;32(1):255-66.

494.	Leppänen MH, Ray C, Wennman H, Alexandrou C, Sääksjärvi K, Koivusilta L, et al. Compliance with the 24-h movement guidelines and the relationship with anthropometry in Finnish preschoolers: The DAGIS study. BMC Public Health. 2019;19(1).

495.	Lettink A, Altenburg TM, Arts J, van Hees VT, Chinapaw MJM. Systematic review of accelerometer-based methods for 24-h physical behavior assessment in young children (0–5 years old). International Journal of Behavioral Nutrition & Physical Activity. 2022;19(1):1-63.

496.	Lewthwaite H, Olds T, Williams MT, Effing TW, Dumuid D. Use of time in chronic obstructive pulmonary disease: Longitudinal associations with symptoms and quality of life using a compositional analysis approach. PloS one. 2019;14(3):e0214058.

497.	Li C, Haegele JA, Sun F, Alves MLT, Ang SHC, Lee J, et al. Meeting the 24-h movement guidelines and health-related outcomes among youth with autism spectrum disorder: a seven-country observational study. Child & Adolescent Psychiatry & Mental Health. 2022;16(1):1-8.

498.	Li C-R, Liu Y-P, Pan J-Y. Circadian rhythm of rest-activity and sleep pattern in remitted patients with bipolar I disorder: An actigraphy study. Chinese Mental Health Journal. 2016;30(3):161-6.

499.	Li D, Chua T, Chen M, Li X, Chia M. Effects of replacing sedentary time with alterations in physical activity or sleep on mood states in Chinese young adults during the pandemic. BMC public health. 2024;24(1):2184.

500.	Li F, Yin L, Luo W, Gao Z, Ryu S, Sun M, et al. Isotemporal substitution effect of 24-hour movement behavior on the mental health of Chinese preschool children. Frontiers in public health. 2024;12((Li, Luo) School of Physical Education, Hunan First Normal University, Changsha, China(Yin) College of Physical Education, Hunan Normal University, Changsha, China(Gao) Department of Kinesiology, Recreation, Sport Studies, University of Tennessee, Knoxvil):1288262.

501.	Li F, Yin L, Sun M, Gao Z. Examining Relationships among Chinese Preschool Children’s Meeting 24-Hour Movement Guidelines and Fundamental Movement Skills. Journal of Clinical Medicine. 2022;11(19).

502.	Li H, Zhang T, Gao Y. A scientific 24-hour movement behavior guideline for the implementation of the "double reduction" policy. Chinese Journal of School Health. 2023;44(1):6-10.

503.	Li MH, Sit CHP, Wong SHS, Wing YK, Ng CK, Sum RKW. Promoting physical activity and health in Hong Kong primary school children through a blended physical literacy intervention: protocol and baseline characteristics of the “Stand+Move” randomized controlled trial. Trials. 2021;22(1).

504.	Li N, Wang N, Lin S, Yuan Y, Huang F, Zhu P. A latent profile analysis of rest–activity behavior patterns among community-dwelling older adults and its relationship with intrinsic capacity. Scientific Reports. 2024;14(1).

505.	Liang HU, Weizhong D, Shanshan XU. Associations Between 24-hour Movement Behavior, Cognitive Function and Academic Performance among Children and Adolescents: Review and Prospects. (English). Journal of Beijing Sport University. 2024;47(3):40-55.

506.	Liang K, Chen S, Chi X. Differential Associations Between Meeting 24-Hour Movement Guidelines With Mental Wellbeing and Mental Illness Among Chinese Adolescents. The Journal of adolescent health : official publication of the Society for Adolescent Medicine. 2023;72(5):658-66.

507.	Liang K, de Lucena Martins CM, Chen S-T, Clark CCT, Duncan MJ, Bu H, et al. Sleep as a Priority: 24-Hour Movement Guidelines and Mental Health of Chinese College Students during the COVID-19 Pandemic. Healthcare (Basel, Switzerland). 2021;9(9).

508.	Liang W, Wang Y, Huang Q, Shang B, Su N, Zhou L, et al. Adherence to 24-Hour Movement Guidelines Among Chinese Older Adults: Prevalence, Correlates, and Associations With Physical and Mental Health Outcomes. JMIR public health and surveillance. 2024;10((Liang, Su) School of Physical Education, Shenzhen University, Shenzhen, China(Wang, Baker, Duan) Department of Sport, Physical Education and Health, Hong Kong Baptist University, Hong Kong, China(Huang) Fitness and Health Lab, Hubei Institute of Sport Sc):e46072.

509.	Liang W, Wang Y, Su N, Song H, Rhodes RE, Wang X, et al. Associations of Reallocating Sedentary Time to Physical Activity and Sleep with Physical and Mental Health of Older Adults. Medicine and science in sports and exercise. 2024((Liang, Su) School of Physical Education, Shenzhen University, Shenzhen, China(Wang, Song, Wang, Baker, Duan) Department of Sport, Physical Education and Health, Hong Kong Baptist University, Hong Kong, China(Rhodes) School of Exercise Science, Physical a).

510.	Liangruenrom N, Craike M, Dumuid D, Biddle SJH, Tudor-Locke C, Ainsworth B, et al. Standardised criteria for classifying the International Classification of Activities for Time-use Statistics (ICATUS) activity groups into sleep, sedentary behaviour, and physical activity. International Journal of Behavioral Nutrition & Physical Activity. 2019;16(1):N.PAG-N.PAG.

511.	Liangruenrom N, Dumuid D, Craike M, Biddle SJH, Pedisic Z. Trends and correlates of meeting 24-hour movement guidelines: a 15-year study among 167,577 Thai adults. International Journal of Behavioral Nutrition & Physical Activity. 2020;17(1):N.PAG-N.PAG.

512.	Liangruenrom N, Dumuid D, Pedisic Z. Physical activity, sedentary behaviour, and sleep in the Thai population: A compositional data analysis including 135,824 participants from two national time-use surveys. PLoS ONE. 2023;18(1 January):e0280957.

513.	Liao Y, Brothers RM, Brown KK, Lee RE. A biobehavioral observational study to understand the multilevel determinants of cardiovascular health in Black women: the BLOOM Study protocol. BMC Women's Health. 2024;24(1).

514.	Lien A, Sampasa-Kanyinga H, Colman I, Hamilton HA, Chaput JP. Adherence to 24-hour movement guidelines and academic performance in adolescents. Public Health (Elsevier). 2020;183:8-14.

515.	Lien A, Sampasa-Kanyinga H, Patte KA, Leatherdale ST, Chaput J-P. Sociodemographic and mental health characteristics associated with changes in movement behaviours due to the COVID-19 pandemic in adolescents. Journal of Activity, Sedentary and Sleep Behaviors. 2022;1(1).

516.	Lim R, Dumuid D, Parfitt G, Stanford T, Post D, Bilton R, et al. Using wrist-worn accelerometers to identify the impact of medicines with anticholinergic or sedative properties on sedentary time: A 12-month prospective analysis. Maturitas. 2023;172((Lim, Bilton, Kalisch Ellett, Pratt, Roughead) Quality Use of Medicines and Pharmacy Research Centre, UniSA Clinical and Health Sciences, University of South Australia, Adelaide 5000, Australia(Dumuid, Parfitt, Stanford, Post) Alliance for Research in Exe):9-14.

517.	Lin L, Liang W, Wang R, Rhodes RE, Liu H. Association of 24-hour movement guideline adherence, mental health and quality of life in young adults: the role of e-Health literacy. Frontiers in Public Health. 2024;12.

518.	Lin Y, Tremblay MS, Katzmarzyk PT, Fogelholm M, Hu G, Lambert EV, et al. Temporal and bi-directional associations between sleep duration and physical activity/sedentary time in children: An international comparison. Preventive Medicine. 2018;111((Lin) Queen's University, Kingston, Canada(Tremblay, Chaput) Children's Hospital of Eastern Ontario Research Institute, Ottawa, Canada(Katzmarzyk, Hu, Tudor-Locke) Pennington Biomedical Research Center, Baton Rouge, United States(Fogelholm) University of):436-41.

519.	Lin Z, Cheng J, Tan J, Huang B, Huang Z, Xu H, et al. Mediating effect of 24-hour movement behavior in the association between smartphone addiction and anxiety among col-lege students. Chinese Journal of School Health. 2023;44(1):32-5and9.

520.	Lin Z, Zhu S, Cheng J, Lin Q, Lawrence WR, Zhang W, et al. The mediating effect of engagement in physical activity over a 24-hour period on chronic disease and depression: Using compositional mediation model. Journal of Affective Disorders. 2022;299:264-72.

521.	Lithopoulos A, Dacin PA, Tremblay MS, Latimer-Cheung AE. Exploring determinants of brand extension attitude to promote optimal levels of movement among children and youth. Journal of Social Marketing. 2021;11(4):453-68.

522.	Liu B-P, Jia C-X, Li S-X. The Association of Soft Drink Consumption and the 24-Hour Movement Guidelines with Suicidality among Adolescents of the United States. Nutrients. 2022;14(9):1870-.

523.	Liu F, Kong B, Zhang Z, Chen L, Li Y, Xiong J, et al. Associations between adherence to 24-Hour Movement Guidelines with continuous metabolic syndrome score among Chinese children and adolescents. Public Health. 2024;236:274-80.

524.	Liu S, Yu Q, Hossain MM, Doig S, Bao R, Zhao Y, et al. Meeting 24-h Movement Guidelines is Related to Better Academic Achievement: Findings from the YRBS 2019 Cycle. International Journal of Mental Health Promotion. 2022;24(1):13-24.

525.	Liu X, Herold F, Werneck AO, Chen Y, Liu Z, Taylor A, et al. Prevalence of 24-hour movement behavior guidelines among youth with prescribed eyeglasses/contact lenses and anxiety. Mental Health and Physical Activity. 2024;26:1-8.

526.	Liu XQ, Liu ML, Wu ZW, Liang JH. Secular trends in the prevalence of meeting 24-hour movement guidelines among U.S. adolescents: evidence from NHANES 2007-2016. Frontiers in public health. 2024;12((Liu) School of History, Qingdao University, Qingdao, China(Liu, Wu, Liang) Department of Maternal and Child Health, School of Public Health, Sun Yat-sen University, Guangzhou, China):1362718.

527.	Liu Y, Ge X, Wang Y, Qiao S, Cai Y. How race and socioeconomic status moderate the association between moderate-to-vigorous physical activity and depressive symptoms: a cross-sectional study with compositional data. British Journal of Sports Medicine. 2024.

528.	Liu Y, Liu SX, Sum RKW, Duncan MJ, Gu YD, Li MH. Associations between levels of physical literacy and adherence to the 24-h movement guidelines among university students: A cross-sectional study. Journal of Exercise Science and Fitness. 2024;22(3):221-6.

529.	Liu Y, Tan S, Zou G. Correlation analysis between daily behavior and physical health of middle school students in Tianjin. Chinese Journal of School Health. 2022;43(10):1556-60.

530.	Liu Z, Herold F, Healy S, Haegele J, Block ME, Ludyga S, et al. Understanding 24-hour movement guideline adherence and links to school achievement, social-behavioural problems, and emotional functioning among children and adolescents with learning disabilities. International Journal of Sport and Exercise Psychology. 2023.

531.	Liu Z, Herold F, Owen N, Huang Z, Kuang J, Kramer AF, et al. Associations of meeting 24-hour movement behavior guidelines with prescribed eyeglasses/contact lenses among children and adolescents. Complementary Therapies in Clinical Practice. 2024;55:N.PAG-N.PAG.

532.	Loh GY, Chua TBK, Hian Tan K, Loo BKG, Quah PL, Chia MYH. Adherence to the Singapore Integrated 24-Hour Activity Guidelines by Infants and Toddlers and Its Association With Well-Being. Journal of physical activity & health. 2024;21(2):171-80.

533.	Loo BKG, Okely AD, Pulungan A, Jalaludin MY. Asia-Pacific Consensus Statement on integrated 24-hour activity guidelines for children and adolescents. British journal of sports medicine. 2022;56(10):539-45.

534.	Loo BKG, Sirisena D, Muller-Riemenschneider F, Chia MYH, Tan B, Tan NC, et al. Consensus statement on Singapore integrated 24-hour activity guide for early childhood. Annals of the Academy of Medicine, Singapore. 2023;52(6):310-20.

535.	Loo BKG, Tan B, Chia MYH, Chan PC, Sirisena D, Zainuddin MA, et al. Consensus statement on Singapore integrated 24-hour activity guide for children and adolescents. Annals of the Academy of Medicine, Singapore. 2022;51(5):292-9.

536.	Lopez-Gil JF, del Pozo-Cruz J, del Pozo Cruz B, Tarraga-Lopez PJ, Garcia-Hermoso A. Environmental tobacco smoke exposure and 24-h movement guidelines in Spanish young people. Translational Pediatrics. 2023;12(7):1327-35.

537.	López-Gil JF, Fabios E, Martín-Calvo N. Meeting the 24-h movement recommendations and its relationship with Mediterranean dietary patterns in early childhood: the SENDO project. European Journal of Pediatrics. 2024;183(5):2365-73.

538.	López-Gil JF, Firth J, García-Hermoso A. Is meeting with the 24-hr movement recommendations linked with suicidality? Results from a nationwide sample of 44,734 U.S. adolescents. Journal of Affective Disorders. 2024;349:617-24.

539.	López-Gil JF, García-Hermoso A, Cavero-Redondo I, Ortega FB, Gómez SF, Martínez-Vizcaíno V. Association between air pollution and 24-h movement behaviours in a representative sample of Spanish youth. Environmental Research. 2022;214.

540.	Lopez-Gil JF, Garcia-Hermoso A, Smith L, Gallego A, Victoria-Montesinos D, Ezzatvar Y, et al. A Cluster Randomized Controlled Trial of the Archena Infancia Saludable Project on 24-h Movement Behaviors and Adherence to the Mediterranean Diet among Schoolchildren: A Pilot Study Protocol. Children. 2023;10(4):738.

541.	López-Gil JF, Roman-Viñas B, Aznar S, Tremblay M. Meeting 24-h movement guidelines: Prevalence, correlates, and associations with socioemotional behavior in Spanish minors. Scandinavian Journal of Medicine and Science in Sports. 2022;32(5):881-91.

542.	Lopez-Gil JF, Tapia-Serrano MA, Sevil-Serrano J, Sanchez-Miguel PA, Garcia-Hermoso A. Are 24-hour movement recommendations associated with obesity-related indicators in the young population? A meta-analysis. Obesity (Silver Spring, Md). 2023;31(11):2727-39.

543.	López-Gil JF, Tremblay MS, Brazo-Sayavera J. Changes in healthy behaviors and meeting 24-h movement guidelines in spanish and brazilian preschoolers, children and adolescents during the covid-19 lockdown. Children. 2021;8(2).

544.	López-Gil JF, Tremblay MS, Tapia-Serrano MÁ, Tárraga-López PJ, Brazo-Sayavera J. Meeting 24 h Movement Guidelines and Health-Related Quality of Life in Youths during the COVID-19 Lockdown. Applied Sciences (Switzerland). 2022;12(16).

545.	Lostak J, Gaba A, Dygryn J, Balaz L, Stefelova N, Gallo J. Twenty-Four-Hour Movement Behaviors in Adults and Older Adults before Total Knee Arthroplasty: a Study Based on Compositional Data Analysis. Acta Chirurgiae Orthopaedicae et Traumatologiae Cechoslovaca. 2022;89(1):9-15.

546.	Lou JI, Wang Y, Li R, Zhou Y. Association of 24-hour movement behaviors with emotional and behavioral problems among left-behind children. Chinese Journal of School Health. 2024;45(1):72-6.

547.	Ltifi MA, Turki O, Ben-Bouzaiene G, Pagaduan JC, Okely A, Chelly MS. Exploring 24-Hour Movement Behaviors in Early Years: Findings From the SUNRISE Pilot Study in Tunisia. Pediatric exercise science. 2024((Ltifi, Turki, Ben-Bouzaiene, Chelly) Research Laboratory (LR23JS01) "Sport Performance, Health and Society, Higher Institute of Sport and Physical Education of Ksar Said, University of Manouba, Tunis, Tunisia(Pagaduan) Faculty of Physical Culture, Palack):1-8.

548.	Lu B, Huang Z, Lou JY, Li R, Zhou YL. Associations between 24-hour activity behaviours and emotional and behavioural problems of left-behind children: a component analysis of data from a cross-sectional study. BMJ OPEN. 2024;14(8):1-9.

549.	Lu S, Cheval B, Yu Q, Hossain MM, Chen S-T, Taylor A, et al. Associations of 24-Hour Movement Behavior with Depressive Symptoms and Anxiety in Children: Cross-Sectional Findings from a Chinese Sample. Healthcare (Basel, Switzerland). 2021;9(11).

550.	Lu Y, Wiltshire HD, Baker JS, Wang Q. Effects of Low-Volume High-Intensity Interval Exercise on 24 h Movement Behaviors in Inactive Female University Students. International Journal of Environmental Research and Public Health. 2022;19(12):7177.

551.	Lu Y, Zhao M. Adherence to 24-h movement guidelines and cognitive difficulties in adolescents. Complementary Therapies in Clinical Practice. 2023;51.

552.	Lu Z, Qu X, Chang J, Xu M, Song G, Wang X, et al. Reallocation of time between preschoolers’ 24-h movement behaviours and executive functions: A compositional data analysis. Journal of Sports Sciences. 2023;41(12):1187-95.

553.	Lund Rasmussen C, Gaba A, Stanford T, Dygryn J, Dumuid D, Janda D, et al. The Goldilocks Day for healthy adiposity measures among children and adolescents. Frontiers in public health. 2023;11((Lund Rasmussen, Gaba, Dygryn, Janda) Faculty of Physical Culture, Palacky University Olomouc, Czechia, Olomouc(Lund Rasmussen) Department of Public Health and Nursing, Norwegian University of Science and Technology, Trondheim, Norway(Lund Rasmussen) Scho):1158634.

554.	Luo L. 24-H movement behaviors and visual impairment among Chinese adolescents with and without obesity. Complementary Therapies in Clinical Practice. 2024;54:N.PAG-N.PAG.

555.	Luo L, Cao Y, Hu Y, Wen S, Tang K, Ding L, et al. The Associations between Meeting 24-Hour Movement Guidelines (24-HMG) and Self-Rated Physical and Mental Health in Older Adults-Cross Sectional Evidence from China. International Journal of Environmental Research and Public Health. 2022;19(20):13407.

556.	Luo L, Zeng X, Cao Y, Hu Y, Wen S, Tang K, et al. The Associations between Meeting 24-Hour Movement Guidelines (24-HMG) and Mental Health in Adolescents-Cross Sectional Evidence from China. International journal of environmental research and public health. 2023;20(4).

557.	Lyu Y, Cai L, Zeng X, Lai L, Tan W, Ma J, et al. Levels and correlates of 24-hour movement behaviors in Chinese children aged 6-13 years. Chinese Journal of School Health. 2019;40(12):1791-5.

558.	Lyu Z, Li T. Relationship between physical activity screen time and sleep duration and overweight and obesity among children and adolescents in Beijing-Tianjin-Hebei region. Chinese Journal of School Health. 2022;43(4):502-6.

559.	Ma C, Yan J, Hu H, Shi C, Li F, Zeng X. Associations between 24-h Movement Behavior and Internet Addiction in Adolescents: A Cross-Sectional Study. International Journal of Environmental Research and Public Health. 2022;19(24):16873.

560.	Maciel GP, da Costa BGG, Bezerra IN, Silva KS, Lopes IE, de Castro VHS, et al. Factors related to depressive symptoms during the second year of COVID-19 pandemic in Brazil: A cross-sectional study with adolescents. Journal of Pediatric Nursing. 2023;73:e534-e40.

561.	MacLeod L, Bouchard DR, Hebert JJ, Boudreau JG, Senechal M. Association Between a Comprehensive Movement Assessment and Metabolically Healthy Overweight Obese Adults. Scientific reports. 2020;10(1):1173.

562.	Maddren CI, Dhamrait G, Elliott K, Toledo-Vargas M, Gryech I, Okely AD. Associations between Postnatal Pollution Exposures, 24-h Movement Behaviours, and Motor Development Outcomes among Children (0-12 Years Old): A Systematic Review. Indoor Air. 2024;2024.

563.	Magee C, Caputi P, Iverson D. Lack of sleep could increase obesity in children and too much television could be partly to blame. Acta Paediatrica, International Journal of Paediatrics. 2014;103(1):e27-e31.

564.	Majed EZ, Janssen I, Levesque L. Meeting the 24-hour movement guidelines among adult immigrants in Canada. Canadian journal of public health = Revue canadienne de sante publique. 2024((Majed) School of Kinesiology and Health Studies, Queen's University, Kingston, ON, Canada. 18ezm@queensu.ca(Janssen, Levesque) School of Kinesiology and Health Studies, Queen's University, Kingston, ON, Canada(Janssen) Department of Public Health Science).

565.	Manifield J, Chaudhry Y, Singh SJ, Ward TJC, Whelan ME, Orme MW. Changes in physical activity, sedentary behaviour and sleep following pulmonary rehabilitation: a systematic review and network meta-analysis. European Respiratory Review. 2024;33(172).

566.	Manyanga T, Barnes JD, Chaput JP, Guerrero M, Katzmarzyk PT, Mire EF, et al. Body mass index and movement behaviors among schoolchildren from 13 countries across a continuum of human development indices: A multinational cross-sectional study. American journal of human biology : the official journal of the Human Biology Council. 2020;32(2):e23341.

567.	Manyanga T, Barnes JD, Chaput JP, Katzmarzyk PT, Prista A, Tremblay MS. Prevalence and correlates of adherence to movement guidelines among urban and rural children in Mozambique: A cross-sectional study. International Journal of Behavioral Nutrition and Physical Activity. 2019;16(1).

568.	Marciano L, Camerini AL. Recommendations on screen time, sleep and physical activity: associations with academic achievement in Swiss adolescents. Public Health. 2021;198:211-7.

569.	Marlow AL, King BR, Trost SG, Weaver N, Smart CE. Healthy weight and overweight adolescents with type 1 diabetes mellitus do not meet recommendations for daily physical activity and sleep. Diabetes Research and Clinical Practice. 2023;203.

570.	Marmol-Perez A, Migueles JH, Ubago-Guisado E, Gil-Cosano JJ, Rodriguez-Solana A, Redondo-TÉBar A, et al. Every Move Counts to Improve Bone Health at Clinical Sites in Young Pediatric Cancer Survivors: The iBoneFIT Project. Medicine & Science in Sports & Exercise. 2024;56(6):1085-93.

571.	Marmol-Perez A, Migueles JH, Ubago-Guisado E, Gil-Cosano JJ, Rodriguez-Solana A, Redondo-Tebar A, et al. Every Move Counts to Improve Bone Health at Clinical Sites in Young Pediatric Cancer Survivors: The iBoneFIT Project. Medicine and science in sports and exercise. 2024;56(6):1085-93.

572.	Marques A, Ramirez-Campillo R, Gouveia ÉR, Ferrari G, Tesler R, Marconcin P, et al. 24-h Movement Guidelines and Overweight and Obesity Indicators in Toddlers, Children and Adolescents: A Systematic Review and Meta-Analysis. Sports Medicine - Open. 2023;9(1).

573.	Marques PA, Voser RD, Valle MBD, Martins CMD, Gaya ACA, Mota J, et al. ASSOCIAÇÃO DO MOVIMENTO DE 24 HORAS COM INDICADORES DE SOBREPESO/OBESIDADE EM ESCOLARES. RBONE-REVISTA BRASILEIRA DE OBESIDADE NUTRICAO E EMAGRECIMENTO. 2024;18(112):164-73.

574.	Marshall ZA, Mackintosh KA, Gregory JW, McNarry MA. Using compositional analysis to explore the relationship between physical activity and cardiovascular health in children and adolescents with and without type 1 diabetes. Pediatric Diabetes. 2022;23(1):115-25.

575.	Marshall ZA, Mackintosh KA, McNarry MA. Investigating the influence of physical activity composition on arterial stiffness in youth. European Journal of Sport Science. 2023;23(4):617-24.

576.	Martinez SM, Tschann JM, McCulloch CE, Sites E, Butte NF, Gregorich SE, et al. Temporal associations between circadian sleep and activity patterns in Mexican American children. Sleep health. 2019;5(2):201-7.

577.	Martins C, Mota J, Goulart N, Silva M, Silva T, Carvalho F, et al. Compliance with the 24-h movement behaviors guidelines among urban and rural Brazilian preschoolers. American Journal of Human Biology. 2024;36(6).

578.	Martins C, Ribeiro Bandeira PF, Filho AS, Bezerra T, Clark C, Webster EK, et al. The combination of three movement behaviours is associated with object control skills, but not locomotor skills, in preschoolers. European Journal of Pediatrics. 2021;180(5):1505-12.

579.	Martins CML, Clark CCT, Bandeira PFR, Mota J, Duncan MJ. Association between compliance with the 24-hour movement guidelines and fundamental movement skills in preschoolers: A network perspective. International Journal of Environmental Research and Public Health. 2020;17(15):1-13.

580.	Martins J, Tapia-Serrano MÁ, Nogueira T, Sanchéz-Miguel PA. Compliance with the 24-h Movement Guidelines for Portuguese children: differences between boys and girls. Scientific Reports. 2024;14(1).

581.	Master L, Nye RT, Lee S, Nahmod NG, Mariani S, Hale L, et al. Bidirectional, Daily Temporal Associations between Sleep and Physical Activity in Adolescents. Scientific reports. 2019;9(1):7732.

582.	Materová E, Pelclová J, Gába A, Frömel K. Surveillance of physical activity and sedentary behaviour in czech children and adolescents: a scoping review of the literature from the past two decades. BMC Public Health. 2022;22(1).

583.	Matricciani L, Dumuid D, Paquet C, Fraysse F, Wang Y, Baur LA, et al. Sleep and cardiometabolic health in children and adults: examining sleep as a component of the 24-h day. Sleep Medicine. 2021;78:63-74.

584.	Matricciani L, Dumuid D, Stanford T, Maher C, Bennett P, Bobrovskaya L, et al. Time use and dimensions of healthy sleep: A cross-sectional study of Australian children and adults. Sleep health. 2024;10(3):348-55.

585.	Matthews CE, Berrigan D, Fischer B, Gomersall SR, Hillreiner A, Kim Y, et al. Use of previous-day recalls of physical activity and sedentary behavior in epidemiologic studies: results from four instruments. BMC Public Health. 2019;19(1):N.PAG-N.PAG.

586.	Matthews CE, Keadle SK, Saint-Maurice PF, Moore SC, Willis EA, Sampson JN, et al. Use of Time and Energy on Exercise, Prolonged TV Viewing, and Work Days. American Journal of Preventive Medicine. 2018;55(3):e61-e9.

587.	McDevitt B, Moore L, Akhtar N, Connolly J, Doherty R, Scott W. Validity of a novel research-grade physical activity and sleep monitor for continuous remote patient monitoring. Sensors. 2021;21(6):1-15.

588.	McGee M, Unger S, Hamilton J, Birken CS, Pausova Z, Vanderloo LM, et al. Lean mass accretion in children born very low birth weight is significantly associated with estimated changes from sedentary time to light physical activity. Pediatric Obesity. 2020;15(5).

589.	McGowan AL, Gerde HK, Pfeiffer KA, Pontifex MB. Meeting 24-hour movement behavior guidelines in young children: Improved quantity estimation and self-regulation. Early Education and Development. 2023;34(3):762-89.

590.	McGregor DE, Carson V, Palarea-Albaladejo J, Dall PM, Tremblay MS, Chastin SFM. Compositional analysis of the associations between 24-h movement behaviours and health indicators among adults and older adults from the Canadian health measure survey. International Journal of Environmental Research and Public Health. 2018;15(8):1779.

591.	McGregor DE, Palarea-Albaladejo J, Dall PM, Del Pozo Cruz B, Chastin SFM. Compositional analysis of the association between mortality and 24-hour movement behaviour from NHANES. European journal of preventive cardiology. 2021;28(7):791-8.

592.	McGregor DE, Palarea-Albaladejo J, Dall PM, Hron K, Chastin SFM. Cox regression survival analysis with compositional covariates: Application to modelling mortality risk from 24-h physical activity patterns. Statistical Methods in Medical Research. 2020;29(5):1447-65.

593.	McNarry MA, Stevens D, Stone M, Roberts S, Hall S, Mackintosh KA. Physical activity, sedentary time and sleep in cystic fibrosis youth: A bidirectional relationship? Pediatric Pulmonology. 2021;56(2):450-6.

594.	McNeill J, Howard SJ, Vella SA, Cliff DP. Compliance with the 24-Hour movement guidelines for the early years: Cross-sectional and longitudinal associations with executive function and psychosocial health in preschool children. Journal of science and medicine in sport. 2020;23(9):846-53.

595.	Medina C, Jáuregui A, Blas N, Campos I, Macías N, López-Ridaura R, et al. Promotion of physical activity in schoolchildren and adolescents. Salud Publica de Mexico. 2024;66(4):437-47.

596.	Medina C, Jáuregui A, Hernández C, González C, Olvera AG, Blas N, et al. Prevalence of movement behaviors in Mexico. Salud Publica de Mexico. 2023;65.

597.	Mei S, Lv J, Ren H, Guo X, Meng C, Fei J, et al. Lifestyle Behaviors and Depressive Symptoms in Chinese Adolescents Using Regression and fsQCA Models. Frontiers in Public Health. 2022;10.

598.	Meister DJ, Kehler DS, Bouchard DR, Thomson AM, Sénéchal M. The association of adherence to 24-hour movement guidelines with frailty and mortality: cross-sectional and longitudinal analyses of NHANES data. Journal of Activity, Sedentary and Sleep Behaviors. 2024;3(1).

599.	Mellow ML, Crozier AJ, Dumuid D, Wade AT, Goldsworthy MR, Dorrian J, et al. How are combinations of physical activity, sedentary behaviour and sleep related to cognitive function in older adults? A systematic review. Experimental gerontology. 2022;159(epq, 0047061):111698.

600.	Mellow ML, Dumuid D, Olds T, Stanford T, Dorrian J, Wade AT, et al. Cross-sectional associations between 24-hour time-use composition, grey matter volume and cognitive function in healthy older adults. International Journal of Behavioral Nutrition & Physical Activity. 2024;21(1):1-17.

601.	Mellow ML, Dumuid D, Thacker JS, Dorrian J, Smith AE. Building your best day for healthy brain aging-The neuroprotective effects of optimal time use. Maturitas. 2019;125:33-40.

602.	Mellow ML, Dumuid D, Wade AT, Stanford T, Olds TS, Karayanidis F, et al. Twenty-four-hour time-use composition and cognitive function in older adults: Cross-sectional findings of the ACTIVate study. Frontiers in Human Neuroscience. 2022;16((Mellow, Dumuid, Wade, Stanford, Olds, Smith) Alliance for Research in Exercise, Nutrition and Activity, Allied Health and Human Performance, University of South Australia, Adelaide, SA, Australia(Karayanidis, Hunter) Functional Neuroimaging Laboratory, S):1051793.

603.	Mendham AE, Goedecke JH, Kufe NC, Soboyisi M, Smith A, Westgate K, et al. Physical Behaviors and Their Association With Adiposity in Men and Women From a Low-Resourced African Setting. Journal of physical activity & health. 2022;19(8):548-57.

604.	Meneguci J, Galvão LL, Tribess S, Meneguci CAG, Virtuoso Júnior JS. Isotemporal substitution analysis of time between sleep, sedentary behavior, and physical activity on depressive symptoms in older adults: a cross-sectional study. Sao Paulo Medical Journal. 2024;142(4).

605.	Menezes-Júnior LAAD, Barbosa BCR, de Paula W, Ferreira AD, Cardoso CS, de Freitas ED, et al. Isotemporal substitution analysis of time between sedentary behavior, and physical activity on sleep quality in younger adults: a multicenter study. BMC Public Health. 2024;24(1).

606.	Meredith SJ, Shepherd AI, Saynor ZL, Scott A, Gorczynski P, Perissiou M, et al. Device-based 24-hour movement behaviours in adult phase III cardiac rehabilitation service-users during the COVID-19 pandemic: a mixed-methods prospective observational study. Disability and rehabilitation. 2024(9207179, a8i):1-12.

607.	Meredith-Jones K, Galland B, Haszard J, Gray A, Sayers R, Hanna M, et al. Do young children consistently meet 24-h sleep and activity guidelines? A longitudinal analysis using actigraphy. International Journal of Obesity. 2019;43(12):2555-64.

608.	Messaadi N, Bayen S, Beghin L, Lefebvre JM, Colleau S, Deken V, et al. Association between screen time and sleep habits in 11-to-12-year-old French middle school students. Revue d'Epidemiologie et de Sante Publique. 2020;68(3):179-84.

609.	Meyer JD, Ellingson LD, Buman MP, Shook RP, Hand GA, Blair SN. Current and 1-Year Psychological and Physical Effects of Replacing Sedentary Time With Time in Other Behaviors. American Journal of Preventive Medicine. 2020;59(1):12-20.

610.	Miao Y, Lin Q, Jiang Y, Zhao J, Zhang Y, Wang G, et al. Association between 24-hour movements and negative emotions in adolescents in Shanghai. Zhongguo Ertong Baojian Zazhi. 2023;31(5):475-80.

611.	Miatke A, Olds T, Maher C, Fraysse F, Mellow ML, Smith AE, et al. The association between reallocations of time and health using compositional data analysis: a systematic scoping review with an interactive data exploration interface. International Journal of Behavioral Nutrition & Physical Activity. 2023;20(1):1-16.

612.	Micklesfield LK, Westgate K, Smith A, Kufe CN, Mendham AE, Lindsay T, et al. Physical Activity Behaviors of a Middle-Age South African Cohort as Determined by Integrated Hip and Thigh Accelerometry. Medicine and science in sports and exercise. 2022;54(9):1493-505.

613.	Miguel Angel T-S, Pedro Antonio S-M, Javier S-S, Antonio G-H, José Francisco L-G. Is adherence to the 24-Hour Movement Guidelines associated with Mediterranean dietary patterns in adolescents? Appetite. 2022;179:N.PAG-N.PAG.

614.	Migueles JH, Aadland E, Andersen LB, Brønd JC, Chastin SF, Hansen BH, et al. GRANADA consensus on analytical approaches to assess associations with accelerometer-determined physical behaviours (physical activity, sedentary behaviour and sleep) in epidemiological studies. British Journal of Sports Medicine. 2022;56(7):376-84.

615.	Migueles JH, Cadenas-Sanchez C, Esteban-Cornejo I, Torres-Lopez LV, Aadland E, Chastin SF, et al. Associations of objectively-assessed physical activity and sedentary time with hippocampal gray matter volume in children with overweight/obesity. Journal of Clinical Medicine. 2020;9(4):1080.

616.	Migueles JH, Delisle Nyström C, Dumuid D, Leppänen MH, Henriksson P, Löf M. Longitudinal associations of movement behaviours with body composition and physical fitness from 4 to 9 years of age: structural equation and mediation analysis with compositional data. International Journal of Behavioral Nutrition and Physical Activity. 2023;20(1).

617.	Migueles JH, Molina-Garcia P, Torres-Lopez LV, Cadenas-Sanchez C, Rowlands AV, Ebner-Priemer UW, et al. Equivalency of four research-grade movement sensors to assess movement behaviors and its implications for population surveillance. Scientific reports. 2022;12(1):5525.

618.	Mihrshahi S, Baur LA. What exposures in early life are risk factors for childhood obesity? Journal of Paediatrics and Child Health. 2018;54(12):1294-8.

619.	Milanović SM, Križan H, Pavičić L, Morović ML, Baić M. PHYSICAL ACTIVITY RISK BEHAVIOUR IN SCHOOL-AGED CHILDREN IS ASSOCIATED WITH OVERWEIGHT AND MATERNAL CHARACTERISTICS. Kinesiology. 2021;53(2):309-17.

620.	Mitchell JJ, Blodgett JM, Chastin SFM, Jefferis BJ, Wannamethee SG, Hamer M. Exploring the associations of daily movement behaviours and mid-life cognition: A compositional analysis of the 1970 British Cohort Study. Journal of Epidemiology and Community Health. 2023;77(3):189-95.

621.	Mitra R, Moore SA, Gillespie M, Faulkner G, Vanderloo LM, Chulak-Bozzer T, et al. Healthy movement behaviours in children and youth during the COVID-19 pandemic: Exploring the role of the neighbourhood environment. Health & Place. 2020;65:N.PAG-N.PAG.

622.	Mo L, Tong J, Chen X, You Y. The role of education attainment on 24-hour movement behavior in emerging adults: evidence from a population-based study. Frontiers in public health. 2024;12((You, Chen) Division of Sports Science and Physical Education, Tsinghua University, Beijing, China(You, Chen) School of Social Sciences, Tsinghua University, Beijing, China(Mo) School of Law and Humanities, China University of Mining and Technology, Beiji):1197150.

623.	Moitra P, Madan J, Verma P. Independent and combined influences of physical activity, screen time, and sleep quality on adiposity indicators in Indian adolescents. BMC Public Health. 2021;21(1).

624.	Molleri N, Gomes Junior SC, Marano D, Zin A. Survey of the Adequacy of Brazilian Children and Adolescents to the 24-Hour Movement Guidelines before and during the COVID-19 Pandemic. International journal of environmental research and public health. 2023;20(9).

625.	Molleri N, Gomes Junior SC, Marano D, Zin A. Adherence of Brazilian Minors to the 24-Hour Movement Guidelines after In-Person School Return. International Journal of Environmental Research and Public Health. 2024;21(7).

626.	Montoye AHK, Clevenger KA, Boudreaux BD, Schmidt MD. Understanding Physical Behaviors During Periods of Accelerometer Wear and Nonwear in College Students. Journal for the Measurement of Physical Behaviour. 2023;6(4):298-314.

627.	Montoye AHK, Clevenger KA, Boudreaux BD, Schmidt MD. Comparability of 24-hr Activity Cycle Outputs From ActiGraph Counts Generated in ActiLife and RStudio. Journal for the Measurement of Physical Behaviour. 2024;7(1).

628.	Moore SA, Faulkner G, Rhodes RE, Brussoni M, Chulak-Bozzer T, Ferguson LJ, et al. Impact of the COVID-19 virus outbreak on movement and play behaviours of Canadian children and youth: A national survey. International Journal of Behavioral Nutrition and Physical Activity. 2020;17(1).

629.	Moore SA, Faulkner G, Rhodes RE, Vanderloo LM, Ferguson LJ, Guerrero MD, et al. Few Canadian children and youth were meeting the 24-hour movement behaviour guidelines 6-months into the COVID-19 pandemic: Follow-up from a national study. Applied physiology, nutrition, and metabolism = Physiologie appliquee, nutrition et metabolisme. 2021;46(10):1225-40.

630.	Moore SA, Sharma R, Martin Ginis KA, Arbour-Nicitopoulos KP. Adverse effects of the COVID-19 pandemic on movement and play behaviours of children and youth living with disabilities: Findings from the national physical activity measurement (NPAM) study. International Journal of Environmental Research and Public Health. 2021;18(24).

631.	Moore SA, Vanderloo LM, Birkenmd MCS, Rehman LA. Reimagining healthy movement in the era of the COVID-19 pandemic. Health Promotion and Chronic Disease Prevention in Canada. 2022;42(4):125-8.

632.	Morgan TL, Faught E, Ross-White A, Fortier MS, Duggan M, Jain R, et al. Tools to guide clinical discussions on physical activity, sedentary behaviour, and/or sleep for health promotion between primary care providers and adults accessing care: a scoping review. BMC Primary Care. 2023;24(1):1-17.

633.	Morgan TL, Pletch J, Faught E, Fortier MS, Gazendam MK, Howse K, et al. Developing and testing the usability, acceptability, and future implementation of the Whole Day Matters Tool and User Guide for primary care providers using think-aloud, near-live, and interview procedures. BMC medical informatics and decision making. 2023;23(1):57.

634.	Morgan TL, Suart TN, Fortier MS, Tomasone JR. Moving toward co-production: five ways to get a grip on collaborative implementation of Movement Behaviour curricula in undergraduate medical education. Canadian medical education journal. 2022;13(5):87-100.

635.	Morrison S, Haszard JJ, Galland BC, Jackson R, Meredith-Jones KA, Elder DE, et al. Where does the time go when children don't sleep? A randomized crossover study. Obesity. 2023;31(3):625-34.

636.	Morrison SA, Meh K, Sember V, Starc G, Jurak G. The Effect of Pandemic Movement Restriction Policies on Children's Physical Fitness, Activity, Screen Time, and Sleep. Frontiers in Public Health. 2021;9.

637.	Mota JG, Clark CCT, Bezerra TA, Lemos L, Reuter CP, Mota JAPS, et al. Twenty-four-hour movement behaviours and fundamental movement skills in preschool children: A compositional and isotemporal substitution analysis. Journal of Sports Sciences. 2020;38(18):2071-9.

638.	Moura ARLI, do Nascimento Moura M, Cabral PUL, Carvalho FO, de Moraes JFVN. Physical activity, screen time, and sleep duration of adolescents before and during COVID-19. Mundo da Saude. 2023;47(1).

639.	Munambah N, Gretschel P, Muchirahondo F, Chiwaridzo M, Chikwanha T, Kariippanon KE, et al. 24 hour movement behaviours and the health and development of pre-school children from Zimbabwean settings: the SUNRISE pilot study. South African journal of sports medicine. 2021;33(1):v33i1a10864.

640.	Murphy A, Graham K, Olds T, Loughry C, Fraysse F, Dumuid D, et al. Measuring 24-h use of time in people with a diabetes-related foot ulcer: A feasibility study. Journal of foot and ankle research. 2024;17(3):e12045.

641.	Murray RM, Doré I, Sabiston CM, Michael F, O'Loughlin JL. A time compositional analysis of the association between movement behaviors and indicators of mental health in young adults. Scandinavian Journal of Medicine & Science in Sports. 2023;33(12):2598-607.

642.	Na X, Chen Y, Ma X, Wang D, Wang H, Song Y, et al. Relations of lifestyle behavior clusters to dyslipidemia in china: A compositional data analysis. International Journal of Environmental Research and Public Health. 2021;18(15):7763.

643.	Narcisse MR, Long CR, Felix HC, Howie EK, Purvis RS, McElfish PA. Adherence to sleep guidelines reduces risk of overweight/obesity in addition to 8-5-2-1-0 guidelines among a large sample of adolescents in the United States. Sleep Health. 2019;5(5):444-51.

644.	Nascimento-Ferreira MV, Marin KA, Abrao Ferreira RK, Oliveira LF, Bandeira AC, Silva Sousa P, et al. 24 h movement behavior and metabolic syndrome study protocol: A prospective cohort study on lifestyle and risk of developing metabolic syndrome in undergraduate students from low-income regions during a pandemic. Frontiers in epidemiology. 2022;2(9918419158106676):1010832.

645.	Nauha L, Farrahi V, Jurvelin H, Jämsä T, Niemelä M, Ala-Mursula L, et al. Regularity of bedtime, wake-up time, and time in bed in mid-life: associations with cardiometabolic health markers with adjustment for physical activity and sedentary time. Journal of Activity, Sedentary and Sleep Behaviors. 2024;3(1).

646.	Nazarali S, Robinson CH, Khan F, Pocsai T, Desai D, De Souza RJ, et al. Deriving Normative Data on 24-Hour Ambulatory Blood Pressure Monitoring for South Asian Children (ASHA): A Clinical Research Protocol. Canadian Journal of Kidney Health and Disease. 2022;9.

647.	Neville RD, Hopkins WG, McArthur BA, Draper CE, Madigan S. Associations Between Changes in 24-Hour Movement Behaviors in Children and Adolescents During the COVID-19 Pandemic: A Systematic Review and Mediation-Based Meta-Analysis. Journal of physical activity & health. 2024;21(4):323-32.

648.	Ng E, Wake M, Olds T, Lycett K, Edwards B, Ha L, et al. Equivalence Curves for Healthy Lifestyle Choices. Pediatrics. 2021;147(4):1-12.

649.	Ng JYY, He Q, Chong KH, Okely AD, Chan CHS, Ha AS. The impact of covid-19 on preschool-aged children's movement behaviors in Hong Kong: A longitudinal analysis of accelerometer-measured data. International Journal of Environmental Research and Public Health. 2021;18(22):1907.

650.	Nielsen MS, Alsaoodi H, Hjorth MF, Sjodin A. Physical Activity, Sedentary Behavior, and Sleep Before and After Bariatric Surgery and Associations with Weight Loss Outcome. Obesity Surgery. 2021;31(1):250-9.

651.	Niemelä M, Kiviniemi A, Ikäheimo TM, Tulppo M, Korpelainen R, Jämsä T, et al. Compositional association of 24‐h movement behavior with incident major adverse cardiac events and all‐cause mortality. Scandinavian Journal of Medicine & Science in Sports. 2023;33(5):641-50.

652.	Niemelä M, Maijala A, Nauha L, Jämsä T, Korpelainen R, Farrahi V. Associations of Wearable Ring Measured Sleep, Sedentary Time, and Physical Activity With Cardiometabolic Health: A Compositional Data Analysis Approach. Scandinavian Journal of Medicine & Science in Sports. 2024;34(8):1-12.

653.	Nystrom CD, Alexandrou C, Henstrom M, Nilsson E, Okely AD, El Masri SW, et al. International study of movement behaviors in the early years (Sunrise): Results from sunrise sweden's pilot and covid-19 study. International Journal of Environmental Research and Public Health. 2020;17(22):1-12.

654.	O'Neill CD, Vidal-Almela S, Terada T, Way KL, Kamiya K, Sperlich B, et al. Moving Together While Staying Apart: Practical Recommendations for 24-Hour Home-Based Movement Behaviours for Those With Cardiovascular Disease. CJC open. 2021;3(12):1495-504.

655.	Ohta T, Ogawa M, Kikuchi N, Sasai H, Okamoto T. Adherence to 24-h Movement Guidelines and Depressive Status During the Coronavirus Disease Outbreak: A Cross-Sectional Japanese Survey. International Journal of Public Health. 2023;68.

656.	Okely AD, Ghersi D, Hesketh KD, Santos R, Loughran SP, Cliff DP, et al. A collaborative approach to adopting/adapting guidelines - The Australian 24-Hour Movement Guidelines for the early years (Birth to 5 years): an integration of physical activity, sedentary behavior, and sleep. BMC Public Health. 2017;17:167-90.

657.	Okely AD, Ghersi D, Loughran SP, Cliff DP, Shilton T, Jones RA, et al. A collaborative approach to adopting/adapting guidelines. The Australian 24-hour movement guidelines for children (5-12 years) and young people (13-17 years): An integration of physical activity, sedentary behaviour, and sleep. International Journal of Behavioral Nutrition & Physical Activity. 2022;19(1):1-21.

658.	Okely AD, Kariippanon KE, Guan H, Taylor EK, Suesse T, Cross PL, et al. Global effect of COVID-19 pandemic on physical activity, sedentary behaviour and sleep among 3- to 5-year-old children: a longitudinal study of 14 countries. BMC Public Health. 2021;21(1).

659.	Okely T, Reilly JJ, Tremblay MS, Kariippanon KE, Draper CE, El Hamdouchi A, et al. Cross-sectional examination of 24-hour movement behaviours among 3- and 4-year-old children in urban and rural settings in low-income, middle-income and high-income countries: the SUNRISE study protocol. BMJ open. 2021;11(10):e049267.

660.	Okusanya D, Ezeugwa JC, Khan A, Buck B, Jickling GC, Ezeugwu VE. The whole day matters after stroke: Study protocol for a randomized controlled trial investigating the effect of a 'sit less, move more, sleep better' program early after stroke. PLoS ONE. 2023;18(12 December).

661.	Olds T, Burton NW, Sprod J, Maher C, Ferrar K, Brown WJ, et al. One day you’ll wake up and won’t have to go to work: The impact of changes in time use on mental health following retirement. PLoS ONE. 2018;13(6).

662.	Olds T, Ferrar KE, Gomersall SR, Maher C, Walters JL. The Elasticity of Time: Associations Between Physical Activity and Use of Time in Adolescents. Health Education and Behavior. 2012;39(6):732-6.

663.	Olds T, Sanders I, Maher C, Fraysse F, Bell L, Leslie E. Does compliance with healthy lifestyle behaviours cluster within individuals in Australian primary school-aged children? Child: Care, Health and Development. 2018;44(1):117-23.

664.	Olds T, Singh B, Miatke A, Eglitis E, Maher C, Dumuid D. The Association Between Socioeconomic Status and Use of Time in Australian Children and Adolescents. Journal of Adolescent Health. 2023;73(6):1068-76.

665.	Olds TS, Maher CA, Matricciani L. Sleep duration or bedtime? Exploring the relationship between sleep habits and weight status and activity patterns. Sleep: Journal of Sleep and Sleep Disorders Research. 2011;34(10):1299-307.

666.	Onambele-Pearson G, Wullems J, Doody C, Ryan D, Morse C, Degens H. Influence of habitual physical behavior - Sleeping, sedentarism, physical activity - on bone health in community-dwelling older people. Frontiers in Physiology. 2019;10(MAR):408.

667.	Orme MW, Steiner MC, Morgan MD, Kingsnorth AP, Esliger DW, Singh SJ, et al. 24-hour accelerometry in COPD: Exploring physical activity, sedentary behavior, sleep and clinical characteristics. International Journal of COPD. 2019;14((Orme, Steiner, Morgan, Singh) Centre for Exercise and Rehabilitation Science, NIHR Leicester Biomedical Research Centre, Respiratory, Leicester, United Kingdom(Kingsnorth, Esliger, Sherar) School of Sport, Exercise and Health Sciences, Loughborough Unive):419-30.

668.	Ortolá R, García-Esquinas E, Buño-Soto A, Cabanas-Sánchez V, Martínez-Gómez D, Sotos-Prieto M, et al. Associations of device-measured sleep, sedentariness and physical activity with growth differentiation factor 15 in older adults. Journal of Cachexia, Sarcopenia and Muscle. 2022;13(2):1003-12.

669.	Ortolá R, García-Esquinas E, Cabanas-Sánchez V, Migueles JH, Martínez-Gómez D, Rodríguez-Artalejo F. Association of physical activity, sedentary behavior, and sleep with unhealthy aging: Consistent results for device-measured and self-reported behaviors using isotemporal substitution models. Journals of Gerontology - Series A Biological Sciences and Medical Sciences. 2021;76(1):85-94.

670.	Oviedo-Caro MA, Bueno-Antequera J, Munguia-Izquierdo D. Associations of 24-hours activity composition with adiposity and cardiorespiratory fitness: The PregnActive project. Scandinavian journal of medicine & science in sports. 2020;30(2):295-302.

671.	Padmapriya N, Chen B, Goh CMJL, Shek LPC, Chong YS, Tan KH, et al. 24-hour movement behaviour profiles and their transition in children aged 5.5 and 8 years – findings from a prospective cohort study. International Journal of Behavioral Nutrition & Physical Activity. 2021;18(1):1-14.

672.	Padmapriya N, Fogel A, Tan SYX, Goh CMJL, Tan SL, Chia A, et al. The cross-sectional and prospective associations of parental practices and environmental factors with 24-hour movement behaviours among school-aged Asian children. International Journal of Behavioral Nutrition & Physical Activity. 2024;21(1):1-14.

673.	Paiement K, Drapeau V, Gilbert JA, Lemoyne J, Moreau N, Monthuy-Blanc J, et al. Changes in lifestyle habits among adolescent girls after fitspirit participation. International Journal of Environmental Research and Public Health. 2020;17(12):1-14.

674.	Pan N, Lin LZ, Nassis GP, Wang X, Ou XX, Cai L, et al. Adherence to 24-hour movement guidelines in children with mental, behavioral, and developmental disorders: Data from the 2016-2020 National Survey of Children's Health. Journal of Sport and Health Science. 2023;12(3):304-11.

675.	Park C, Larsen B, Kwon S, Xia Y, Dickson VV, Kim SS, et al. Acculturation, Discrimination and 24-h Activity in Asian American Immigrant Women. Journal of Immigrant and Minority Health. 2022;24(4):1005-12.

676.	Park C, Larsen B, Kwon SC, Xia Y, LaNoue M, Dickson VV, et al. Reallocating time between device-measured 24-hour activities and cardiovascular risk in Asian American immigrant women: An isotemporal substitution model. PLoS ONE. 2024;19(1 January):e0297042.

677.	Parker H, Burkart S, Reesor-Oyer L, von Klinggraeff L, Pfledderer CD, Adams E, et al. The Day-Level Association Between Child Care Attendance and 24-Hour Movement Behaviors in Preschool-Aged Children. Journal of physical activity & health. 2024;21(6):616-23.

678.	Pasanen J, Leskinen T, Suorsa K, Pulakka A, Virta J, Auranen K, et al. Effects of physical activity intervention on 24-h movement behaviors: a compositional data analysis. Scientific reports. 2022;12(1):8712.

679.	Patel P, Li X, Keown-Stoneman CDG, Vanderloo LM, Kinlin LM, Maguire JL, et al. Changes in Pediatric Movement Behaviors During the COVID-19 Pandemic by Stages of Lockdown in Ontario, Canada: A Longitudinal Cohort Study. Journal of physical activity & health. 2023;20(4):292-302.

680.	Paterson DC, Ramage K, Moore SA, Riazi N, Tremblay MS, Faulkner G. Exploring the impact of COVID-19 on the movement behaviors of children and youth: A scoping review of evidence after the first year. Journal of sport and health science. 2021;10(6):675-89.

681.	Patience M, Janssen X, Kirk A, McCrory S, Russell E, Hodgson W, et al. 24-Hour Movement Behaviours (Physical Activity, Sedentary Behaviour and Sleep) Association with Glycaemic Control and Psychosocial Outcomes in Adolescents with Type 1 Diabetes: A Systematic Review of Quantitative and Qualitative Studies. International journal of environmental research and public health. 2023;20(5).

682.	Patte KA, Faulkner G, Qian W, Duncan M, Leatherdale ST. Are one-year changes in adherence to the 24-hour movement guidelines associated with depressive symptoms among youth? BMC public health. 2020;20(1):793.

683.	Patte KA, Qian W, Cole AG, Faulkner G, Chaput JP, Carson V, et al. School start time changes in the COMPASS study: associations with youth sleep duration, physical activity, and screen time. Sleep Medicine. 2019;56:16-22.

684.	Peddie MC, Scott T, Haszard JJ. Using a 24 h Activity Recall (STAR-24) to Describe Activity in Adolescent Boys in New Zealand: Comparisons between a Sample Collected before, and a Sample Collected during the COVID-19 Lockdown. International journal of environmental research and public health. 2021;18(15).

685.	Pedišić Z. Measurement issues and poor adjustments for physical activity and sleep undermine sedentary behaviour research - The focus should shift to the balance between sleep, sedentary behaviour, standing and activity. Kinesiology. 2014;46(1):135-46.

686.	Pedišić Ž, Dumuid D, Olds TS. Integrating sleep, sedentary behaviour, and physical activity research in the emerging field of time-use epidemiology: Definitions, concepts, statistical methods, theoretical framework, and future directions. Kinesiology. 2017;49(2):252-69.

687.	Pellerine LP, Bray NW, Fowles JR, Furlano JA, Morava A, Nagpal TS, et al. Increased recreational screen time and time to fall asleep are associated with worse academic performance in Canadian undergraduates. International Journal of Health Promotion and Education. 2023.

688.	Peng B, Reeves KKL, Lee SWY, Chung THY, Hui HWL, Leung AHL, et al. Physical, psychological, and behavioral problems among children and adolescents in countries with different economic statuses during the COVID-19 pandemic: a systematic review and meta-analysis. Frontiers in Pediatrics. 2023;11.

689.	Peralta GP, Camerini AL, Haile SR, Kahlert CR, Lorthe E, Marciano L, et al. Lifestyle Behaviours of Children and Adolescents During the First Two Waves of the COVID-19 Pandemic in Switzerland and Their Relation to Well-Being: An Observational Study. International Journal of Public Health. 2022;67.

690.	Perez LG, Dong L, Beckman R, Meadows SO. Movement behaviors associated with mental health among US military service members. Military Psychology. 2022;34(2):211-23.

691.	Pessoa ML, Clark C, Souza A, Nakamura F, Brasileiro-Santos MDS, Ribeiro A, et al. The 24-h movement behaviours composition is associated with parasympathetic modulation in preschoolers. Child: Care, Health and Development. 2024;50(3).

692.	Pettee Gabriel K, Sternfeld B, Shiroma EJ, Pérez A, Cheung J, Lee IM. Bidirectional associations of accelerometer-determined sedentary behavior and physical activity with reported time in bed: Women's Health Study. Sleep Health. 2017;3(1):49-55.

693.	Pfefferbaum B, Van Horn RL. Physical Activity and Sedentary Behavior in Children During the COVID-19 Pandemic: Implications for Mental Health. Current Psychiatry Reports. 2022;24(10):493-501.

694.	Pfledderer CD, Beets MW, Burkart S, Adams EL, Weaver RG, Zhu X, et al. Impact of Virtual vs. In-Person School on Children Meeting the 24-h Movement Guidelines during the COVID-19 Pandemic. International Journal of Environmental Research and Public Health. 2022;19(18).

695.	Pfledderer CD, Burkart S, Dugger R, Parker H, von Klinggraeff L, Okely AD, et al. The impact of different data handling strategies on the proportion of children classified as meeting 24-h movement guidelines and associations with overweight and obesity. Journal of Activity, Sedentary and Sleep Behaviors. 2024;3(1).

696.	Phillips SM, Summerbell C, Hesketh KR, Saxena S, Hillier-Brown FC. Parental Views on the Acceptability and Feasibility of Measurement Tools Used to Assess Movement Behaviour of Pre-School Children: A Qualitative Study. International Journal of Environmental Research and Public Health. 2022;19(6).

697.	Phillips SM, Summerbell C, Hesketh KR, Saxena S, Hillier-Brown FC. Co-design and content validity of the movement measurement in the early years (MoveMEY) tool for assessing movement behaviour of pre-school aged children. International Journal of Behavioral Nutrition and Physical Activity. 2023;20(1).

698.	Pina I, Hodgson P, Anderson K, Oliver EJ. Speak for yourself: usability and acceptability of audio diaries to explore physical activity, sedentary and sleep behaviours of those living with severe mental illness. Journal of Activity, Sedentary and Sleep Behaviors. 2024;3(1):21-.

699.	Pina I, Mendham AE, Tomaz SA, Goedecke JH, Micklesfield LK, Brooks NE, et al. Intensity matters for musculoskeletal health: A cross-sectional study on movement behaviors of older adults from high-income scottish and low-income South African communities. International Journal of Environmental Research and Public Health. 2021;18(8):4310.

700.	Plekhanova T, Crawley E, Davies MJ, Gorely T, Harrington DM, Ioannidou E, et al. Association between Chronotype and Physical Behaviours in Adolescent Girls. Children. 2023;10(5):819.

701.	Plekhanova T, Rowlands AV, Evans RA, Edwardson CL, Bishop NC, Bolton CE, et al. Device-assessed sleep and physical activity in individuals recovering from a hospital admission for COVID-19: a multicentre study. International Journal of Behavioral Nutrition and Physical Activity. 2022;19(1).

702.	Porter CD, McPhee PG, Kwan MY, Timmons BW, Brown DMY. 24-hour movement guideline adherence and mental health: A cross-sectional study of emerging adults with chronic health conditions and disabilities. Disability and Health Journal. 2023;16(3):101476.

703.	Powell C, Browne LD, Carson BP, Dowd KP, Perry IJ, Kearney PM, et al. Use of Compositional Data Analysis to Show Estimated Changes in Cardiometabolic Health by Reallocating Time to Light-Intensity Physical Activity in Older Adults. Sports Medicine. 2020;50(1):205-17.

704.	Powers B, Patterson F, Palmiere K, Healy S. "I sit all of the time": Health-related time-use among adults with intellectual disabilities. Research in Developmental Disabilities. 2021;108:N.PAG-N.PAG.

705.	Prince SA, Biswas A, Betancourt MT, Toigo S, Roberts KC, Colley RC, et al. Telework and 24-hour movement behaviours among adults living in Canada during the COVID-19 pandemic. Preventive medicine. 2024;185(pm4, 0322116):108053.

706.	Prince SA, Lang JJ, Betancourt M, Toigo S, Roberts KC. Sedentary time at school and work in Canada. Canadian Journal of Public Health. 2024;115(2):343-55.

707.	Quah PL, Loo BKG, Chia MYH, Chua TBK, Tan TSZ, Chan PC, et al. Adherence to 24-hour integrated activity guidelines among infants, toddlers and preschool children in Singapore. PLoS ONE. 2024;19(2 February).

708.	Quah PL, Loo BKG, Mettananda S, Dassanayake S, Chia MYH, Chua TBK, et al. 24 h Activity Guidelines in Children and Adolescents: A Prevalence Survey in Asia-Pacific Cities. International Journal of Environmental Research and Public Health. 2023;20(14).

709.	Quante M, Kaplan ER, Rueschman M, Cailler M, Buxton OM, Redline S. Practical considerations in using accelerometers to assess physical activity, sedentary behavior, and sleep. Sleep Health. 2015;1(4):275-84.

710.	Rahayu NI, Suherman A, Sultoni K, Muktiarni M, Maryanti R. TECHNOLOGY'S INFLUENCE ON PATTERN OF PHYSICAL ACTIVITY AND SEDENTARY BEHAVIORS AMONG EARLY CHILDHOOD. Journal of Engineering Science and Technology. 2023;18(1):453-62.

711.	Ramer JD, Santiago-Rodriguez ME, Vukits AJ, Bustamante EE. The convergent effects of primary school physical activity, sleep, and recreational screen time on cognition and academic performance in grade 9. Frontiers in Human Neuroscience. 2022;16((Ramer, Vukits, Bustamante) Healthy Kids Lab, Department of Kinesiology and Nutrition, University of Illinois Chicago, Chicago, IL, United States(Santiago-Rodriguez) Child Movement, Activity, and Developmental Health Laboratory, School of Kinesiology, Uni):1017598.

712.	Ramírez‐Vélez R, Izquierdo M, López‐Gil JF, Rincón‐Pabón D, Martínez‐Jamioy EN, Rivera‐Ruíz R, et al. Prevalence of meeting all three 24‐h movement guidelines and its correlates among preschool‐aged children. Scandinavian Journal of Medicine & Science in Sports. 2023;33(6):979-88.

713.	Rao DP, Orpana H, Krewski D. Physical activity and non-movement behaviours: Their independent and combined associations with metabolic syndrome. International Journal of Behavioral Nutrition and Physical Activity. 2016;13(1):26.

714.	Rees-Punia E, Guinter MA, Gapstur SM, Wang Y, Patel AV. Composition of time in movement behaviors and weight change in latinx, black and white participants. PLoS ONE. 2021;16(1 January):e0244566.

715.	Rees-Punia E, Newton CC, Rittase MH, Hodge RA, Nielsen J, Cunningham S, et al. Prospective changes in physical activity, sedentary time and sleep during the COVID-19 pandemic in a US-based cohort study. BMJ Open. 2021;11(12):e053817.

716.	Reilly JJ, Hughes AR, Janssen X, Hesketh KR, Livingstone S, Hill C, et al. GRADE-ADOLOPMENT Process to Develop 24-Hour Movement Behavior Recommendations and Physical Activity Guidelines for the Under 5s in the United Kingdom, 2019. Journal of physical activity & health. 2020;17(1):101-8.

717.	Reis LN, Reuter CP, Okely A, Brand C, Fochesatto CF, Martins CML, et al. Replacing screen time, with physical activity and sleep time: influence on cardiovascular indicators and inflammatory markers in Brazilian children. Jornal de Pediatria. 2024;100(2):149-55.

718.	Ren Z, Tan J, Huang B, Cheng J, Huang Y, Xu P, et al. Association between 24-Hour Movement Behaviors and Smartphone Addiction among Adolescents in Foshan City, Southern China: Compositional Data Analysis. International Journal of Environmental Research and Public Health. 2022;19(16):9942.

719.	Rhodes RE, Guerrero MD, Vanderloo LM, Barbeau K, Birken CS, Chaput J-P, et al. Development of a consensus statement on the role of the family in the physical activity, sedentary, and sleep behaviours of children and youth. International Journal of Behavioral Nutrition & Physical Activity. 2020;17(1):1-31.

720.	Rhodes RE, Spence JC, Berry T, Faulkner G, Latimer-Cheung AE, O'Reilly N, et al. Parental support of the Canadian 24-hour movement guidelines for children and youth: Prevalence and correlates. BMC Public Health. 2019;19(1).

721.	Rhodes RE, Stearns J, Berry T, Faulkner G, Latimer-Cheung AE, O'Reilly N, et al. Predicting parental support and parental perceptions of child and youth movement behaviors. Psychology of Sport & Exercise. 2019;41:80-90.

722.	Riad RR, Aly MG, Mohamed NE. Movement and Play Behaviors of Egyptian Children After Covid-19 Pandemic Restrictions: Cross-Sectional Study. Egyptian Journal of Hospital Medicine. 2022;88(1):3892-9.

723.	Riazi N, Ramanathan S, O'Neill M, Tremblay MS, Faulkner G. Canadian 24-hour movement guidelines for the early years (0-4 years): exploring the perceptions of stakeholders and end users regarding their acceptability, barriers to uptake, and dissemination. BMC public health. 2017;17(Supplement 5):841.

724.	Riquelme R, Rezende LFM, Marques A, Drenowatz C, Ferrari G. Association between 24-h movement guidelines and cardiometabolic health in Chilean adults. Scientific reports. 2022;12(1):5805.

725.	Rivera E, Hesketh KD, Orellana L, Taylor R, Carson V, Nicholson JM, et al. Prevalence of toddlers meeting 24-hour movement guidelines and associations with parental perceptions and practices. Journal of science and medicine in sport. 2024;27(4):250-6.

726.	Roberts KC, Yao X, Carson V, Chaput JP, Janssen I, Tremblay MS. Meeting the Canadian 24-Hour Movement Guidelines for Children and Youth. Health reports. 2017;28(10):3-7.

727.	Rodrigo-Sanjoaquín J, Bois JE, Aibar Solana A, Lhuisset L, Corral-Abós A, Zaragoza Casterad J. Are school-based interventions promoting 24-hour movement guidelines among children? A scoping review. Health Education Journal. 2023;82(4):444-60.

728.	Rodrigo-Sanjoaquín J, Bois JE, Solana AA, Lhuisset L, Casterad JZ. Identifying promising school-based intervention programs to promote 24-hour movement guidelines among children: Protocol for a systematic review. Sustainability (Switzerland). 2020;12(22):1-11.

729.	Rodrigues B, Encantado J, Carraca E, Sousa-Sa E, Lopes L, Cliff D, et al. Questionnaires measuring movement behaviours in adults and older adults: Content description and measurement properties. A systematic review. PloS one. 2022;17(3):e0265100.

730.	Rollo S, Antsygina O, Tremblay MS. The whole day matters: Understanding 24-hour movement guideline adherence and relationships with health indicators across the lifespan. Journal of sport and health science. 2020;9(6):493-510.

731.	Rollo S, Lang JJ, Roberts KC, Bang F, Carson V, Chaput JP, et al. Health associations with meeting the Canadian 24-hour movement guidelines for adults: Results from the Canadian Health Measures Survey. Health reports. 2022;33(1):16-26.

732.	Rollo S, Roberts KC, Bang F, Carson V, Chaput JP, Colley RC, et al. Sociodemographic Factors Associated With Meeting the Canadian 24-Hour Movement Guidelines Among Adults: Findings From the Canadian Health Measures Survey. Journal of physical activity & health. 2022;19(3):194-202.

733.	Rollo S, Sckrapnick A, Campbell JE, Moore SA, Faulkner G, Tremblay MS. Prevalence and correlates of meeting the Canadian 24-hour movement guidelines among a sample of Canadian parents during the COVID-19 pandemic. Journal of Activity, Sedentary and Sleep Behaviors. 2023;2(1).

734.	Roman-Vinas B, Chaput J-P, Katzmarzyk PT, Fogelholm M, Lambert EV, Maher C, et al. Proportion of children meeting recommendations for 24-hour movement guidelines and associations with adiposity in a 12-country study. The international journal of behavioral nutrition and physical activity. 2016;13(1):123.

735.	Ronen GM, Janssen I. Patterns of daily activity among young people with epilepsy. Developmental Medicine & Child Neurology. 2019;61(12):1386-91.

736.	Rorem D, Ezeugwu VE, Joly VJ, Rasmussen C, Carson V, Tamana SK, et al. Finding the balance: The influence of movement behaviours on childhood behaviour problems. Mental Health and Physical Activity. 2024;26.

737.	Rosenberg DE, Wu Y, Idu A, Greenwood-Hickman MA, McCurry SM, LaCroix AZ, et al. Historic Cognitive Function Trajectories as Predictors of Sedentary Behavior and Physical Activity in Older Adults. Journals of Gerontology - Series A Biological Sciences and Medical Sciences. 2024;79(7).

738.	Rosenberger ME, Buman MP, Haskell WL, McConnell MV, Carstensen LL. Twenty-four Hours of Sleep, Sedentary Behavior, and Physical Activity with Nine Wearable Devices. Medicine and Science in Sports and Exercise. 2016;48(3):457-65.

739.	Rosenberger ME, Fulton JE, Buman MP, Troiano RP, Grandner MA, Buchner DM, et al. The 24-Hour Activity Cycle: A New Paradigm for Physical Activity. Medicine and science in sports and exercise. 2019;51(3):454-64.

740.	Ross R, Chaput J-P, Giangregorio LM, Janssen I, Saunders TJ, Kho ME, et al. Canadian 24-Hour Movement Guidelines for Adults aged 18–64 years and Adults aged 65 years or older: an integration of physical activity, sedentary behaviour, and sleep. Applied Physiology, Nutrition & Metabolism. 2020;45:S57-S102.

741.	Ross R, Tremblay M. Introduction to the Canadian 24-Hour Movement Guidelines for Adults aged 18–64 years and Adults aged 65 years or older: an integration of physical activity, sedentary behaviour, and sleep. Applied Physiology, Nutrition & Metabolism. 2020;45:v-xi.

742.	Ross SM, Haegele JA, Abrahamson K, Schram BM, Healy S. US adults with visual impairments meeting 24-h movement guidelines: Updated national prevalence estimates. Disability and health journal. 2022;15(3):101320.

743.	Ross SM, Haegele JA, Schram BM, Healy S. 24-Hour Movement Behaviors Among US Adults With Functional Disabilities. Journal of physical activity & health. 2022;19(5):392-7.

744.	Rowlands AV. Physical Activity, Inactivity and Health During Youth-2016. Pediatric exercise science. 2017;29(1):26-30.

745.	Rowlands AV. Moving Forward With Accelerometer-Assessed Physical Activity: Two Strategies to Ensure Meaningful, Interpretable, and Comparable Measures. Pediatric Exercise Science. 2018;30(4):450-6.

746.	Rubín L, Gába A, Dygrýn J, Jakubec L, Materová E, Vencálek O. Prevalence and correlates of adherence to the combined movement guidelines among Czech children and adolescents. BMC Public Health. 2020;20(1):1-11.

747.	Runacres A, MacKintosh KA, Chastin S, McNarry MA. The associations of physical activity, sedentary time, and sleep with VO ̇2max in trained and untrained children and adolescents: A novel five-part compositional analysis. PLoS ONE. 2023;18(3 March).

748.	Ryan DJ, Wullems JA, Stebbings GK, Morse CI, Stewart CE, Onambele-Pearson GL. The difference in sleep, sedentary behaviour, and physical activity between older adults with 'healthy' and 'unhealthy' cardiometabolic profiles: a cross-sectional compositional data analysis approach. European review of aging and physical activity : official journal of the European Group for Research into Elderly and Physical Activity. 2019;16(101284836):25.

749.	Ryan ST, Okely AD, Chong KH, Stanley RM, Randle M, Waqa G, et al. Proportion and Correlates of Children in the US-Affiliated Pacific Region Meeting Sleep, Screen Time, and Physical Activity Guidelines. Journal of physical activity & health. 2024;21(6):567-77.

750.	Salas-González MD, Bermejo LM, González-Rodríguez LG, Lozano-Estevan MDC, Ortega RM, López-Sobaler AM. Adherence to 24-h movement guidelines in Spanish schoolchildren and its association with insulin resistance: a cross-sectional study. Frontiers in Public Health. 2023;11.

751.	Sampasa-Kanyinga H, Chaput J-P, Goldfield GS, Janssen I, Wang J, Hamilton HA, et al. The Canadian 24-hour movement guidelines and psychological distress among adolescents. The Canadian Journal of Psychiatry / La Revue canadienne de psychiatrie. 2021;66(7):624-33.

752.	Sampasa-Kanyinga H, Chaput JP, Goldfield GS, Janssen I, Wang J, Hamilton HA, et al. 24-hour movement guidelines and suicidality among adolescents. Journal of Affective Disorders. 2020;274((Sampasa-Kanyinga, Chaput, Goldfield, Wang, Colman) School of Epidemiology and Public Health, University of Ottawa, Ottawa, Ontario, Canada(Chaput, Goldfield) Healthy Active Living and Obesity Research Group, Children's Hospital of Eastern Ontario Researc):372-80.

753.	Sampasa-Kanyinga H, Colman I, Dumuid D, Janssen I, Goldfield GS, Wang JL, et al. Longitudinal association between movement behaviours and depressive symptoms among adolescents using compositional data analysis. PLoS ONE. 2021;16(9 September).

754.	Sampasa-Kanyinga H, Colman I, Goldfield GS, Janssen I, Wang J, Hamilton HA, et al. Associations between the Canadian 24 h movement guidelines and different types of bullying involvement among adolescents. Child Abuse and Neglect. 2020;108.

755.	Sampasa-Kanyinga H, Colman I, Goldfield GS, Janssen I, Wang J, Hamilton HA, et al. 24-h movement guidelines and substance use among adolescents: A school-based cross-sectional study. International Journal of Environmental Research and Public Health. 2021;18(6).

756.	Sampasa-Kanyinga H, Colman I, Goldfield GS, Janssen I, Wang J, Podinic I, et al. Combinations of physical activity, sedentary time, and sleep duration and their associations with depressive symptoms and other mental health problems in children and adolescents: a systematic review. International Journal of Behavioral Nutrition & Physical Activity. 2020;17(1):1-16.

757.	Sampasa-Kanyinga H, Colman I, Goldfield GS, Janssen I, Wang J, Tremblay MS, et al. 24-Hour Movement Behaviors and Internalizing and Externalizing Behaviors Among Youth. Journal of Adolescent Health. 2021;68(5):969-77.

758.	Sampasa-Kanyinga H, Hamilton HA, Chaput J-P. Movement behaviours, breakfast consumption, and fruit and vegetable intake among adolescents. Journal of Activity, Sedentary and Sleep Behaviors. 2022;1(1).

759.	Sampasa-Kanyinga H, Lien A, Hamilton HA, Chaput J-P. The Canadian 24-hour movement guidelines and self-rated physical and mental health among adolescents. Canadian journal of public health = Revue canadienne de sante publique. 2022;113(2):312-21.

760.	Sampasa-Kanyinga H, Lien A, Hamilton HA, Chaput JP. Canadian 24-h Movement Guidelines, Life Stress, and Self-Esteem Among Adolescents. Frontiers in Public Health. 2022;10.

761.	Sampasa-Kanyinga H, Standage M, Tremblay MS, Katzmarzyk PT, Hu G, Kuriyan R, et al. Associations between meeting combinations of 24-h movement guidelines and health-related quality of life in children from 12 countries. Public Health (Elsevier). 2017;153:16-24.

762.	Sandborg J, Migueles JH, Soderstrom E, Blomberg M, Henriksson P, Lof M. Physical Activity, Body Composition, and Cardiometabolic Health during Pregnancy: A Compositional Data Approach. Medicine and science in sports and exercise. 2022;54(12):2054-63.

763.	Santos R, Zhiguang Z, Pereira JR, Sousa-Sá E, Cliff DP, Okely AD, et al. Compliance with the Australian 24-hour movement guidelines for the early years: associations with weight status. BMC Public Health. 2017;17:199-205.

764.	Saunders TJ, Gray CE, Poitras VJ, Chaput JP, Janssen I, Katzmarzyk PT, et al. Combinations of physical activity, sedentary behaviour and sleep: Relationships with health indicators in school-aged children and youth. Applied Physiology, Nutrition and Metabolism. 2016;41(6):S283-S93.

765.	Schaan CW, Schaan BD, Cureau FV. Prevalence and correlates of meeting physical activity, screen time, and sleep duration guidelines among Brazilian adolescents: findings from ERICA. Applied Physiology, Nutrition and Metabolism. 2022;47(6):690-8.

766.	Scully M, Gascoyne C, Wakefield M, Morley B. Prevalence and trends in Australian adolescents' adherence to 24-hour movement guidelines: findings from a repeated national cross-sectional survey. BMC Public Health. 2022;22(1):1-9.

767.	Segura-Jiménez V, Pedišić Ž, Gába A, Dumuid D, Olds T, Štefelová N, et al. Longitudinal reallocations of time between 24-h movement behaviours and their associations with inflammation in children and adolescents: the UP&DOWN study. International Journal of Behavioral Nutrition & Physical Activity. 2023;20(1):1-12.

768.	Sehn AP, Brand C, Silveira JFDC, Marshall R, Renner JDP, Reuter CP. Integrated role of lifestyle habits in cardiometabolic risk factors according to sex in adolescents. Annals of Human Biology. 2022;49(1):18-26.

769.	Sehn AP, Brand C, Tornquist L, Tornquist D, Silveira JFDC, Gaya AR, et al. Sleep duration and screen time in children and adolescents: Simultaneous moderation role in the relationship between waist circumference and cardiometabolic risk according to physical activity. European Journal of Sport Science. 2024;24(2):239-48.

770.	Sejín JRR. Physical activity, sedentary behaviors and sleep. Development, evolution and current context of the 24-hour body movement cycle paradigm and its relationship with the compositional analysis method: documentary review. VIREF-REVISTA DE EDUCACION FISICA. 2022;11(2):40-54.

771.	Sevil J, García-González L, Abós Á, Generelo E, Aibar A. Can High Schools Be an Effective Setting to Promote Healthy Lifestyles? Effects of a Multiple Behavior Change Intervention in Adolescents. Journal of Adolescent Health. 2019;64(4):478-86.

772.	Sevil-Serrano J, Aibar-Solana A, Abos A, Julian JA, Garcia-Gonzalez L. Healthy or unhealthy? The cocktail of health-related behavior profiles in spanish adolescents. International Journal of Environmental Research and Public Health. 2019;16(17):3151.

773.	Shi G, Liang C, Zang W, Bao R, Yan J, Zhou L, et al. 24-hour movement behaviours and self-rated health in Chinese adolescents: a questionnaire-based survey in Eastern China. PeerJ. 2023;11(101603425):e16174.

774.	Shi H, Hu FB, Huang T, Schernhammer ES, Willett WC, Sun Q, et al. Sedentary Behaviors, Light-Intensity Physical Activity, and Healthy Aging. JAMA Network Open. 2024;7(6):e2416300-e.

775.	Shi X, Liu R. Impact of 24 h movement behaviors on fundamental motor skills in preschool children. Chinese Journal of School Health. 2024;45(6):868-72.

776.	Shi Y, Cheng G, Jiang N, Li C, Min X, Yan Y. Association of outdoor activity time and screen time with sleep for 36 to 54 months children in Kaifu District, Changsha: A birth cohort study. Journal of Central South University (Medical Sciences). 2022;47(10):1408-17.

777.	Shi Y, Huang WY, Sit CHP, Wong SHS. Compliance With 24-Hour Movement Guidelines in Hong Kong Adolescents: Associations With Weight Status. Journal of physical activity & health. 2020;17(3):287-92.

778.	Shin SW, Choi Y, Kang YH, Kim J. Associations of meeting 24-h movement guidelines and metabolic syndrome in Korean adults during the COVID-19 pandemic. Public Health. 2024;227:187-93.

779.	Shirazipour CH, Raines C, Diniz MA, Salvy SJ, Haile RW, Freedland SJ, et al. The 24-Hour Movement Paradigm: An integrated approach to the measurement and promotion of daily activity in cancer clinical trials. Contemporary Clinical Trials Communications. 2023;32((Shirazipour, Raines, Diniz, Salvy, Haile, Freedland, Asher, Gresham) Cedars-Sinai Medical Center, Los Angeles, CA, United States(Shirazipour) University of California Los Angeles, Los Angeles, CA, United States(Salvy) University of Southern California, L):101081.

780.	Sigmundova D, Dygryn J, Vorlicek M, Banatova K, Voracova J, Sigmund E. FAMIly Physical Activity, Sedentary behaviour and Sleep (FAMIPASS) study: protocol for a cross-sectional study. BMJ Open. 2023;13(8):e073244.

781.	Silva DAS, Duncan MJ, Kuzik N, Tremblay MS. Associations between anxiety disorders and depression symptoms are related to 24-hour movement behaviors among Brazilian adolescents. Journal of Affective Disorders. 2023;339:280-92.

782.	Silva DAS, Duncan MJ, Kuzik N, Tremblay MS. Do movement behaviours influence the association between early menarche and depression symptoms among Brazilian adolescents? Applied Physiology, Nutrition and Metabolism. 2024;49(9):1137-62.

783.	Silva DAS, Duncan MJ, Kuzik N, Tremblay MS. Race/Ethnicity Inequities in the Association Between Movement Behaviors and Suicidal Thoughts/Ideation Among Adolescents. Journal of Pediatric Psychology. 2024;49(3):166-74.

784.	Singh TD, Sharma HB. How Much Time to Spend in Physical Activity, Sleep and be Sedentary in 24 h to Achieve Good Health? Sleep and Vigilance. 2022;6(2):371-6.

785.	Sit CHP. Promoting 24‐hour movement guideline adherence in ambulatory children with cerebral palsy. Developmental Medicine & Child Neurology. 2023;65(3):305-6.

786.	Skinner AM, Barker AR, Moore SA, Soininen S, Haapala EA, Vaisto J, et al. Cross-sectional and longitudinal associations between the 24-hour movement behaviours, including muscle and bone strengthening activity, with bone and lean mass from childhood to adolescence. BMC public health. 2024;24(1):227.

787.	Smit DJM, Zwinkels M, Takken T, Hulst RY, de Groot JF, Lankhorst K, et al. Sleep quantity and its relation with physical activity in children with cerebral palsy; insights using actigraphy. Journal of Paediatrics and Child Health. 2020;56(10):1618-22.

788.	Smith AE, Dumuid D, Goldsworthy MR, Graetz L, Hodyl N, Thornton NLR, et al. Daily activities are associated with non-invasive measures of neuroplasticity in older adults. Clinical Neurophysiology. 2021;132(4):984-92.

789.	Smith AE, Wade AT, Olds T, Dumuid D, Breakspear MJ, Laver K, et al. Characterising activity and diet compositions for dementia prevention: Protocol for the ACTIVate prospective longitudinal cohort study. BMJ Open. 2022;12(1).

790.	Smith E, Fazeli F, Wilkinson K, Clark CCT. Physical behaviors and fundamental movement skills in British and Iranian children: An isotemporal substitution analysis. Scandinavian Journal of Medicine & Science in Sports. 2021;31(2):398-404.

791.	Soares AHG, Wendt A, Crochemore-Silva I, Martins C, Barbosa AO, de Barros MVG, et al. Prevalence and Sociodemographic Correlates of Meeting the 24-Hour Movement Guidelines Among Low-Income Brazilian Older Adults With Chronic Diseases. Journal of Aging & Physical Activity. 2023;31(5):756-64.

792.	Soltero EG, Navabi N, Vander Wyst KB, Hernandez E, Castro FG, Ayers SL, et al. Examining 24-Hour Activity and Sleep Behaviors and Related Determinants in Latino Adolescents and Young Adults With Obesity. Health Education & Behavior. 2022;49(2):291-303.

793.	Song H, Lau PWC, Wang J, Liu Y, Song Y, Shi L. 24-H movement behaviors and physical fitness in preschoolers: A compositional and isotemporal reallocation analysis. Journal of Exercise Science & Fitness. 2024;22(3):187-93.

794.	Song J, Dunlop DD, Semanik PA, Chang AH, Lee YC, Gilbert AL, et al. Reallocating time spent in sleep, sedentary behavior and physical activity and its association with pain: a pilot sleep study from the Osteoarthritis Initiative. OSTEOARTHRITIS AND CARTILAGE. 2018;26(12):1595-603.

795.	Song Y, Jeon JY. A 24-Hour Movement Behavior for Young Children in South Korea. Exercise Science. 2023;32(2):175-84.

796.	Song Y, Tan S, Qi Y, Feng Z, Zhang G, Cao L. The isochronous substitution effect of 24-hour activity behavior on physical health of college students. Chinese Journal of School Health. 2023;44(9):1382-6.

797.	Song Y, Yoon YJ, Lee HJ, Kim YS, Spence JC, Jeon JY. Development of a 24-Hour Movement Behavior Questionnaire for Youth: Process and Reliability Testing. Journal of Nutrition Education & Behavior. 2021;53(12):1081-9.

798.	Souza AR, Bandeira PFR, da Silva MAC, da Cunha GL, Pereira DF, Martins C. Twenty-Four-Hour Movement Behaviors, Fitness, and Adiposity in Preschoolers: A Network Analysis. OBESITIES. 2023;3(1):36-45.

799.	Spence JC, Kim Y-B, Lamboglia CG, Lindeman C, Mangan AJ, McCurdy AP, et al. Potential Impact of Autonomous Vehicles on Movement Behavior: A Scoping Review. American Journal of Preventive Medicine. 2020;58(6):e191-e9.

800.	Sreelakshmi TP, Sreenivasan P, Krishnan A. Physical Activity, Sedentary Behavior, Sleep and Screen Time of Healthy Under-Fives Attending Selected Immunization Clinics and Anganwadis of South Kerala, India. Indian Pediatrics. 2024;61(3):225-9.

801.	St Laurent CW, Rasmussen CL, Holmes JF, Cremone-Caira A, Kurdziel LBF, Desrochers PC, et al. Associations of activity, sedentary, and sleep behaviors with cognitive and social-emotional health in early childhood. Journal of activity, sedentary and sleep behaviors. 2023;2(1):7.

802.	St. Laurent CW, Andre C, Holmes JF, Fields ND, Spencer RMC. Temporal relationships between device-derived sedentary behavior, physical activity, and sleep in early childhood. Sleep. 2022;45(4):zsac008.

803.	St. Laurent CW, Burkart S, Rodheim K, Marcotte R, Spencer RMC. Cross-sectional associations of 24-hour sedentary time, physical activity, and sleep duration compositions with sleep quality and habits in preschoolers. International Journal of Environmental Research and Public Health. 2020;17(19):1-13.

804.	St. Laurent CW, Holmes JF, Spencer RMC. Temporal Associations between Actigraphy-Measured Daytime Movement Behaviors and Nap Sleep in Early Childhood. International Journal of Environmental Research and Public Health. 2022;19(22):15308.

805.	Stamatakis E, Koster A, Hamer M, Rangul V, Lee IM, Bauman AE, et al. Emerging collaborative research platforms for the next generation of physical activity, sleep and exercise medicine guidelines: The Prospective Physical Activity, Sitting, and Sleep consortium (ProPASS). British Journal of Sports Medicine. 2020;54(8):435-7.

806.	Stamatakis E, Rogers K, Ding D, Berrigan D, Chau J, Hamer M, et al. All-cause mortality effects of replacing sedentary time with physical activity and sleeping using an isotemporal substitution model: a prospective study of 201,129 mid-aged and older adults. International Journal of Behavioral Nutrition & Physical Activity. 2015;12:1-10.

807.	Stanley R, Jones R, Swan C, Christian H, Sherring J, Shilton T, et al. Exploring stakeholders' perceptions of the acceptability, usability, and dissemination of the australian 24-hour movement guidelines for the early years. Journal of Physical Activity and Health. 2020;17(1):120-5.

808.	Starbek P, Kastelic K, Šarabon N. The Impact of Online-Schooling during COVID-19 on Device-Measured 24-Hour Movement Behaviours among High School Students: A Compositional Data Analysis. Children. 2022;9(5):667-N.PAG.

809.	Štefelová N, Palarea-Albaladejo J, Hron K, Gába A, Dygrýn J. Compositional PLS biplot based on pivoting balances: an application to explore the association between 24-h movement behaviours and adiposity. Computational Statistics. 2024;39(2):835-63.

810.	Stenholm S, Suorsa K, Leskinen T, Myllyntausta S, Pulakka A, Pentti J, et al. Finnish Retirement and Aging Study: a prospective cohort study. BMJ Open. 2023;13(12).

811.	Stevens ML, Gupta N, Inan Eroglu E, Crowley PJ, Eroglu B, Bauman A, et al. Thigh-worn accelerometry for measuring movement and posture across the 24-hour cycle: a scoping review and expert statement. BMJ open sport & exercise medicine. 2020;6(1):e000874.

812.	Stewart T, Narayanan A, Hedayatrad L, Neville J, Mackay L, Duncan S. A dual-accelerometer system for classifying physical activity in children and adults. Medicine and Science in Sports and Exercise. 2018;50(12):2595-602.

813.	Stoner L, Higgins S, Paterson C. The 24-h activity cycle and cardiovascular outcomes: establishing biological plausibility using arterial stiffness as an intermediate outcome. American Journal of Physiology - Heart and Circulatory Physiology. 2023;325(6):H1243-H63.

814.	Su JJ, Wei EX, Clark C, Liang KX, Sun XJ. Physical exercise, Sedentaary Behaviour, Sleep and Depression Symptoms in Chinese Young Adults During the COVID-19 Pandemic: A Compositional Isotemporal Analysis. INTERNATIONAL JOURNAL OF MENTAL HEALTH PROMOTION. 2022;24(5):759-69.

815.	Suárez ÁP, Lombán BN, Soto EC, Sánchez JMP, Rodríguez LGG, Ortega RM. Weight status, body composition, and diet quality of spanish schoolchildren according to their level of adherence to the 24-hour movement guidelines. Nutricion Hospitalaria. 2021;38(1):73-84.

816.	Šuc A, Einfalt L, Šarabon N, Kastelic K. Validity and reliability of self-reported methods for assessment of 24-h movement behaviours: a systematic review. International Journal of Behavioral Nutrition & Physical Activity. 2024;21(1):1-18.

817.	Suchert V, Hanewinkel R, Neumann C, Hansen J. Regional Socioeconomic Deprivation in Germany and Adherence to the 24-h Movement Guidelines among Children and Adolescents. Children. 2023;10(8).

818.	Suherman A, Rahayu NI, Zaky M. Pattern of physical activity and sedentary behavior in early childhood. International Journal of Human Movement and Sports Sciences. 2021;9(4):125-9.

819.	Sun J, Jiang X, Wei S. Combinations of physical activity, screen time and sleep, and their association with subjective wellbeing in children. Complementary Therapies in Clinical Practice. 2023;51:N.PAG-N.PAG.

820.	Sun Q, Zhan X. Association of physical activity, screen time and sleep with depressivesymptoms in adolescents. International Journal of Mental Health Promotion. 2021;23(1):75-86.

821.	Sun W, Li J, Zhou J, Li S, Hou L, Xiao W, et al. Meeting the Canadian 24-Hour Movement Guidelines and physical-mental comorbidity among Chinese children and adolescents: Prevalence, associations, and the population impacts. Journal of psychosomatic research. 2024;176(0376333, juv):111544.

822.	Sun X, Yang L, Zhu Z, Wang W, Zhu L, Dong S, et al. Association between meeting 24-hour movement behavior guidelines and quality of life in adolescents with idiopathic scoliosis. BMC Public Health. 2024;24(1).

823.	Sun Y, Liu Y, Yin X, Li M, Zhang T, Zhang F, et al. Proportion of Chinese Children and Adolescents Meeting 24-Hour Movement Guidelines and Associations with Overweight and Obesity. International Journal of Environmental Research and Public Health. 2023;20(2):1408.

824.	Sun Y, Luo D, Guan K, Luo X. Meeting 24-h movement behavior guidelines is associated with academic engagement, social-emotional functioning in obese/overweight youth. Complementary Therapies in Clinical Practice. 2024;56:N.PAG-N.PAG.

825.	Suorsa K, Gupta N, Leskinen T, Andersen LL, Pasanen J, Hettiarachchi P, et al. Modifications of 24-h movement behaviors to prevent obesity in retirement: a natural experiment using compositional data analysis. International Journal of Obesity. 2023;47(10):922-30.

826.	Suorsa K, Leskinen T, Gupta N, Andersen LL, Pasanen J, Hettiarachchi P, et al. Longitudinal Associations between 24-h Movement Behaviors and Cardiometabolic Biomarkers: A Natural Experiment over Retirement. Medicine and science in sports and exercise. 2024;56(7):1297-306.

827.	Suorsa K, Leskinen T, Pasanen J, Pulakka A, Myllyntausta S, Pentti J, et al. Changes in the 24-h movement behaviors during the transition to retirement: compositional data analysis. International Journal of Behavioral Nutrition & Physical Activity. 2022;19(1):1-11.

828.	Swindell N, Rees P, Fogelholm M, Drummen M, MacDonald I, Martinez JA, et al. Compositional analysis of the associations between 24-h movement behaviours and cardio-metabolic risk factors in overweight and obese adults with pre-diabetes from the PREVIEW study: Cross-sectional baseline analysis. International Journal of Behavioral Nutrition and Physical Activity. 2020;17(1).

829.	Swindell N, Wachira LJ, Okoth V, Kagunda S, Owino G, Ochola S, et al. Prevalence and correlates of compliance with 24-h movement guidelines among children from urban and rural Kenya-The Kenya-LINX project. PLoS ONE. 2022;17(12 December):e0279751.

830.	Talarico R, Janssen I. Compositional associations of time spent in sleep, sedentary behavior and physical activity with obesity measures in children. International Journal of Obesity. 2018;42(8):1508-14.

831.	Tan SYX, Chia A, Tai BC, Natarajan P, Goh CMJL, Shek LP, et al. A Web-Based, Time-Use App To Assess Children's Movement Behaviors: Validation Study of My E-Diary for Activities and Lifestyle (MEDAL). JMIR pediatrics and parenting. 2022;5(2):e33312.

832.	Tan SYX, Chia A, Tai BC, Toh JY, Colega M, Padmapriya N, et al. Correlates of lifestyle patterns among children in Singapore aged 10 years: the growing up in Singapore towards healthy outcomes (GUSTO) study. BMC Public Health. 2024;24(1).

833.	Tan SYX, Padmapriya N, Bernard JY, Toh JY, Wee HL, Tan KH, et al. Cross-sectional and prospective associations between children's 24-h time use and their health-related quality of life: a compositional isotemporal substitution approach. The Lancet Regional Health - Western Pacific. 2023;41((Tan, Padmapriya, Wee, Tan, Chong, Muller-Riemenschneider) Saw Swee Hock School of Public Health, National University of Singapore, Singapore, Singapore(Padmapriya, Chong, Eriksson) Department of Obstetrics and Gynaecology, Yong Loo Lin School of Medicine):100918.

834.	Tanaka C, Okada S, Takakura M, Hasimoto K, Mezawa H, Ando D, et al. Relationship between adherence to WHO “24-Hour Movement Guidelines for the Early Years” and motor skills or cognitive function in preschool children: SUNRISE pilot study. Japanese Journal of Physical Fitness and Sports Medicine. 2020;69(4):327-33.

835.	Tanaka C, Shikano A, Imai N, Chong KH, Howard SJ, Tanabe K, et al. Accelerometer-Measured Physical Activity and Sedentary Time among Children in Japan before and during COVID-19: A Cross-Sectional and Longitudinal Analysis. International Journal of Environmental Research and Public Health. 2023;20(2).

836.	Tanaka C, Tremblay MS, Okuda M, Tanaka S. Association between 24‐hour movement guidelines and physical fitness in children. Pediatrics International. 2020;62(12):1381-7.

837.	Tanaka C, Tremblay MS, Tanaka S. Gender differences in the proportion of Japanese parents meeting 24-h movement guidelines and associations with weight status. American journal of human biology : the official journal of the Human Biology Council. 2024((Tanaka) Department of Human Nutrition, Tokyo Kasei Gakuin University, Chiyoda-ku, Tokyo, Japan(Tanaka, Tanaka) Department of Nutrition and Metabolism, National Institute of Health and Nutrition, National Institutes of Biomedical Innovation, Health and Nu):e24142.

838.	Tandon PS, Sasser T, Gonzalez ES, Whitlock KB, Christakis DA, Stein MA. Physical activity, screen time, and sleep in children with ADHD. Journal of Physical Activity and Health. 2019;16(6):416-22.

839.	Tandon S, Patte KA, Goldfield GS, Leatherdale ST, Chaput JP. Loneliness during COVID-19 and its association with eating habits and 24-hour movement behaviours in a sample of Canadian adolescents. Preventive Medicine Reports. 2023;35.

840.	Tang BQ, Chen BH, Li YY, Liu HQ, Xu SQ, Wang SM. A cross-sectional study on the relationship between 24-hour activities and depressive symptoms in vocational school students. Fudan University Journal of Medical Sciences. 2024;51(2):159-65.

841.	Tang Y, Song Y, Du Y, Cao L. Association between 24-hour activity behavior and cardiorespiratory endurance in children and adolescents based on isochronous substitution models. Zhongguo Ertong Baojian Zazhi. 2024;32(8):826-31and55.

842.	Tapia-Serrano MA, Garcia-Hermoso A, Sevil-Serrano J, Sanchez-Oliva D, Sanchez-Miguel PA. Is adherence to 24-Hour Movement Guidelines associated with a higher academic achievement among adolescent males and females? Journal of Science and Medicine in Sport. 2022;25(2):155-61.

843.	Tapia-Serrano MA, Sanchez-Oliva D, Sevil-Serrano J, Marques A, Sanchez-Miguel PA. 24-h movement behaviours in Spanish youth before and after 1-year into the covid-19 pandemic and its relationship to academic performance. Scientific reports. 2022;12(1):16660.

844.	Tapia-Serrano MA, Sevil-Serrano J, Sanchez-Miguel PA. Adherence to 24-hour movement guidelines among spanish adolescents: Differences between boys and girls. Children. 2021;8(2):95.

845.	Tapia-Serrano MA, Sevil-Serrano J, Sanchez-Miguel PA, Lopez-Gil JF, Tremblay MS, Garcia-Hermoso A. Prevalence of meeting 24-Hour Movement Guidelines from pre-school to adolescence: A systematic review and meta-analysis including 387,437 participants and 23 countries. Journal of sport and health science. 2022;11(4):427-37.

846.	Tapia-Serrano MÁ, Sevil-Serrano J, Sánchez-Oliva D, Vaquero-Solís M, Sánchez-Miguel PA. Effects of a school-based intervention on physical activity, sleep duration, screen time, and diet in children. Revista de Psicodidactica. 2022;27(1):56-65.

847.	Tapia‐Serrano MÁ, López‐Gil JF, Sevil‐Serrano J, García‐Hermoso A, Sánchez‐Miguel PA. What is the role of adherence to 24‐hour movement guidelines in relation to physical fitness components among adolescents? Scandinavian Journal of Medicine & Science in Sports. 2023;33(8):1373-83.

848.	Taylor A, Kong C, Zhang Z, Herold F, Ludyga S, Healy S, et al. Associations of meeting 24-h movement behavior guidelines with cognitive difficulty and social relationships in children and adolescents with attention deficit/hyperactive disorder. Child & Adolescent Psychiatry & Mental Health. 2023;17(1):1-17.

849.	Taylor RW, Haszard JJ, Farmer VL, Richards R, Te Morenga L, Meredith-Jones K, et al. Do differences in compositional time use explain ethnic variation in the prevalence of obesity in children? Analyses using 24-hour accelerometry. International Journal of Obesity. 2020;44(1):94-103.

850.	Taylor RW, Haszard JJ, Healey D, Meredith-Jones KA, Taylor BJ, Galland BC. Adherence to 24-h movement behavior guidelines and psychosocial functioning in young children: a longitudinal analysis. International Journal of Behavioral Nutrition & Physical Activity. 2021;18(1):1-13.

851.	Taylor RW, Haszard JJ, Meredith-Jones KA, Azeem AA, Galland BC, Heath A-LM, et al. Associations between activity, sedentary and sleep behaviours and psychosocial health in young children: a longitudinal compositional time-use study. Journal of Activity, Sedentary and Sleep Behaviors. 2023;2(1).

852.	Taylor RW, Haszard JJ, Meredith-Jones KA, Galland BC, Heath A-LM, Lawrence J, et al. 24-h movement behaviors from infancy to preschool: cross-sectional and longitudinal relationships with body composition and bone health. International Journal of Behavioral Nutrition & Physical Activity. 2018;15(1):N.PAG-N.PAG.

853.	Tennant EM, Tremblay MS, Faulkner G, Gainforth HL, Latimer-Cheung AE. Exploring Parents’ Message Receipt and Message Enactment of the World’s First Integrated Movement Behaviour Guidelines for Children and Youth. Journal of Health Communication. 2019;24(7-8):643-53.

854.	Thewlis D, Bahl JS, Fraysse F, Curness K, Arnold JB, Taylor M, et al. Objectively measured 24-hour activity profiles before and after total hip arthroplasty. Bone and Joint Journal. 2019;101 B(4):415-25.

855.	Thivel D, Tremblay MS, Katzmarzyk PT, Fogelholm M, Hu G, Maher C, et al. Associations between meeting combinations of 24-hour movement recommendations and dietary patterns of children: A 12-country study. Preventive Medicine. 2019;118:159-65.

856.	Toledo-Vargas M, Perez-Contreras P, Chandia-Poblete D, Aguilar-Farias N. Compliance of the 24-Hour Movement Guidelines in 9- to 11-Year-Old Children From a Low-Income Town in Chile. Journal of physical activity & health. 2020;17(10):1034-41.

857.	Tomasone JR, Flood SM, Latimer-Cheung AE, Faulkner G, Duggan M, Jones R, et al. Knowledge translation of the Canadian 24-Hour Movement Guidelines for Adults aged 18–64 years and Adults aged 65 years or older: a collaborative movement guideline knowledge translation process. Applied Physiology, Nutrition & Metabolism. 2020;45:S103-S24.

858.	Tomasone JR, Kauffeldt KD, Morgan TL, Magor KW, Latimer-Cheung AE, Faulkner G, et al. Dissemination and implementation of national physical activity, sedentary behaviour, and/or sleep guidelines among community-dwelling adults aged 18 years and older: a systematic scoping review and suggestions for future reporting and research. Applied physiology, nutrition, and metabolism = Physiologie appliquee, nutrition et metabolisme. 2020;45(10 Supplement 2):S258-S83.

859.	Tomaz SA, Okely AD, van Heerden A, Vilakazi K, Samuels ML, Draper CE. The South African 24-Hour Movement Guidelines for Birth to 5 Years: Results From the Stakeholder Consultation. Journal of physical activity & health. 2020;17(1):126-37.

860.	Tomaz SA, Prioreschi A, Watson ED, McVeigh JA, Rae DE, Jones RA, et al. Body mass index, physical activity, sedentary behavior, sleep, and gross motor skill proficiency in preschool children from a low- To middle-income urban setting. Journal of Physical Activity and Health. 2019;16(7):525-32.

861.	Tooth LR, Mielke GI, Moss KM. Adherence to 24-h movement behaviour guidelines in families with multiple children. Child: Care, Health and Development. 2024;50(1).

862.	Tremblay MS. Introducing 24-Hour Movement Guidelines for the Early Years: A New Paradigm Gaining Momentum. Journal of physical activity & health. 2020;17(1):92-5.

863.	Tremblay MS, Carson V, Chaput J-P. Introduction to the Canadian 24-Hour Movement Guidelines for Children and Youth: An Integration of Physical Activity, Sedentary Behaviour, and Sleep. Applied Physiology, Nutrition & Metabolism. 2016;41:iii-iv.

864.	Tremblay MS, Carson V, Chaput J-P, Connor Gorber S, Thy D, Duggan M, et al. Canadian 24-Hour Movement Guidelines for Children and Youth: An Integration of Physical Activity, Sedentary Behaviour, and Sleep. Applied Physiology, Nutrition & Metabolism. 2016;41:S311-S27.

865.	Tremblay MS, Chaput J-P, Adamo KB, Aubert S, Barnes JD, Choquette L, et al. Canadian 24-Hour Movement Guidelines for the Early Years (0-4 years): An Integration of Physical Activity, Sedentary Behaviour, and Sleep. BMC Public Health. 2017;17:1-32.

866.	Tremblay MS, Ross R. How should we move for health? The case for the 24-hour movement paradigm. Canadian Medical Association Journal (CMAJ). 2020;192(49):E1728-E9.

867.	Trost SG, Terranova CO, Brookes DSK, Chai LK, Byrne RA. Reliability and validity of rapid assessment tools for measuring 24-hour movement behaviours in children aged 0-5 years: the Movement Behaviour Questionnaire Baby (MBQ-B) and child (MBQ-C). The international journal of behavioral nutrition and physical activity. 2024;21(1):43.

868.	Tsanas A. Investigating Wrist-Based Acceleration Summary Measures across Different Sample Rates towards 24-Hour Physical Activity and Sleep Profile Assessment. Sensors. 2022;22(16).

869.	Tudor-Locke C, Leonardi C, Johnson WD, Katzmarzyk PT. Time spent in physical activity and sedentary behaviors on the working day: The American time use survey. Journal of Occupational and Environmental Medicine. 2011.

870.	Tudor-Locke C, Schuna JM, Katzmarzyk PT, Liu W, Hamrick KS, Johnson WD. Body mass index: Accounting for full time sedentary occupation and 24-Hr self-reported time use. PLoS ONE. 2014;9(10):109051.

871.	Tye LS, Scott T, Haszard JJ, Peddie MC. Physical activity, sedentary behaviour and sleep, and their association with bmi in a sample of adolescent females in New Zealand. International Journal of Environmental Research and Public Health. 2020;17(17):1-10.

872.	Tyler R, Atkin AJ, Dainty JR, Dumuid D, Fairclough SJ. Cross-sectional associations between 24-hour activity behaviours and motor competence in youth: a compositional data analysis. Journal of Activity, Sedentary and Sleep Behaviors. 2022;1(1).

873.	Uzicanin E, Gilic B, Babajic F, Huremovic T, Hodzic S, Bilalic J, et al. The international study of movement behaviours in the early years: A pilot study from Bosnia and Herzegovina. MONTENEGRIN JOURNAL OF SPORTS SCIENCE AND MEDICINE. 2024.

874.	Vale S, Mota J. Adherence to 24-hour movement guidelines among Portuguese preschool children: the prestyle study. Journal of Sports Sciences. 2020;38(18):2149-54.

875.	Vanderlinden J, Biddle GJH, Boen F, van Uffelen JGZ. Are reallocations between sedentary behaviour and physical activity associated with better sleep in adults aged 55+ years? An isotemporal substitution analysis. International Journal of Environmental Research and Public Health. 2020;17(24):1-14.

876.	Vanderlinden J, Biddle GJH, Boen F, van Uffelen JGZ. To be well or not to be well: compositional associations of physical activity, sedentary behaviour and sleep with mental well-being in Flemish adults aged 55+ years. Journal of Activity, Sedentary and Sleep Behaviors. 2023;2(1).

877.	Vanderloo LM, Maguire JL, Keown-Stoneman CDG, Parkin PC, Borkhoff CM, Tremblay MS, et al. Associations Between Meeting the 24-Hour Movement Guidelines and Cardiometabolic Risk in Young Children. Pediatric Exercise Science. 2021;33(3):112-9.

878.	Vanderloo LM, Omand J, Keown-Stoneman CDG, Janus M, Tremblay MS, Maguire JL, et al. Association Between Physical Activity, Screen Time and Sleep, and School Readiness in Canadian Children Aged 4 to 6 Years. Journal of Developmental & Behavioral Pediatrics. 2022;43(2):96-103.

879.	Vaquero-Solís M, Tapia-Serrano MA, Hortigüela-Alcalá D, Jacob-Sierra M, Sánchez-Miguel PA. Health promotion through movement behaviors and its relationship with quality of life in spanish high school adolescents: A predictive study. International Journal of Environmental Research and Public Health. 2021;18(14).

880.	Verhoog S, Braun KVE, Bano A, van Rooij FJA, Franco OH, Koolhaas CM, et al. Associations of Activity and Sleep With Quality of Life: A Compositional Data Analysis. American Journal of Preventive Medicine. 2020;59(3):412-9.

881.	Verschuren O, Hulst RY, Voorman J, Pillen S, Luitwieler N, Dudink J, et al. 24-hour activity for children with cerebral palsy: a clinical practice guide. Developmental Medicine and Child Neurology. 2021;63(1):54-9.

882.	Verswijveren SJJM, Dingle S, Donnelly AE, Dowd KP, Ridgers ND, Carson BP, et al. How are different clusters of physical activity, sedentary, sleep, smoking, alcohol, and dietary behaviors associated with cardiometabolic health in older adults? A cross-sectional latent class analysis. Journal of Activity, Sedentary and Sleep Behaviors. 2023;2(1).

883.	Viana RS, Nascimento-Ferreira MV, Schaan BD, Bloch KV, de Carvalho KMB, Cureau FV, et al. Prevalence of the Double Burden of Malnutrition among Adolescents: Associations with Lifestyle Behaviors and Clusters of Social Determinants. Children. 2024;11(6):620.

884.	Vidmar AP, Cáceres NA, Schneider-Worthington CR, Shirazipour C, Buman MP, de la Haye K, et al. Integration of Time-Based Recommendations with Current Pediatric Health Behavior Guidelines: Implications for Obesity Prevention and Treatment in Youth. Current Obesity Reports. 2022;11(4):236-53.

885.	Virgara R, Phillips A, Lewis L, Richardson M, Maher C. Development of Australian physical activity and screen time guidelines for outside school hours care: an international Delphi study. International Journal of Behavioral Nutrition and Physical Activity. 2021;18(1).

886.	Wahlström V, Januario LB, Mathiassen SE, Heiden M, Hallman DM. Hybrid office work in women and men: do directly measured physical behaviors differ between days working from home and days working at the office? Annals of Work Exposures and Health. 2023;67(9):1043-55.

887.	Walmsley R, Chan S, Smith-Byrne K, Ramakrishnan R, Woodward M, Rahimi K, et al. Reallocation of time between device-measured movement behaviours and risk of incident cardiovascular disease. British Journal of Sports Medicine. 2022;56(18):1008-17.

888.	Walsh JJ, Barnes JD, Cameron JD, Goldfield GS, Chaput J-P, Gunnell KE, et al. Associations between 24 hour movement behaviours and global cognition in US children: a cross-sectional observational study. The Lancet Child & adolescent health. 2018;2(11):783-91.

889.	Walters AJ, Lithopoulos A, Tennant EM, Weissman S, Latimer-Cheung AE. Exploring attention to the Canadian 24-Hour Movement Guidelines for Children and Youth using eye-tracking: A randomized control trial. Public Health Nursing. 2022;39(5):982-92.

890.	Walters AJ, Tomasone JR, Latimer-Cheung AE. An Experimental Test of a Generic Messaging Approach for the Canadian 24-Hour Movement Guidelines for Adults. Journal of health communication. 2022;27(1):8-16.

891.	Wang D, Lyu H, Li T. Characteristics and enlightenment of Guidelines on Physical Activity Sedentary Behavior and Sleep for Children under 5 Years of Age. Chinese Journal of School Health. 2023;44(5):645-8.

892.	Wang H, Liu C, Chen H. Effects of comprehensive intervention on 24 h activity behavior among obese students in vocational colleges. Chinese Journal of School Health. 2024;45(2):207-12.

893.	Wang J, Qi Y, Xin Z, Huo Y, Wan Q, Qin Y, et al. Isotemporal substitution of different behaviour patterns with the presence of MAFLD in Chinese adults. Liver International. 2022;42(12):2683-95.

894.	Wang J, Zhao L, Guan H, Gao Q, Liang J, He S, et al. The mediating effect of 24-h time-use behaviors on the relationship between depression and mortality: A compositional mediation analysis for survival outcomes. Journal of Affective Disorders. 2024;350:222-9.

895.	Wang R, Wu B, Gao Y, Li H, Zhang T. Relationship between 24￣hour movement behavior and obesity indicators in children and adolescents. Chinese Journal of School Health. 2023;44(1):28-31.

896.	Wang S, Intille S, Ponnada A, Do B, Rothman A, Dunton G. Investigating Microtemporal Processes Underlying Health Behavior Adoption and Maintenance: Protocol for an Intensive Longitudinal Observational Study. JMIR Research Protocols. 2022;11(7).

897.	Wang S, Liang W, Song H, Su N, Zhou L, Duan Y, et al. Prospective association between 24-hour movement behaviors and mental health among overweight/obese college students: a compositional data analysis approach. Frontiers in public health. 2023;11((Wang) School of Economics and Business Administration, Chongqing University of Education, Chongqing, China(Wang) Department of Military Medical Geography, Army Medical Service Training Base, Army Medical University, Chongqing, China(Liang, Su) School of):1203840.

898.	Wang W, Haegele JA, Wu Y, Li C. Meeting the 24-Hour Movement Guidelines and Outcomes in Adolescents with ADHD: A Cross-Sectional Observational Study. International Journal of Environmental Research and Public Health. 2022;19(4):2132.

899.	Wang X, Huang Y, Meng N, Zhang J, Wu Q, Zhang Y. Suboptimal movement behaviours among children under two years old in early childhood education institutions in urban China: A cross-sectional study. Journal of Global Health. 2023;13.

900.	Wang X, Ye P, Qiao T. Associations between adherence to 24-hour movement guidelines and socio-emotional competence among preschool children: A cross-sectional study. Complementary Therapies in Clinical Practice. 2024;57.

901.	Wang Y, Zhang P, Wang M, Gong Q, Yu C, Wang H, et al. Isotemporal Substitution Effects of Daily Time Use on Cardiorespiratory Fitness of Children in the OptiChild Study: A Mediation Analysis with Diet Quality. Nutrients. 2024;16(16):2788.

902.	Wang Y, Zhu R, Long J, Zhang Y. Levels of 24-hour movement and associations with childhood obesity in Chinese school students. Chinese Journal of School Health. 2021;42(4):606-10.

903.	Watson A, Dumuid D, Maher C, Olds T. Associations between meeting 24-hour movement guidelines and academic achievement in Australian primary school-aged children. Journal of sport and health science. 2022;11(4):521-9.

904.	Watson A, Dumuid D, Olds T. Associations Between 24-Hour Time Use and Academic Achievement in Australian Primary School–Aged Children. Health Education & Behavior. 2020;47(6):905-13.

905.	Weatherson K, Gierc M, Patte K, Qian W, Leatherdale S, Faulkner G. Complete mental health status and associations with physical activity, screen time, and sleep in youth. Mental Health and Physical Activity. 2020;19.

906.	Weatherson KA, Joopally H, Wunderlich K, Kwan MY, Tomasone JR, Faulkner G. Post-secondary students' adherence to the Canadian 24-Hour Movement Guidelines for Adults: Results from the first deployment of the Canadian Campus Wellbeing Survey (CCWS). Health promotion and chronic disease prevention in Canada : research, policy and practice. 2021;41(6):173-81.

907.	Weaver RG, Hensing C, Armstrong B, Adams EL, Beets M. Seasonal Shifts in Children's Sedentary Behaviors, Physical Activity, and Sleep: A Systematic Review and meta-Analysis. Pediatric clinics of North America. 2022;69(4):671-93.

908.	Wenden EJ, Virgara R, Pearce N, Budgeon C, Christian HE. Movement behavior policies in the early childhood education and care setting: An international scoping review. Frontiers in public health. 2023;11(101616579):1077977.

909.	Wendt A, Bielemann RM, Wehrmeister FC, Ricardo LIC, Muller WdA, Machado AKF, et al. Is rest-activity rhythm prospectively associated with all-cause mortality in older people regardless of sleep and physical activity level? The 'Como Vai?' Cohort study. PloS one. 2024;19(2):e0298031.

910.	Whitaker KM, Jones MA, Smith K, Catov J, Feghali M, Kline CE, et al. Study Design and Protocol of the Multisite Pregnancy 24/7 Cohort Study. American Journal of Epidemiology. 2024;193(3):415-25.

911.	Whiting S, Buoncristiano M, Gelius P, Abu-Omar K, Pattison M, Hyska J, et al. Physical Activity, Screen Time, and Sleep Duration of Children Aged 6-9 Years in 25 Countries: An Analysis within the WHO European Childhood Obesity Surveillance Initiative (COSI) 2015-2017. Obesity Facts. 2021;14(1):32-44.

912.	Wilhite K, Booker B, Huang B-H, Antczak D, Corbett L, Parker P, et al. Combinations of Physical Activity, Sedentary Behavior, and Sleep Duration and Their Associations With Physical, Psychological, and Educational Outcomes in Children and Adolescents: A Systematic Review. American Journal of Epidemiology. 2023;192(4):665-79.

913.	Wilhite K, del Pozo Cruz B, Noetel M, Lonsdale C, Ridgers ND, Maher C, et al. Socioeconomic position as a predictor of youth's movement trajectory profiles between ages 10 and 14 years. International Journal of Behavioral Nutrition & Physical Activity. 2023;20(1):1-14.

914.	Willems I, Verbestel V, Calders P, Lapauw B, De Craemer M. Test-Retest Reliability and Internal Consistency of a Newly Developed Questionnaire to Assess Explanatory Variables of 24-h Movement Behaviors in Adults. International Journal of Environmental Research and Public Health. 2023;20(5):4407.

915.	Willems I, Verbestel V, Dumuid D, Stanford TE, Calders P, Lapauw B, et al. Cross-sectional associations between 24-hour movement behaviors and cardiometabolic health among adults with type 2 diabetes mellitus: A comparison according to weight status. Journal of Science and Medicine in Sport. 2024;27(3):179-86.

916.	Williams JS, Dunford EC, Cheng JL, Moncion K, Valentino SE, Droog CA, et al. The impact of the 24-h movement spectrum on vascular remodeling in older men and women: A review. American Journal of Physiology - Heart and Circulatory Physiology. 2021;320(3):H1136-H55.

917.	Williams SM, Farmer VL, Taylor BJ, Taylor RW. Do more active children sleep more? A repeated cross-sectional analysis using accelerometry. PLoS ONE. 2014;9(4).

918.	Willumsen J, Bull F. Development of WHO guidelines on physical activity, sedentary behavior, and sleep for children less than 5 years of age. Journal of Physical Activity and Health. 2020;17(1):96-100.

919.	Wong CKH, Wong RS, Cheung JPY, Tung KTS, Yam JCS, Rich M, et al. Impact of sleep duration, physical activity, and screen time on health-related quality of life in children and adolescents. Health and Quality of Life Outcomes. 2021;19(1).

920.	Wong M, Lycett K, Olds T, Gold L, Wake M. Use of time and adolescent health-related quality of life/well-being: a scoping review. Acta Paediatrica, International Journal of Paediatrics. 2017;106(8):1239-45.

921.	Wu M, Xiao J, Li F, Hu R, Liu C. 24-hour Movement Behaviors: a New Direction for the Study of Factors Affecting Health of the Elderly. Chinese General Practice. 2024;27(15):1911-6.

922.	Wu Q, Wang X, Zhang J, Zhang Y, van Velthoven MH. The effectiveness of a WeChat-based self-assessment with a tailored feedback report on improving complementary feeding and movement behaviour of children aged 6–20 months in rural China: a cluster randomized controlled trial. The Lancet Regional Health - Western Pacific. 2023;37.

923.	Wu Y, Rosenberg DE, Greenwood-Hickman MA, McCurry SM, Proust-Lima C, Nelson JC, et al. Analysis of the 24-Hour Activity Cycle: An illustration examining the association with cognitive function in the Adult Changes in Thought (ACT) Study. 2023.

924.	Xavier JFDS, Feuerstein SC, De Moraes ACF, de Oliveira TA, da Silva Gomes ER, de Almeida Silva MIA, et al. Development of a User-Friendly Self-Screening Tool for Assessing Metabolic Syndrome Risk in Youths from Economically Challenged Regions. Journal of Personalized Medicine. 2024;14(8).

925.	Xiao Q, Lu J, Zeitzer JM, Matthews CE, Saint-Maurice PF, Bauer C. Rest-activity profiles among U.S. adults in a nationally representative sample: a functional principal component analysis. International Journal of Behavioral Nutrition & Physical Activity. 2022;19(1):1-13.

926.	Xiaogang Z, Ahmad NS, Khairani AZ, Yongqian C. Association between meeting the 24-hour movement guideline and anxiety status in Chinese school-aged adolescents. Environment and Social Psychology. 2024;9(6).

927.	Xiong X, Dalziel K, Carvalho N, Xu R, Huang L. Association between 24-hour movement behaviors and health-related quality of life in children. Quality of life research : an international journal of quality of life aspects of treatment, care and rehabilitation. 2022;31(1):231-40.

928.	Xu MX, Wang XJ, Wang B, Jiang YY, Zhu ZH, Zhang T, et al. Status of Physical Activity Outside Kindergarten Among Beijing Urban PresChoolers as Well as Family and Community Environment. Zhongguo Ertong Baojian Zazhi. 2022;30(1):30-3.

929.	Xu WH, Qi J. Meeting 24-hour movement guidelines: Their relationships with overweight and obesity among Chinese children with autism spectrum disorder. RESEARCH IN AUTISM SPECTRUM DISORDERS. 2022;99.

930.	Xu WH, Wang LJ. Adherence to 24-h movement guidelines among Chinese children and adolescents with intellectual disabilities. Journal of Intellectual Disability Research. 2023;67(7):668-78.

931.	Yang Y, Yuan S, Liu Q, Li F, Dong Y, Dong B, et al. Meeting 24-Hour Movement and Dietary Guidelines: Prevalence, Correlates and Association with Weight Status among Children and Adolescents: A National Cross-Sectional Study in China. Nutrients. 2022;14(14):N.PAG-N.PAG.

932.	Yang Z, Li X, Song W, Zhang Y. Associations between meeting 24-h movement guidelines and sarcopenia risk among adults aged >= 55 years in five low- and middle-income countries. Complementary therapies in clinical practice. 2024;57((Yang, Zhang) School of Humanities and Social Science, The Chinese University of Hong Kong, Shenzhen, 518172, China; Physical Activity and Health Promotion Laboratory, The Chinese University of Hong Kong, Shenzhen, 518172, China(Li) Department of Physical):101887.

933.	Yanping QIU, Lijuan W, Yulan Z, Huan C, Guo L. The association between 24h movement behaviors and fundamental motor skills of children based on compositional data analyses. (English). Journal of Physical Education / Tiyu Xuekan. 2023;30(1):137-44.

934.	Yin L, Li F, Liu P, Yin Z, Yang Z, Pi L, et al. Examining the relationship between meeting 24-hour movement behaviour guidelines and mental health in Chinese preschool children. Frontiers in Pediatrics. 2024;12((Yin) College of Physical Education, Hunan Normal University, Changsha, China(Li) School of Physical Education, Hunan First Normal University, Changsha, China(Liu) School of Physical Education, Hunan University of Technology, Zhuzhou, China(Yin) English C):1337158.

935.	Ying L, Zhu X, Haegele J, Wen Y. Movement in High School: Proportion of Chinese Adolescents Meeting 24-Hour Movement Guidelines. International journal of environmental research and public health. 2020;17(7).

936.	You Y. Accelerometer-measured physical activity and sedentary behaviour are associated with C-reactive protein in US adults who get insufficient sleep: A threshold and isotemporal substitution effect analysis. Journal of Sports Sciences. 2024;42(6):527-36.

937.	Yu C, Zhang B, Au C, Lo F, Sun F. Physical Activity, Sedentary Behaviour, and Sleep Habits in Kindergarten Children in Hong Kong. Hong Kong Journal of Paediatrics. 2023;28(4):219-27.

938.	Yu H, Song Y, Wang Y, Wang X, Li H, Feng X, et al. The Impact of Temperature on 24-Hour Movement Behaviors among Chinese Freshmen Students. International Journal of Environmental Research and Public Health. 2023;20(6):4970.

939.	Yuan W, Zhang Y, Yang T, Cai S, Chen Z, Song X, et al. Association Between Combinations of 24-Hour Movement Behaviors and Depression Among Adolescents — Inner Mongolia Autonomous Region, China, 2019–2021. China CDC Weekly. 2023;5(40):889-93.

940.	Zahran S, Janssen I. Compositional associations of time spent in sleep, screen time, and physical activity with polysubstance use in adolescents. Addictive Behaviors. 2023;144:N.PAG-N.PAG.

941.	Zahran S, Visser C, Ross-White A, Janssen I. A systematic review of compositional analysis studies examining the associations between sleep, sedentary behaviour, and physical activity with health indicators in early childhood. Journal of Activity, Sedentary and Sleep Behaviors. 2023;2(1).

942.	Zarghani NH, Jafari Z, Amini F, Marashi SZ, Ghaffarifar S, Ghofranipour F, et al. International study of 24‐h movement behaviors of the early years (SUNRISE): A pilot study from Iran. Child: Care, Health & Development. 2024;50(3):1-9.

943.	Zawada SJ, Ganjizadeh A, Conte GM, Demaerschalk BM, Erickson BJ. Accelerometer-Measured Behavior Patterns in Incident Cerebrovascular Disease: Insights for Preventative Monitoring From the UK Biobank. Journal of the American Heart Association. 2024;13(11).

944.	Zeng X, Cai L, Yang W, Tan W, Huang W, Chen Y. Association between the 24-hour movement guidelines and executive function among Chinese children. BMC public health. 2022;22(1):1017.

945.	Zhang D, Chen S, Lopez-Gil JF, Hong J, Wang F, Liu Y. 24-Hour movement behaviours research during the COVID-19 pandemic: a systematic scoping review. BMC public health. 2023;23(1):2188.

946.	Zhang H, Sheng J, Yue X, Lou H, Ban X, Wu W, et al. Proportion, Correlates and the Associations of Meeting 24-h Movement Guidelines With Mental Well-Being and Psychological Distress: A Cross-Sectional Study of 10 589 Chinese Students. Child: Care, Health and Development. 2024;50(5).

947.	Zhang H, Zhang T, Wu B, Gao Y, Li H. Relationship between 24-hour movement behavior and physical fitness among pupils. Chinese Journal of School Health. 2023;44(1):17-22.

948.	Zhang J, Luo D, Yan X, Ma N, Liu Y, Dang J, et al. Development of core recommendations on physical activity for Chinese students aged 7-18 years based on Delphi method and analytic hierarchy process. Chinese Journal of School Health. 2021;42(7):1014-9.

949.	Zhang R, Zhang C-Q, Gu D. Prospective associations between adherence to 24-hour movement guidelines and mental well-being in Chinese adolescents. Journal of Sports Sciences. 2023;41(19):1735-43.

950.	Zhang R, Zhang CQ, Gu D. Integrating perceived physical environments and the theory of planned behaviors when explaining adherence to 24‐hour movement guidelines in Chinese adolescents. Scandinavian Journal of Medicine & Science in Sports. 2024;34(1):1-10.

951.	Zhang T, Li H, Li C, Zhang L, Zhang Z. The Compositional Impacts of 2 Distinct 24-Hour Movement Behavior Change Patterns on Physical Fitness in Chinese Adolescents. Journal of physical activity & health. 2022;19(4):284-91.

952.	Zhang T, Li H, Zhang Z, Gao Y. A systematic review of the association between 24-hour movement behavior and obesity in children and adolescents. Chinese Journal of School Health. 2023;44(1):23-7.

953.	Zhang X, Gu X. Adherence to the 24-hour Movement Behavior Guidelines and Associations with Depressive Symptoms among College Students. International Journal of Kinesiology in Higher Education. 2022;6(4):225-37.

954.	Zhang X, Mao F, Wu L, Zhang G, Huang Y, Chen Q, et al. Associations of physical activity, sedentary behavior and sleep duration with anxiety symptoms during pregnancy: An isotemporal substitution model. Journal of Affective Disorders. 2022;300:137-44.

955.	Zhang Y, Chi X, Huang L, Yang X, Chen S. Cross-sectional association between 24-hour movement guidelines and depressive symptoms in Chinese university students. PeerJ. 2024;12(4):e17217.

956.	Zhang Y, Kuang J, Luo X, Zhao M, Liu X. Association between Meeting 24-Hour Movement Guidelines and Psychological Features of Chinese Emerging Adults. International Journal of Mental Health Promotion. 2024;26(5):399-406.

957.	Zhang Y, Pan Y, Ma Z, Wang D, Zou R, Fan F. Cross-sectional and longitudinal associations of adherence to the 24-hour movement guidelines with mental health problems among Chinese adolescents. Journal of psychosomatic research. 2023;170(0376333, juv):111352.

958.	Zhang Y, Wang Q, Zhu Z, Zong Q, Wu X, Tao F. The link between unhealthy lifestyle behaviors and emotional and behavioral problems in children and adolescents: a latent class analysis. Psychology, Health and Medicine. 2023;28(8):2182-97.

959.	Zhang Y, Yang X, Yang Z, Chi X, Chen S. Associations of 24-hour movement guidelines adherence with fruit and vegetable intake in university students. PeerJ. 2024;12(8):e17875.

960.	Zhang Z, Pereira JR, Sousa-Sá E, Okely AD, Feng X, Santos R. Environmental characteristics of early childhood education and care, daily movement behaviours and adiposity in toddlers: A multilevel mediation analysis from the GET UP! Study. Health and Place. 2018;54:236-43.

961.	Zhang Z, Predy M, Hesketh KD, Pritchard L, Carson V. Demographic Correlates of Movement Behaviors in Infants: A Longitudinal Study. Journal of physical activity & health. 2022;19(3):177-85.

962.	Zhang Z, Sousa-Sá E, Pereira J, Chaput JP, Okely A, Feng X, et al. Correlates of nocturnal sleep duration, nocturnal sleep variability, and nocturnal sleep problems in toddlers: results from the GET UP! Study. Sleep Medicine. 2019;53:124-32.

963.	Zhao H, Wu N, Haapala EA, Gao Y. Association between meeting 24-h movement guidelines and health in children and adolescents aged 5–17 years: a systematic review and meta-analysis. Frontiers in Public Health. 2024;12.

964.	Zhao J, Mackay L, Chang K, Mavoa S, Stewart T, Ikeda E, et al. Visualising combined time use patterns of children's activities and their association with weight status and neighbourhood context. International Journal of Environmental Research and Public Health. 2019;16(5):897.

965.	Zhao M, You Y, Gao X, Li L, Li J, Cao M. The effects of a web-based 24-hour movement behavior lifestyle education program on mental health and psychological well-being in parents of children with autism spectrum disorder: A randomized controlled trial. Complementary Therapies in Clinical Practice. 2024;56.

966.	Zhao M, Zhang Y, Herold F, Chen J, Hou M, Zhang Z, et al. Associations between meeting 24-hour movement guidelines and myopia among school-aged children: A cross-sectional study. Complementary Therapies in Clinical Practice. 2023;53:N.PAG-N.PAG.

967.	Zhao MX, Hou MJ, Herold F, Chen YX, Werneck AO, Kramer AF, et al. Associations of meeting 24-hour movement behavior guidelines with social and emotional function in youth with ASD/ADHD. JOURNAL OF AFFECTIVE DISORDERS. 2024;359:189-95.

968.	Zhao T, Liu R, Han Q, Han X, Ren J, Mao M, et al. Associations of 24-hour movement behaviors with depressive symptoms in rural-dwelling older adults: a compositional data analysis. Aging Clinical & Experimental Research. 2024;36(1):1-11.

969.	Zheng J, Tan TC, Zheng K, Huang T. Development of a 24-hour movement behaviors questionnaire (24HMBQ) for Chinese college students: validity and reliability testing. BMC public health. 2023;23(1):752.

970.	Zhou L, Liang W, He Y, Duan Y, Rhodes RE, Liu H, et al. Relationship of 24-Hour Movement Behaviors with Weight Status and Body Composition in Chinese Primary School Children: A Cross-Sectional Study. International Journal of Environmental Research and Public Health. 2022;19(14):8586.

971.	Zhu JH, Shen ZZ, Liu BP, Jia CX. Replacement of sedentary behavior with various physical activities and the risk of incident depression: a prospective analysis of accelerator-measured and self-reported UK Biobank data. Social Psychiatry and Psychiatric Epidemiology. 2024.

972.	Zhu N, Guo H, Ma D, Wang Q, Ma J, Kim H. The Association between 24 h Movement Guidelines and Internalising and Externalising Behaviour Problems among Chinese Preschool Children. Children. 2023;10(7).

973.	Zhu X, Haegele JA, Healy S. Movement and mental health: Behavioral correlates of anxiety and depression among children of 6–17 years old in the U.S. Mental Health and Physical Activity. 2019;16:60-5.

974.	Zhu X, Healy S, Haegele JA, Patterson F. Twenty-Four-Hour Movement Guidelines and Body Weight in Youth. The Journal of pediatrics. 2020;218(jlz, 0375410):204-9.

975.	Zimmerman SP, Nowland L, Zhu X, Haegele JA, Ross SM. Associations between 24-h movement guidelines compliance and anxiety and depression among youth receiving special education services in the US. Disability and Health Journal. 2024;17(1).

976.	Zink J, Booker R, Wolff-Hughes DL, Allen NB, Carnethon MR, Alexandria SJ, et al. Longitudinal associations of screen time, physical activity, and sleep duration with body mass index in U.S. youth. The international journal of behavioral nutrition and physical activity. 2024;21(1):35.

977.	Zisis E, Hakimi S, Lee E-Y. Climate change, 24-hour movement behaviors, and health: a mini umbrella review. Global Health Research & Policy. 2021;6(1):1-14.

978.	胥祉涵, 王世强, 余澳林, 陈昱欣, 肖顺英, 马梓桐. 24 小时活动行为对学龄前儿童执行功能影响的 成分等时替代效益研究. (Chinese). Journal of Physical Education / Tiyu Xuekan. 2024;31(2):148-54.

979.	阎海, 朱为模. 第64届美国运动医学会年会关于体育社会学 研究趋势与热点议题. (Chinese). Journal of Beijing Sport University. 2017;40(8):53-7.

(1-250)(251-500)(501-750)(751-979)
