# Supplementary material for: Terminology for describing the 24-h cycle of physical activity, sedentary behaviour, and sleep: a scoping review
Source: Int J Behav Nutr Phys Act. 2026 Aug 5;23:107. doi: 10.1186/s12966-026-01945-6 (PMC13729492; doi:10.1186/s12966-026-01945-6)
Supplement: Supplementary file 3 [file 12966_2026_1945_MOESM3_ESM.docx]

**Supplementary Material S3.** Frequencies of study designs, country where research was conducted, health status of sample, and outcomes measured.

| **Study designs (N=979)** | **Articles reporting** | **% of total articles (n/979)** |
| --- | --- | --- |
| Cross-sectional | 629 | 64.2 |
| Longitudinal (e.g., case-control, cohort studies) | 122 | 12.5 |
| Review | 94 | 9.6 |
| Commentary or editorial | 33 | 3.4 |
| Protocol | 24 | 2.5 |
| Cross-sectional and longitudinal | 19 | 1.9 |
| Randomized Controlled Trials | 18 | 1.8 |
| Measurement studies | 14 | 1.4 |
| Expert consensus | 8 | 0.8 |
| Guideline development | 8 | 0.8 |
| Quasi-experimental | 3 | 0.3 |
| Knowledge translation | 1 | 0.1 |
| Policy analysis | 1 | 0.1 |
| Other | 5 | 0.5 |
| **Countries/Regions of research team (N=979)** | **Articles reporting** | **% of total articles (n/979)** |
| Australia | 261 | 26.7 |
| Canada | 246 | 25.1 |
| United States of America | 229 | 23.4 |
| China | 194 | 19.8 |
| United Kingdom | 148 | 15.1 |
| Brazil | 78 | 8.0 |
| Spain | 71 | 7.3 |
| Finland | 47 | 4.8 |
| Belgium | 44 | 4.5 |
| Portugal | 42 | 4.3 |
| Germany | 36 | 3.7 |
| Sweden | 33 | 3.4 |
| Denmark | 31 | 3.2 |
| New Zealand | 30 | 3.1 |
| Netherlands | 27 | 2.8 |
| Singapore | 25 | 2.6 |
| Czech Republic | 23 | 2.3 |
| Japan | 22 | 2.2 |
| South Africa | 22 | 2.2 |
| France | 21 | 2.1 |
| Norway | 21 | 2.1 |
| Chile | 20 | 2.0 |
| Ireland | 20 | 2.0 |
| Switzerland | 19 | 1.9 |
| Ecuador | 14 | 1.4 |
| Colombia | 12 | 1.2 |
| South Korea | 12 | 1.2 |
| India | 11 | 1.1 |
| Slovenia | 11 | 1.1 |
| Malaysia | 8 | 0.8 |
| Austria | 7 | 0.7 |
| Italy | 7 | 0.7 |
| Mexico | 6 | 0.6 |
| Saudi Arabia | 6 | 0.6 |
| Thailand | 6 | 0.6 |
| Bangladesh | 5 | 0.5 |
| Croatia | 5 | 0.5 |
| Greece | 5 | 0.5 |
| Israel | 5 | 0.5 |
| Poland | 5 | 0.5 |
| Scotland | 5 | 0.5 |
| Indonesia | 4 | 0.4 |
| Uruguay | 4 | 0.4 |
| Bulgaria | 3 | 0.3 |
| Hong Kong | 3 | 0.3 |
| Kenya | 3 | 0.3 |
| Morocco | 3 | 0.3 |
| Philippines | 3 | 0.3 |
| Sri Lanka | 3 | 0.3 |
| Argentina | 2 | 0.2 |
| Bosnia and Herz. | 2 | 0.2 |
| Costa Rica | 2 | 0.2 |
| Egypt | 2 | 0.2 |
| Ethiopia | 2 | 0.2 |
| Fiji | 2 | 0.2 |
| Hungary | 2 | 0.2 |
| Iran | 2 | 0.2 |
| Kyrgyzstan | 2 | 0.2 |
| Lithuania | 2 | 0.2 |
| Luxembourg | 2 | 0.2 |
| Mongolia | 2 | 0.2 |
| Mozambique | 2 | 0.2 |
| Peru | 2 | 0.2 |
| Romania | 2 | 0.2 |
| Russia | 2 | 0.2 |
| Taiwan | 2 | 0.2 |
| Turkey | 2 | 0.2 |
| United Arab Emirates | 2 | 0.2 |
| Venezuela | 2 | 0.2 |
| Vietnam | 2 | 0.2 |
| Zimbabwe | 2 | 0.2 |
| Botswana | 1 | 0.1 |
| Cyprus | 1 | 0.1 |
| Estonia | 1 | 0.1 |
| Jordan | 1 | 0.1 |
| Kazakhstan | 1 | 0.1 |
| Latvia | 1 | 0.1 |
| Macedonia | 1 | 0.1 |
| Malawi | 1 | 0.1 |
| Malta | 1 | 0.1 |
| Montenegro | 1 | 0.1 |
| Nigeria | 1 | 0.1 |
| Pakistan | 1 | 0.1 |
| Papua New Guinea | 1 | 0.1 |
| San Marino | 1 | 0.1 |
| Serbia | 1 | 0.1 |
| Slovakia | 1 | 0.1 |
| Tajikistan | 1 | 0.1 |
| Tanzania | 1 | 0.1 |
| Tunisia | 1 | 0.1 |
| Turkemistan | 1 | 0.1 |
| Uganda | 1 | 0.1 |
| **Health status reported (N=979)** | **Articles reporting** | **% of total articles (n/979)** |
| Not reported or not applicable | 754 | 77.0 |
| Healthy | 83 | 8.5 |
| Overweight/obese | 24 | 2.5 |
| Autism spectrum disorder | 14 | 1.4 |
| Physical disabilities | 14 | 1.4 |
| Pregnant women | 12 | 1.2 |
| Cancer | 8 | 0.8 |
| Visual impairment | 8 | 0.8 |
| Type 2 diabetes | 7 | 0.7 |
| Cardiovascular disease | 6 | 0.6 |
| Cerebral palsy | 6 | 0.6 |
| Attention deficit and hyperactivity disorder | 4 | 0.4 |
| Chronic diseases | 4 | 0.4 |
| Chronic obstructive pulmonary disease | 4 | 0.4 |
| Cognitive impairment | 4 | 0.4 |
| Stroke | 4 | 0.4 |
| Type 1 diabetes | 4 | 0.4 |
| Cystic fibrosis | 3 | 0.3 |
| Rheumatoid arthritis | 3 | 0.3 |
| Other | 69 | 7.1 |
| **Physical outcomes reported (N=979)** | **Articles reporting** | **% of total articles (n/979)** |
| No physical outcome reported | 623 | 63.6 |
| Body mass index | 227 | 23.2 |
| Adiposity | 226 | 23.1 |
| Weight | 226 | 23.1 |
| Cardiometabolic marker | 86 | 8.8 |
| Fitness | 53 | 5.4 |
| Motor skills | 36 | 3.7 |
| Bone health | 17 | 1.7 |
| Mortality | 13 | 1.3 |
| Mobility | 7 | 0.7 |
| Self-rated health | 7 | 0.7 |
| Metabolic syndrome | 6 | 0.6 |
| Body composition | 4 | 0.4 |
| Gait | 4 | 0.4 |
| Pain | 4 | 0.4 |
| Other | 101 | 10.3 |
| **Psychosocial or brain health-related outcomes reported (N=979)** | **Articles reporting** | **% of total articles (n/979)** |
| No psychosocial or brain health-related outcome reported | 694 | 70.9 |
| Depression | 85 | 8.7 |
| Cognition | 65 | 6.6 |
| Anxiety | 46 | 4.7 |
| Quality of life | 46 | 4.7 |
| Emotional health | 45 | 4.6 |
| Academic achievement/performance | 32 | 3.3 |
| Well-being | 28 | 2.9 |
| Mental health | 25 | 2.6 |
| Stress | 23 | 2.4 |
| Prosocial behaviour | 16 | 1.6 |
| Attention deficit and hyperactivity symptoms | 14 | 1.4 |
| Behavioural conduct | 12 | 1.2 |
| Psychological health | 10 | 1.0 |
| Self-esteem | 9 | 0.9 |
| Psychosocial health | 8 | 0.8 |
| Brain health | 7 | 0.7 |
| Flourishing | 6 | 0.6 |
| Suicidal ideation | 6 | 0.6 |
| Self-efficacy | 5 | 0.5 |
| Social health | 5 | 0.5 |
| Social support | 4 | 0.4 |
| Other | 88 | 9.0 |
| **Behavioural outcomes reported (N=979)** | **Articles reporting** | **% of total articles (n/979)** |
| No behavioural outcome reported | 929 | 94.9 |
| Diet | 42 | 4.3 |
| Tobacco consumption | 13 | 1.3 |
| Alcohol consumption | 11 | 1.1 |
| Substance use | 8 | 0.8 |
| Physical restraint | 1 | 0.1 |
| Reading time | 1 | 0.1 |
| Sexual activity | 1 | 0.1 |
