# Supplementary material for: Terminology for describing the 24-h cycle of physical activity, sedentary behaviour, and sleep: a scoping review
Source: Int J Behav Nutr Phys Act. 2026 Aug 5;23:107. doi: 10.1186/s12966-026-01945-6 (PMC13729492; doi:10.1186/s12966-026-01945-6)
Supplement: Supplementary file 4 [file 12966_2026_1945_MOESM4_ESM.docx]

**Supplementary Material S4.** Absolute and relative frequencies of terminology used for discussing physical activity, sedentary behaviour, or sleep as individual behaviours.

| **Physical activity terminology** | **n** | **%** |
| --- | --- | --- |
| Physical activity | 979 | 100.00 |
| Exercise | 132 | 13.48 |
| Physical exercise | 19 | 1.94 |
| Outdoor time | 18 | 1.84 |
| Walking | 18 | 1.84 |
| Playtime | 15 | 1.53 |
| Outdoor play | 12 | 1.23 |
| Physical behaviour | 10 | 1.02 |
| Tummy time | 9 | 0.92 |
| Sports | 8 | 0.82 |
| Standing | 8 | 0.82 |
| Moving | 6 | 0.61 |
| Stepping | 6 | 0.61 |
| Cycling | 4 | 0.41 |
| Outdoor playtime | 4 | 0.41 |
| Running | 4 | 0.41 |
| Energetic play | 3 | 0.31 |
| Active play | 2 | 0.20 |
| Active time | 2 | 0.20 |
| Indoor play | 2 | 0.20 |
| Leisure-time physical activity | 2 | 0.20 |
| Physical play | 2 | 0.20 |
| Active outdoor play | 1 | 0.10 |
| Active video gaming | 1 | 0.10 |
| Aerobic physical activity | 1 | 0.10 |
| Balance training | 1 | 0.10 |
| Being active | 1 | 0.10 |
| Biking | 1 | 0.10 |
| Climbing | 1 | 0.10 |
| Commuting | 1 | 0.10 |
| Crawling | 1 | 0.10 |
| Energetic physical activity | 1 | 0.10 |
| Exercise activity | 1 | 0.10 |
| Exercise capacity | 1 | 0.10 |
| Exercise-related behaviours | 1 | 0.10 |
| Functional training | 1 | 0.10 |
| Habitual physical activity | 1 | 0.10 |
| Household chores | 1 | 0.10 |
| Indoor hobbies | 1 | 0.10 |
| Light activity | 1 | 0.10 |
| Motor activity | 1 | 0.10 |
| Occupational and non-occupational physical activity | 1 | 0.10 |
| Outdoor activity | 1 | 0.10 |
| Outdoor hobbies | 1 | 0.10 |
| Physical activity time | 1 | 0.10 |
| Physical exertion | 1 | 0.10 |
| Physical fitness | 1 | 0.10 |
| Physical movement | 1 | 0.10 |
| Physically active play | 1 | 0.10 |
| Playing | 1 | 0.10 |
| Playing outdoors | 1 | 0.10 |
| Recreational activity | 1 | 0.10 |
| Recreational physical activity | 1 | 0.10 |
| Resistance training | 1 | 0.10 |
| Stair climbing | 1 | 0.10 |
| Upright | 1 | 0.10 |
| Walking activities | 1 | 0.10 |
| Work activity | 1 | 0.10 |
| **Sedentary behaviour terminology** | **n** | **%** |
| Sedentary behaviour | 884 | 90.30 |
| Screen time | 570 | 58.22 |
| Sedentary time | 488 | 49.85 |
| Sitting time | 173 | 17.67 |
| Sedentary activity | 108 | 11.03 |
| Physical inactivity | 90 | 9.19 |
| Lying time | 42 | 4.29 |
| Inactivity | 41 | 4.19 |
| Reclining | 14 | 1.43 |
| Rest | 9 | 0.92 |
| Sedentary screen time | 9 | 0.92 |
| Stationary time | 8 | 0.82 |
| Recreational screen time | 6 | 0.61 |
| Screen use | 6 | 0.61 |
| Restrained sitting | 4 | 0.41 |
| Stationary behaviour | 4 | 0.41 |
| Reading time | 3 | 0.31 |
| Restrained time | 3 | 0.31 |
| Passive transport | 2 | 0.20 |
| Screen behaviour | 2 | 0.20 |
| Screen viewing | 2 | 0.20 |
| Screen-based sedentary behaviour | 2 | 0.20 |
| Sedentarism | 2 | 0.20 |
| Sedentary lifestyle | 2 | 0.20 |
| Sedentary recreational screen time | 2 | 0.20 |
| Academic-related activities | 1 | 0.10 |
| Back time | 1 | 0.10 |
| Behaviours of sedentarity | 1 | 0.10 |
| Being sedentary | 1 | 0.10 |
| Carried | 1 | 0.10 |
| Cell time | 1 | 0.10 |
| Computer screen time | 1 | 0.10 |
| Daily leisure-sedentary behaviour time | 1 | 0.10 |
| Daily screen-sedentary behaviour time | 1 | 0.10 |
| Daily total screen duration | 1 | 0.10 |
| Inactive behaviour | 1 | 0.10 |
| Non-screen-based sedentary behaviour | 1 | 0.10 |
| Physically inactive | 1 | 0.10 |
| Quiet time | 1 | 0.10 |
| Recreational screen use | 1 | 0.10 |
| Recreational screen-based activity | 1 | 0.10 |
| Recreational sedentary-based sitting time | 1 | 0.10 |
| Screen viewing time | 1 | 0.10 |
| Seated transport | 1 | 0.10 |
| Sedentariness | 1 | 0.10 |
| Sedentarity | 1 | 0.10 |
| Sedentary | 1 | 0.10 |
| Sedentary bouts | 1 | 0.10 |
| Sedentary habits | 1 | 0.10 |
| Sedentary physical activity | 1 | 0.10 |
| Sedentary screen time activities | 1 | 0.10 |
| Sedentary screen-time | 1 | 0.10 |
| Sedentary screen-time behaviours | 1 | 0.10 |
| Sedentary state | 1 | 0.10 |
| Sedentary-related behaviour | 1 | 0.10 |
| Standing time | 1 | 0.10 |
| Stroller | 1 | 0.10 |
| Television screen time | 1 | 0.10 |
| Television viewing | 1 | 0.10 |
| Time spent restrained | 1 | 0.10 |
| Time spent sedentary | 1 | 0.10 |
| Video game | 1 | 0.10 |
| **Sleep terminology** | **n** | **%** |
| Sleep | 972 | 99.28 |
| Sleep duration | 706 | 72.11 |
| Sleep time | 460 | 46.99 |
| Bedtime or time in bed | 93 | 9.50 |
| Sleep behaviour | 10 | 1.02 |
| Nap | 7 | 0.72 |
| Sleep quantity | 4 | 0.41 |
| Sleep periods | 3 | 0.31 |
| Total sleep | 3 | 0.31 |
| Nap duration | 2 | 0.20 |
| Sleep hours | 2 | 0.20 |
| Time spent in sleep | 2 | 0.20 |
| Bedtime duration | 1 | 0.10 |
| Night rest | 1 | 0.10 |
| Sleep habits | 1 | 0.10 |
| Sleep length | 1 | 0.10 |
| Sleep per night | 1 | 0.10 |
