# Supplementary material for: Terminology for describing the 24-h cycle of physical activity, sedentary behaviour, and sleep: a scoping review
Source: Int J Behav Nutr Phys Act. 2026 Aug 5;23:107. doi: 10.1186/s12966-026-01945-6 (PMC13729492; doi:10.1186/s12966-026-01945-6)
Supplement: Supplementary file 5 [file 12966_2026_1945_MOESM5_ESM.docx]

**Supplementary Material S5.** Frequency of terms adopted to discuss sleep, sedentary behaviour, and physical activity collectively.

| **24-hour terminology reported (N=979)** | **Articles reporting** | **% of total articles (n/979)** |
| --- | --- | --- |
| Movement behaviours | 673 | 68.7 |
| 24-hour movement behaviours | 457 | 46.7 |
| Physical behaviours | 58 | 5.9 |
| 24-hour activity cycle | 36 | 3.7 |
| Time-use behaviours | 31 | 3.2 |
| Time-use compositions | 28 | 2.9 |
| 24-hour time-use compositions | 25 | 2.6 |
| 24-hour time-use | 24 | 2.5 |
| 24-hour physical behaviours | 15 | 1.5 |
| 24-hour behaviours | 14 | 1.4 |
| Lifestyle behaviours | 14 | 1.4 |
| 24-hour cycle | 13 | 1.3 |
| 24-hour activity behaviour | 12 | 1.2 |
| Time-use pattern | 12 | 1.2 |
| 24-hour activity behaviours | 11 | 1.1 |
| 24-hour time-use behaviours | 11 | 1.1 |
| 24-hour activity profile | 10 | 1.0 |
| 24-hour compositions | 10 | 1.0 |
| Movement-related behaviours | 10 | 1.0 |
| Physical activity behaviours | 10 | 1.0 |
| 24-hour activity compositions | 9 | 0.9 |
| 24-hour activity patterns | 9 | 0.9 |
| 24-hour movement | 9 | 0.9 |
| Daily movement behaviours | 9 | 0.9 |
| 24-hour activity | 8 | 0.8 |
| 24-hour movement patterns | 8 | 0.8 |
| Movement continuum | 8 | 0.8 |
| Time-use | 7 | 0.7 |
| 24-hour activities | 6 | 0.6 |
| 24-hour movement compositions | 6 | 0.6 |
| 24-hour time-use continuum | 6 | 0.6 |
| Activity behaviours | 6 | 0.6 |
| Daily behaviours | 6 | 0.6 |
| 24-hour movement continuum | 5 | 0.5 |
| Activity patterns | 5 | 0.5 |
| Daily activity behaviours | 5 | 0.5 |
| 24-hour daily cycle | 4 | 0.4 |
| 24-hour movement paradigm | 4 | 0.4 |
| 24-hour movement profiles | 4 | 0.4 |
| 24-hour time-use paradigm | 4 | 0.4 |
| Daily activities | 4 | 0.4 |
| Health behaviours | 4 | 0.4 |
| Movement compositions | 4 | 0.4 |
| Movement patterns | 4 | 0.4 |
| Time-use activity behaviours | 4 | 0.4 |
| Time-use components | 4 | 0.4 |
| 24-hour activity cycle behaviours | 3 | 0.3 |
| 24-hour behaviour | 3 | 0.3 |
| 24-hour behaviour profiles | 3 | 0.3 |
| 24-hour continuum | 3 | 0.3 |
| 24-hour movement behaviour patterns | 3 | 0.3 |
| Activity compositions | 3 | 0.3 |
| Daily time-use compositions | 3 | 0.3 |
| Rest-activity behaviours | 3 | 0.3 |
| 24-hour activity rhythm | 2 | 0.2 |
| 24-hour behaviour compositions | 2 | 0.2 |
| 24-hour behaviour patterns | 2 | 0.2 |
| 24-hour behavioural patterns | 2 | 0.2 |
| 24-hour daily activity behaviours | 2 | 0.2 |
| 24-hour integrated movement | 2 | 0.2 |
| 24-hour lifestyle behaviour | 2 | 0.2 |
| 24-hour movement activities | 2 | 0.2 |
| 24-hour movement framework | 2 | 0.2 |
| 24-hour movement pattern | 2 | 0.2 |
| 24-hour movement spectrum | 2 | 0.2 |
| 24-hour movement-related behaviours | 2 | 0.2 |
| 24-hour movements | 2 | 0.2 |
| 24-hour paradigm | 2 | 0.2 |
| 24-hour patterns | 2 | 0.2 |
| 24-hour rest-activity cycle | 2 | 0.2 |
| 24-hour time-use data | 2 | 0.2 |
| 24-hour use of time | 2 | 0.2 |
| Activity-sleep patterns | 2 | 0.2 |
| Daily activity compositions | 2 | 0.2 |
| Health-related behaviours | 2 | 0.2 |
| Movement activities | 2 | 0.2 |
| Movement behaviour continuum | 2 | 0.2 |
| Movement behaviour patterns | 2 | 0.2 |
| Time-use epidemiology | 2 | 0.2 |
| Time-use movement behaviours | 2 | 0.2 |
| 24-hour activity and sleep behaviours | 1 | 0.1 |
| 24-hour activity components | 1 | 0.1 |
| 24-hour activity continuum | 1 | 0.1 |
| 24-hour activity cycle outcomes | 1 | 0.1 |
| 24-hour activity cycle paradigm | 1 | 0.1 |
| 24-hour activity daily continuum | 1 | 0.1 |
| 24-hour activity domains | 1 | 0.1 |
| 24-hour activity metrics | 1 | 0.1 |
| 24-hour activity paradigm | 1 | 0.1 |
| 24-hour activity pattern | 1 | 0.1 |
| 24-hour activity spectrum | 1 | 0.1 |
| 24-hour activity-sleep profile | 1 | 0.1 |
| 24-hour behavioural time-use compositions | 1 | 0.1 |
| 24-hour combined movement behaviours | 1 | 0.1 |
| 24-hour components | 1 | 0.1 |
| 24-hour compositional activity | 1 | 0.1 |
| 24-hour compositions of activity behaviours | 1 | 0.1 |
| 24-hour compositions of behaviours | 1 | 0.1 |
| 24-hour compositions of physical behaviour | 1 | 0.1 |
| 24-hour cycle behaviours | 1 | 0.1 |
| 24-hour cycle of behaviours | 1 | 0.1 |
| 24-hour cycle of physical behaviours | 1 | 0.1 |
| 24-hour cycle of the day | 1 | 0.1 |
| 24-hour daily movements | 1 | 0.1 |
| 24-hour daily time distribution | 1 | 0.1 |
| 24-hour day compositions | 1 | 0.1 |
| 24-hour day paradigm | 1 | 0.1 |
| 24-hour distribution of movement behaviours | 1 | 0.1 |
| 24-hour framework | 1 | 0.1 |
| 24-hour free-living activity pattern | 1 | 0.1 |
| 24-hour free-living behaviours | 1 | 0.1 |
| 24-hour health-related behaviours | 1 | 0.1 |
| 24-hour healthy movement behaviours | 1 | 0.1 |
| 24-hour integrated activity | 1 | 0.1 |
| 24-hour integrated lifestyle behaviour | 1 | 0.1 |
| 24-hour lifestyle activities | 1 | 0.1 |
| 24-hour lifestyle behaviours | 1 | 0.1 |
| 24-hour motor behaviour | 1 | 0.1 |
| 24-hour movement and non-movement behaviours | 1 | 0.1 |
| 24-hour movement approach | 1 | 0.1 |
| 24-hour movement behaviour paradigm | 1 | 0.1 |
| 24-hour movement behavioural compositions | 1 | 0.1 |
| 24-hour movement combinations | 1 | 0.1 |
| 24-hour movement components | 1 | 0.1 |
| 24-hour movement compositions behaviours | 1 | 0.1 |
| 24-hour movement cycle | 1 | 0.1 |
| 24-hour movement profile | 1 | 0.1 |
| 24-hour movement-pattern | 1 | 0.1 |
| 24-hour movement/non-movement continuum | 1 | 0.1 |
| 24-hour movement/non-movement intensity continuum | 1 | 0.1 |
| 24-hour of behaviour | 1 | 0.1 |
| 24-hour of time-use | 1 | 0.1 |
| 24-hour rest-activity patterns | 1 | 0.1 |
| 24-hour rest-activity rhythm | 1 | 0.1 |
| 24-hour rhythms | 1 | 0.1 |
| 24-hour sleep-awake activity | 1 | 0.1 |
| 24-hour sleep-awake behaviours | 1 | 0.1 |
| 24-hour sleep-awake movement behaviours | 1 | 0.1 |
| 24-hour sleep-movement patterns | 1 | 0.1 |
| 24-hour sleep-wake behaviours | 1 | 0.1 |
| 24-hour sleep-wake cycle | 1 | 0.1 |
| 24-hour sleep-wake or activity cycle | 1 | 0.1 |
| 24-hour spectrum of behaviours | 1 | 0.1 |
| 24-hour time allocation patterns | 1 | 0.1 |
| 24-hour time budget | 1 | 0.1 |
| 24-hour time continuum | 1 | 0.1 |
| 24-hour time-use approach | 1 | 0.1 |
| 24-hour time-use distribution | 1 | 0.1 |
| 24-hour time-use of activities | 1 | 0.1 |
| 24-hour time-use profile | 1 | 0.1 |
| 24-time-use compositions | 1 | 0.1 |
| 24/7 physical behaviour | 1 | 0.1 |
| Activity balance | 1 | 0.1 |
| Activity behaviour patterns | 1 | 0.1 |
| Activity profile | 1 | 0.1 |
| Behaviour across the 24-hour period | 1 | 0.1 |
| Behaviour pattern | 1 | 0.1 |
| Behavioural domains | 1 | 0.1 |
| Behaviours across the movement spectrum | 1 | 0.1 |
| Circadian behaviours | 1 | 0.1 |
| Circadian rhythms of rest-activity and sleep pattern | 1 | 0.1 |
| Circadian sleep and activity behaviours | 1 | 0.1 |
| Circadian-related behaviours | 1 | 0.1 |
| Component of 24-hour time-use | 1 | 0.1 |
| Daily activity behaviour | 1 | 0.1 |
| Daily activity cycle | 1 | 0.1 |
| Daily movement behaviour patterns | 1 | 0.1 |
| Daily movement behaviour time-use compositions | 1 | 0.1 |
| Daily movement compositions | 1 | 0.1 |
| Daily movements | 1 | 0.1 |
| Daily time-use | 1 | 0.1 |
| Daily time-use behaviours | 1 | 0.1 |
| Habitual human behaviours | 1 | 0.1 |
| Habitual movement behaviours | 1 | 0.1 |
| Health-related time-use behaviours | 1 | 0.1 |
| Lifestyle activities | 1 | 0.1 |
| Lifestyle factors | 1 | 0.1 |
| Lifestyle patterns | 1 | 0.1 |
| Lifestyle-related behavioural variables | 1 | 0.1 |
| Modifiable lifestyle behaviours | 1 | 0.1 |
| Movement and play behaviours | 1 | 0.1 |
| Movement patterns across a 24-hour period | 1 | 0.1 |
| Movement-related activities | 1 | 0.1 |
| Movement-related time-use components | 1 | 0.1 |
| Movement/non-movement behaviours | 1 | 0.1 |
| Patterns of lifestyle behaviours | 1 | 0.1 |
| Patterns of time-use | 1 | 0.1 |
| Sleep-wake cycle | 1 | 0.1 |
| Temporal behaviour patterns | 1 | 0.1 |
| Time-codependent movement behaviours | 1 | 0.1 |
| Time-dependent behaviours | 1 | 0.1 |
| Time-use activities | 1 | 0.1 |
| Time-use behaviour profile | 1 | 0.1 |
| Time-use combination | 1 | 0.1 |
| Time-use components | 1 | 0.1 |
| Time-use compositional behaviours | 1 | 0.1 |
| Time-use data | 1 | 0.1 |
| Time-use in physical behaviours | 1 | 0.1 |
| Whole-day activity behaviour | 1 | 0.1 |
